# Development and validation of a new method for indirect estimation of neonatal, infant, and child mortality trends using summary birth histories

**DOI:** 10.1371/journal.pmed.1002687

**Published:** 2018-10-31

**Authors:** Roy Burstein, Haidong Wang, Robert C. Reiner, Simon I. Hay

**Affiliations:** Institute for Health Metrics and Evaluation, Department of Health Metrics Sciences, University of Washington, Seattle, Washington, United States of America; The Hospital for Sick Children, CANADA

## Abstract

**Background:**

The addition of neonatal (NN) mortality targets in the Sustainable Development Goals highlights the increased need for age-specific quantification of mortality trends, detail that is not provided by summary birth histories (SBHs). Several methods exist to indirectly estimate trends in under-5 mortality from SBHs; however, efforts to monitor mortality trends in important age groups such as the first month and first year of life have yet to utilize the vast amount of SBH data available from household surveys and censuses.

**Methods and findings:**

We analyzed 243 Demographic and Health Surveys (DHS) from 76 countries, which collected both complete and SBHs from 8.5 million children from 2.3 million mothers to develop a new empirically based method to indirectly estimate time trends in age-specific mortality. We used complete birth history (CBH) data to train a discrete hazards generalized additive model in order to predict individual hazard functions for children based on individual-, mother-, and country-year-level covariates. Individual-level predictions were aggregated over time by assigning probability weights to potential birth years from mothers from SBH data. Age-specific estimates were evaluated in three ways: using cross-validation, using an external database of an additional 243 non-DHS census and survey data sources, and comparing overall under-5 mortality to existing indirect methods.

Our model was able to closely approximate trends in age-specific child mortality. Depending on age, the model was able to explain between 80% and 95% of the variation in the validation data. Bias was close to zero in every age, with median relative errors spanning from 0.96 to 1.09. For trends in all under-5s, performance was comparable to the methods used for the Global Burden of Disease (GBD) study and significantly better than the standard indirect (Brass) method, especially in the 5 years preceding a survey. For the 15 years preceding surveys, the new method and GBD methods could explain more than 95% of the variation in the validation data for under-5s, whereas the standard indirect variants tested could only explain up to 88%. External validation using census and survey data found close agreement with concurrent direct estimates of mortality in the NN and infant age groups. As a predictive method based on empirical data, one limitation is that potential issues in these training data could be reflected in the resulting application of the method out of sample.

**Conclusions:**

This new method for estimating child mortality produces results that are comparable to current best methods for indirect estimation of under-5 mortality while additionally producing age-specific estimates. Use of such methods allows researchers to utilize a massive amount of SBH data for estimation of trends in NN and infant mortality. Systematic application of these methods could further improve the evidence base for monitoring of trends and inequalities in age-specific child mortality.

## Abstract 2

Roy Burstein and colleagues present and test their method for estimating age-specific child mortality using the summary birth history data that are broadly available from household surveys and censuses.

## Author summary

**Why was this study done?:**

- Recently, and particularly in light of the Sustainable Development Goals, there has been increased interest in rigorous measurement of neonatal and infant mortality trends at both national and subnational levels.
- Summary birth histories are a widely available data source for child mortality but have only been validated to produce estimates of under-5 mortality and are not widely used to make estimates of mortality for other age groups.
- A method was needed that would enable summary birth histories to be utilized in the estimation of age-specific (such as neonatal and infant) mortality trends.

**What did the researchers do and find?:**

- We extracted data from 243 Demographic and Health Surveys (DHS) that contained complete birth histories—data from which age-specific trends could be directly estimated and from which summary birth histories could be produced.
- We trained a discrete time survival model that could be used to predict mortality from summary birth histories and used both cross-validation and a systematic external validation on an additional database of 243 summary birth history–only census and survey sources in order to assess how well the model could reproduce age-specific mortality trends.
- We found that in both out-of-sample DHS data and on a large database of external data, model predictions of age-specific mortality closely matched validation data while also performing nearly as well as the current best model used for overall under-5 mortality.

**What do these findings mean?:**

- This method can help improve the empirical evidence base upon which global, national, and subnational neonatal, infant, and other age-specific child mortality estimates are made, including by making new use of publicly available summary birth history data on over 150 million children already extracted and analyzed for this paper.

## Introduction

Monitoring levels and trends of child mortality is a key component to understanding progress in child survival and for targeting additional policy and financial assistance to accelerate gains [1]. A complete, prospective, and continuous registration of births and deaths is the preferred source of information on child mortality [2], but in countries where child mortality is highest, deaths often go unrecorded because of poor or nonexistent vital registration (VR) systems [3]. In the absence of quality VR data, trends in under-5 mortality are typically estimated using retrospectively collected household sample survey and census data that ask mothers about births and deaths of their children [4,5].

Age-specific under-5 mortality varies widely both by and within country [4,6], and thus, it is critical to estimate levels and trends by age group with as much data as possible. The implications have high national and global relevance, particularly as the UN Sustainable Development Goals explicitly emphasized neonatal (NN) mortality in addition to under-5 mortality [7].

Household survey- and census-based child mortality questionnaires are available as either complete birth histories (CBHs), also sometimes known as full birth histories, or summary birth histories (SBHs). CBHs are preferred over SBHs because they capture detailed vital event histories on each child born to the surveyed mothers. Information on dates of birth and ages at death can thus be tabulated directly by age group and for specific years. In contrast, SBH surveys only ask each mother how many children she has birthed (children ever born [CEB]), how many of her children have died to date (children died [CD]), her age, and sometimes information about the time since first birth (TSFB) and/or marriage. Nevertheless, SBHs are widely available in many censuses and other sample surveys, in part because of the relative simplicity of collecting them. To utilize this vast source of data, several methods have been developed to indirectly estimate trends in under-5 mortality (5*q*0) from SBHs [8–11]. However, such methods have yet to be specifically adapted for wider application to estimate age-specific mortality among under-5s from SBHs; subsequently, past assessments of NN and infant mortality have been informed by comparably fewer data, especially outside of VR settings.

The Demographic and Health Surveys (DHS) have been widely collected in low- and middle-income countries (LMICs) since 1984 and provide a major source of CBH data. DHS surveys also collect SBH information, and the linked SBH–CBH serves as the basis for the method we describe in this paper. Other large survey families, such as the Multiple Indicator Cluster Surveys (MICS), collect SBHs in some settings and CBHs in others. Censuses are the largest global source of SBH data, representing data on many millions more children than are available in CBH surveys and often offering high sample sizes within small spatial areas. The DHS program takes steps to ensure high-quality and consistent data collection, including probing to ensure that CBH and SBH tabulations are aligned. Censuses have much more variation in collection methods and quality. For example, SBH modules are sometimes collected not from mothers but from household informants who are often male heads of household. Indirect trends in child mortality from SBHs are currently estimated using either the standard indirect method [8,11–15], a version of which is used by the UN Inter-Agency Groups for Child Mortality Estimation (IGME), or the combination of two methods outlined by Rajaratnam and colleagues [9], used in the Global Burden of Disease (GBD) study. For detailed review on these methods, see S1 Text. In brief, the standard indirect method uses simulated coefficients applied to the ratio of CD to CEB, aggregated at different maternal age (or TSFB) cohorts to estimate mortality rates and locate them in time. The GBD methods use pooled DHS survey data to inform two types of indirect estimation models, which are then combined to produce final estimates. The maternal age cohort (MAC)-based method is fundamentally similar to the standard indirect method. The maternal age period (MAP) method uses empirical distributions, tabulated from DHS CBH data, describing the proportion of children born as well as the proportion of CD to mothers of specific ages in each year preceding the survey. Separate MAP distributions are produced by maternal age, CEB, and region. The period-specific aggregations of expected CD and children born derived from these distributions are used to locate mortality risk in time in SBH data.

Other methods such as cohort change and birth history imputation have been proposed [10,16], but in general, the development of new methods for indirect estimation of age-specific mortality has been understudied. Furthermore, none of the major existing methods have explored the use of predictive covariates measured at the individual mother or child level. The continued investment in collection of DHS surveys over the past 30 years has provided a massive dataset in which both SBHs and CBHs are available and thus the opportunity to empirically train and test new methods.

In this paper, we describe and test a novel method for indirect estimation of age-specific mortality using SBHs, based on a discrete hazards survival analysis model. This approach differs from existing popular indirect methods in two main respects: it produces a cohesive set of age-specific trend estimates without reliance on model life tables, thus allowing for the flexibility to estimate mortality rates for younger age groups such as neonates, and it is fit and predicted at the individual level, utilizing time-varying individual covariates.

## Methods

### Data

We analyzed 243 DHS (https://dhsprogram.com/) surveys from 76 countries, collecting CBHs and SBHs on 8,504,688 children from 2,346,538 mothers. We included DHS surveys and related Macro Malaria Indicator Surveys conducted since 1988 and available by October 2017. A full listing of the surveys used with summary information can be found in the S1 Table.

Birth history data in DHS surveys are recorded as follows: women are asked a series of questions about how many sons and daughters they have given live births to, including how many live with them now and how many have died. Certain probing questions are included to get more accurate responses. These data are aggregated to CEB and CD, forming the SBH component of the data. CBHs are also collected for each child born to the mother. Month and year of birth are recorded, as is age if the child is still alive. If the child reporting on had died, age at death is recorded in days if the child was under 1 month at death, in months if the child was under 2 years at death, and in years if the child was 2 years or older at death.

We further analyzed an additional 243 censuses and household surveys from 93 LMICs in order to demonstrate how the method can be applied in datasets in which only SBH was collected and to validate our results against concurrent CBH data. Of the SBH-only sources used, 71 were census, 81 were UNICEF Multiple Indicator Cluster Surveys (MICS), and the rest were from other household survey families such as Living Standards Measurement Surveys and other country-specific household surveys. The DHS datasets, as well as an additional 99 other CBH data sources, were used for comparison.

To identify data sources, we searched the Global Health Data Exchange (GHDx, http://ghdx.healthdata.org/) for national census and survey data in LMICs with the following key words: complete birth history, summary birth history, child mortality, and infant mortality. This was further supplemented by bespoke searches on national statistics agency websites. We used only data sources for which individual-level data were available. A full listing of these data sources can be found in the S1 Table and S2 Table, along with their GHDx record identification number, where links to data distributors are provided.

### Statistical model

Our goal was to develop a model that could be used to predict age-specific trends in mortality using SBH data only. We used CBH data to train the model, since it allowed us to identify mortality risk in time and age. For independent variables, we only used attributes that were available from SBHs, since we ultimately wanted to use this model to estimate mortality trends in datasets in which only SBHs are available.

We treated data from CBH as time to event, or survival data [17,18]. The goal of survival modeling is to estimate the underlying hazard or survival functions that describe the risk of event over exposure time. Special care is taken for data that are right censored, for which event status is unobserved after a certain period. In the context of child mortality data, the “event” of interest is a death, “exposure time” is age since birth, and right censoring occurs when a child is reported alive.

Most survival models can be expressed in the general form *h*(*age*|*β****X***) = *h*_0,*age*_*e*^*β****X***^, where *h*_0_ represents the baseline hazard function over age, which is shifted by weighted effects of covariates ***X***. The baseline hazard function can be fit either parametrically to a variety of smooth functions defined either by probability distributions or as flexible splines [19] or discretely, either using arbitrary age bins or in data-defined age bins, as in the widely used Cox proportional hazards model. Covariates will generally shift the hazard function and as such have a proportional effect across ages. This proportionality can be relaxed using age-varying covariates.

For this analysis, we adopted a discrete time survival analysis (DTSA) [20] approach to modeling the baseline hazard function. In a DTSA model, age is split into discrete bins, which conforms well to the discrete nature of age reporting in CBH data. The baseline hazard function is flexibly parameterized using fixed effects dummies, *I*, for chosen discrete age bins. This is achieved by reshaping input data such that every row in the new dataset is associated with each age bin, *a*, entered into by each child, *i*, in the data. Censored age bins for any child are not included in the reshaped data. An indicator variable *Y*_*i*,*a*_ is included for each row and recorded as 1 if the child died in that age bin and 0 if they survived it. A no-covariate baseline hazard could then be determined by fitting the following logistic regression model:

$$Y_{i,a}∼Bernoulli(q_{a})$$

$$logit(q_{a})=\sum_{\alpha =1}^{A}I_{i,\alpha }\beta_{\alpha }$$

Note that fixed effects are estimated for each age bin without an intercept term so that each *β*_*a*_ is in reference to zero, and thus, each $e^{\beta_{a}}$ are interpretable as the probability of mortality in age group *a*, conditional on survival to age group *a*, or (*q*_*a*_). In the discrete case, we thus refer to *q*_*a*_ as the probability of death within age bin *a*, though “mortality rate” is commonly used interchangeably to describe the same quantity. This basic model can be extended to include individual-level covariates, random effects to account for hierarchical data, transformations or smoothing splines on covariates to improve prediction, and interactions with age bin dummies in order to allow for nonproportional effects of covariates.

**Fig 1:**
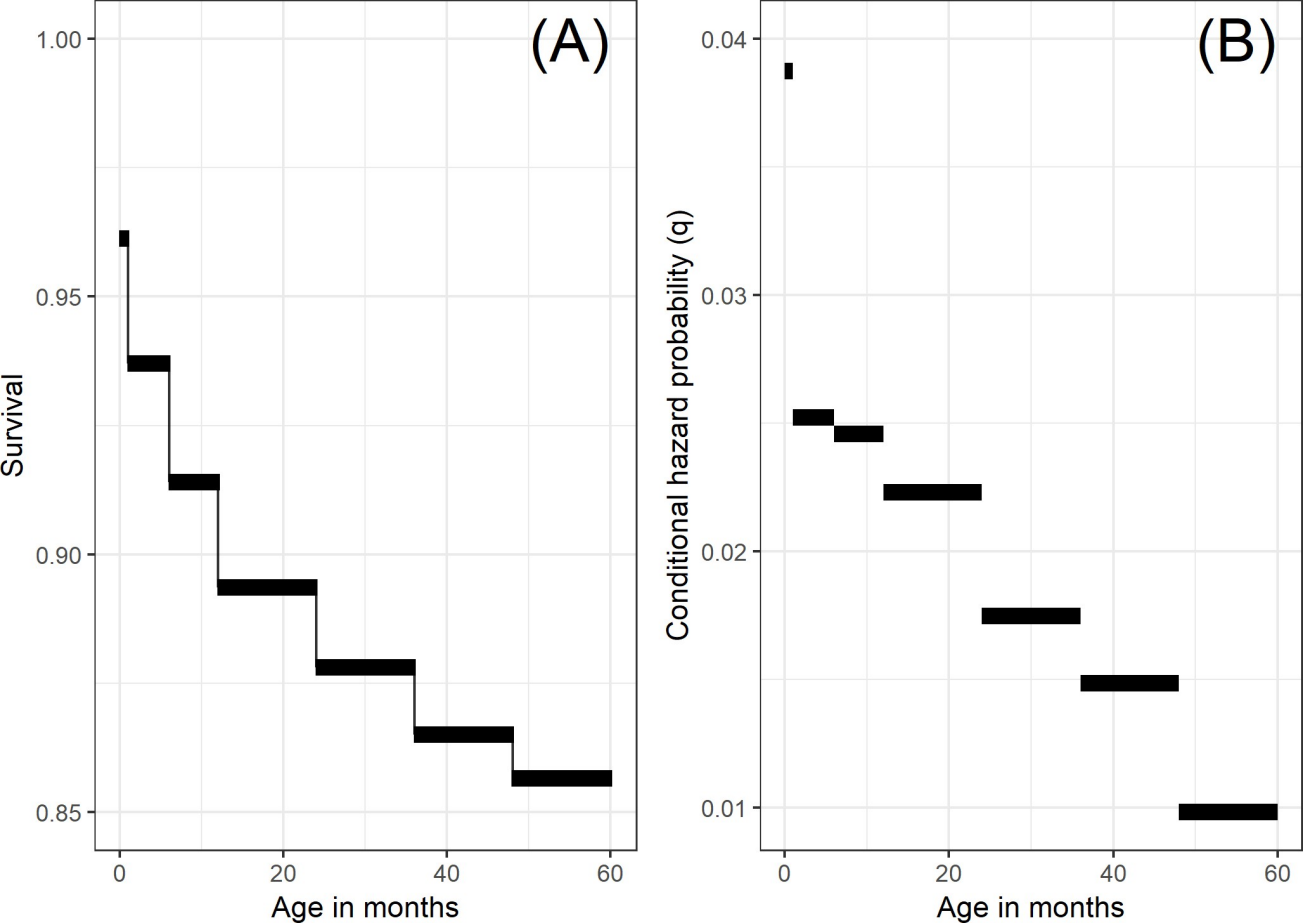
Illustrating the estimated pooled baseline discrete hazard and survival functions from the 2011 Burundi DHS dataset, fit using the seven age bins *a* ∈ (1 = *NN*,2 = *PNN*1,3 = *PNN*2,4 = 1*yr*,5 = 2*yr*,6 = 3*yr*,7 = 4*yr*). Note that we are estimating discrete hazards, and thus, hazards (shown in panel B) are interpreted as a conditional probability rather than a conditional rate. The survival function (shown in panel A), showing estimated survival at the end of each age bin, is calculated directly from estimated hazards as ${\hat{S}}_{a}=\prod_{\alpha =1}^{a}\left(1-q_{\alpha }\right)$. DHS, Demographic and Health Surveys; NN, neonatal; PNN1, post-neonatal 1; PNN2, post-neonatal 2.

For this application, we used the following seven age bins for this analysis: live birth to 28 days (NN), 29 days to 5 months inclusive (post-neonatal 1 [PNN1]), 6 to 11 months inclusive (post-neonatal 2 [PNN2]), 12 to 23 months inclusive (1yr), 24 to 35 months inclusive (2yr), 36 to 47 months inclusive (3yr), and 48 to 59 months inclusive (4yr). These bins were chosen to align with the way in which age information is collected in DHS such that each age bin would have identifiable data on children entering and dying within it. We separated the first year of life further into three age bins because there is a high and quickly changing mortality hazard during this period. The NN period during the first month of life is further split because it is often of separate public health interest because of the unique epidemiology of causes of death during this period. Fig 1 shows this simple baseline hazard function fit to the 2011 Burundi DHS dataset for illustration purposes.

We trained the model on the pooled CBH database with the purpose of making predictions in situations for which only SBHs are available, as in census data. As such, we were limited to using covariates from the training data, which were also available in SBH-only datasets. Certain covariates, such as year of birth and mother's age at birth, were found to be highly predictive of mortality but could not be ascertained directly from SBH data. In order to account for them, we approach predicting from the perspective of a hypothetical child. Specifically, for any given woman in the target SBH data, we wished to predict hazard functions for all hypothetical children she could have had over the course of her childbearing years. For example, if a 30-year-old woman was observed in a dataset collected in 2010, we could predict a separate hazard function for a potential child born to her each year going back until she was 12 in 1992. Hazard functions for these hypothetical children could be differentiated by covariate values that vary over the mother's life.

We specified the following generalized additive DTSA model for the conditional probability of death for every age bin *a* of each child *i* to each mother *m*:

$$Y_{m,i,a}∼Bernoulli(q_{m,i,a})$$

$$logit(q_{m,i,a})=\sum_{\alpha =1}^{7}[I_{i,\alpha }\beta_{\alpha }]+\sum_{\alpha =1}^{7}[g_{1,\alpha }(yr_{i},SDI_{c,yr,i})I_{i,\alpha }]+g_{2}(\frac{CD_{m}}{CEB_{m}},CEB_{m,yr},MothAge_{m,yr})+\nu_{svy}+\eta_{country,a}$$

$$\nu ∼Normal(0,\sigma_{\nu }^{2})$$

$$\eta ∼Normal(0,\sigma_{\eta }^{2})$$

where g_*_(∙) represents thin plate regression spline smooths, with *g*_1_(∙) having separate smooths for each age bin *a*, operationalized by the age bin dummy variable *I*. *yr*_*i*_ represents the year of birth for child *i*. This is directly observed in the training data but for prediction is assigned for each hypothetical child. *SDI*_*c*,*yr*,*i*_ represents the Socio-demographic Index (SDI) [21] for the country *c* that child *i* was born in at their year of birth *yr*. SDI is a composite average, expressed on a scale of 0 to 1, of income per capita, average educational attainment, and fertility rates and has been found to be a strong predictor of child mortality [21]. The interaction of SDI and year of birth allows the secular trend in mortality for each age bin to vary by the level of development in each country, thus allowing for prediction in countries without training data.

The variables in the second smooth represent child- and mother-level covariates. $\frac{CD_{m}}{CEB_{m}}$ is the ratio of CD to CEB to each mother *m* at the time of the survey. *MothAge*_*m*,*i*,*yr*_ is the mother's age at the year of birth. This is observed in the training data and assigned for prediction of hypothetical children in the same way as *yr*. Finally, *CEB*_*m*,*yr*_ is the number of children born to the mother at the time of birth *yr* of child *i*. This is directly observed in the training data. For prediction, we use empirical probability of birth distributions [9] to impute this value for each hypothetical child. Much in the same way that the standard indirect method interacts $\frac{CD}{CEB}$ with fertility ratios, this interaction is included to address the fact that the relationship between $\frac{CD}{CEB}$ and *q* is mediated by the fertility experiences of the women reporting $\frac{CD}{CEB}$ [15]. This differs from previous approaches, which used aggregate levels of fertility, and instead depends on individual women’s fertility experiences.

Finally, *ν* and *η* are independent normal random intercepts for each survey and each age bin within country.

All covariates were centered and scaled by their standard deviations for model fitting. Models were fit separately by the same regions used by Rajaratnam and colleagues [9]. Uncertainty in predictions was ascertained by taking 1,000 multivariate normal draws from the variance–covariance matrix of fitted model parameters, including fitted random effects values [22]. In cases for which prediction data had random effects levels not used in the training data (for a new survey or a new country), estimated variances $\hat{\sigma_{\nu }^{2}}$ and $\hat{\sigma_{\eta }^{2}}$ were used to simulate 1,000 independent normal draws. Models were fit using restricted maximum likelihood with the bam command from the mgcv package in the R Statistical Computing Language Version 3.4.3 [23,24].

### Conversion to trends

**Fig 2:**
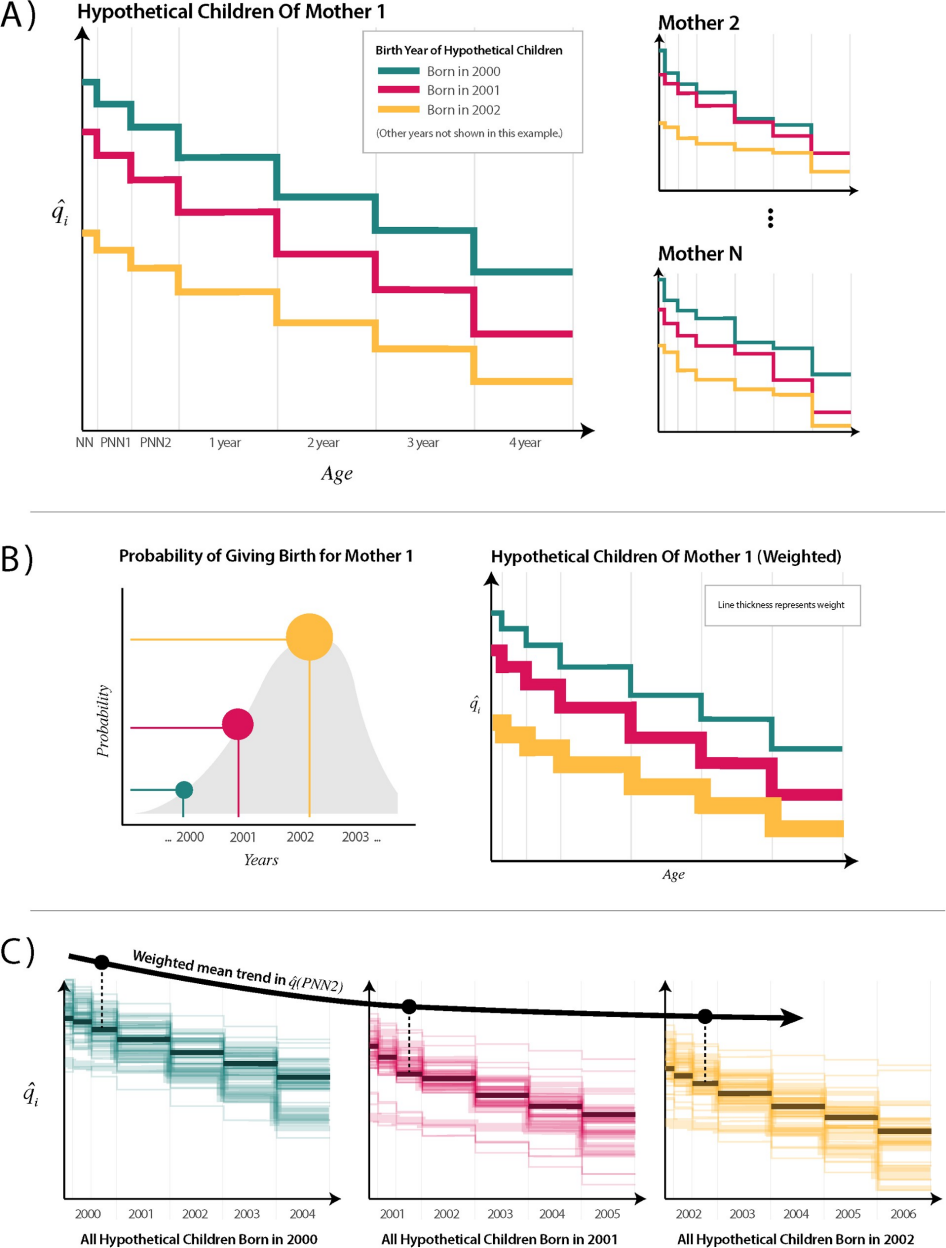
Illustration of procedure to convert discrete hazard functions for hypothetical children to population-level age-specific trends. (A) Discrete hazard functions are estimated for each hypothetical child from each mother in the target SBH dataset. Here, we color all children born in the same year with the same color. Only 3 years are shown for simplicity in this example. In real data, the years of birth of hypothetical children would vary by mother based on her age, such that there would be one hypothetical child for each year going back in time from the survey until the mother was 12 years old. (B) Probability of birth distributions is applied to each hypothetical birth from each mother. These are derived from the empirical map distributions from Rajaratnam and colleagues [9], in which a different probability is available by woman’s age, CEB, region of residence, and year prior to the survey. These probabilities are multiplied by each mother’s CEB and carried through to subsequent age bins to estimate the expected number of children entering each age bin (EEB) using estimated survival probabilities. As such, line thicknesses get slightly smaller with each subsequent age bin. The EEB value for each hypothetical child’s age bin represents the number of children entering that age bin that the hypothetical child represents for their given mother. (C) All hypothetical children to mothers are grouped by year of birth. The estimated mortality probabilities for each age bin from all hypothetical children born in the same year are pooled, and EEB is used to calculate a weighted mean. Trends are drawn across ${\hat{q}}_{a}$ for each year, indicated here by a trend in the third age bin. This aggregation procedure can be done for any grouping of women to make estimates for a survey cluster, a district, or a whole country. CEB, children ever born; EEB, expected entering bin; NN, neonatal; PNN1, post-neonatal 1; PNN2, post-neonatal 2; SBH, summary birth history.

Using the model described above, we estimated age-specific mortality hazards for individual hypothetical children to mothers responding to SBH questionnaires. These hazard functions of hypothetical children must then be converted into trends in age-specific mortality. To do so, we aggregated estimates of mortality among hypothetical children born in the period using weights that indicated the likelihood that each hypothetical child actually existed. This process is illustrated in Fig 2.

From the model, we obtained estimates of ${\hat{q}}_{m,a,yr}$: the probability of death in age bin *a* for a hypothetical child born in *yr* to mother *m*. To obtain estimates of ${\hat{q}}_{m,yr}$: age-bin and period-specific hazards representative of the population surveyed, we weighted each child based on their probability of birth. Each hypothetical child was assigned a probability of birth (*POB*_*m*,*yr*_) using the birth distributions used for the GBD-MAP method. Probability of birth distributions are compiled from empirical distributions that describe, for each year preceding a survey, the probability of birth based on mothers' age and CEB and by region. Distributions were matched based on geographical region, mothers’ age, CEB, and *yr* to each hypothetical child.

We then assigned a weight to each age bin of each hypothetical child. We defined the expected number of children entering each age bin (expected entering bin [EEB]) *a* for child born in year *yr* from mother *m* as the following:

$$EEB_{m,a,yr}=POB_{m,yr}*CEB_{m}*{\hat{S}}_{m,a,yr}$$

where ${\hat{S}}_{m,a,yr}$ is the estimated survival until age bin *a*, and *EEB* is the estimate of the number of children entering each age bin for the hypothetical child born to mother *m* in year *yr*, given each mother’s overall fertility and the estimated mortality experiences of her children over time.

We aggregated our estimates across ${\hat{q}}_{a,yr}$ by taking a weighted mean such that

$${\hat{q}}_{a,yr}=\frac{\sum_{m=1}^{M}{\hat{q}}_{m,a,yr}EEB_{m,a,yr}}{\sum_{m=1}^{M}EEB_{m,a,yr}}=\frac{Expected\;Deaths_{a,yr}}{Expected\;Children\;Entering_{a,yr}}.$$

The benefit of predicting at the individual level is that weighted means can be aggregated for any population desired. Also, this procedure conveniently provides not only estimates of ${\hat{q}}_{a,yr}$ and expected children entering each bin but also the numbers of expected deaths. For nationally representative estimates, survey weights can also be included into this procedure by multiplying weights into the summands. Finally, age bins can be combined as independent conditional probabilities to produce trends in wider age bins that may be of interest, such as $1\hat{q}0$ or $5\hat{q}0$.

Uncertainty in aggregate estimates of all quantities are calculated by repeating the aggregation procedure 1,000 times based on the predictive draws of ${\hat{q}}_{m,a,yr}$. We report the 2.5% and 97.5% quantiles.

### Validation and verification

We developed three approaches to model validation. We first used cross-validation on the DHS data in order to assess how well age-specific mortality trends estimated from our method could reproduce those directly estimated from CBH data. We then compared our indirect 5q0 estimates to those produced using existing methods. Finally, we applied the method to nationally representative non-DHS surveys that only collected SBHs and compared those results to contemporaneous direct estimates.

We developed the first model validation framework to assess out-of-sample predictive validity, holding out entire DHS surveys from the database, fitting the predictive model in their absence, and using their SBH variables to produce indirect age-specific time trends. We then used direct estimates from the CBHs of these held surveys to reproduce age-specific trends to serve as a basis for validation.

For each country in the DHS database, we held out the most recent DHS. We fit the model, used the fitted parameters to make indirect estimates from SBHs, and compared to direct estimates from CBHs. This was repeated for each country. Using the most recent survey represents a particularly difficult test because doing so requires several years of out-of-sample projection from the time since the penultimate survey in that country.

Our aim was to minimize the bias and magnitude of errors (the difference between estimates and validation data). We used the following five metrics to assess out-of-sample predictive performance: (1) Mean error (ME) to capture systematic bias. An ME of zero indicates a perfectly unbiased estimate. ME is an absolute (as opposed to relative) metric and thus cannot be compared across age bins. (2) Standard deviation of the errors (SDE) to capture how much variation there is in out-of-sample errors across countries and years. The smaller the SDE, the more precise the errors are. Again, SDE is an absolute metric. (3) Median relative error (MRE) to capture relative bias. MRE is simply the ratio of estimate to validation data, and as such, an MRE close to one indicates no bias. MRE allows us to compare bias on a relative scale across age bins. (4) Median absolute percentage error (MAPE) to capture the relative scale of errors. This is calculated as the ratio of the absolute error to the direct validation estimate multiplied by 100. The MAPE represents overall relative accuracy of the estimates, with a value close to zero indicating high accuracy. (5) The coefficient of determination (R^2^) represents the proportion of total variance in the directly tabulated hazards explained by the modeled estimates. Each of the metrics were assessed for each age bin as well as for 5*q*0.

Single-year age-specific direct tabulations of CBHs have relatively small sample sizes and can produce somewhat noisy estimates of a “truth” for comparison. Since we are interested in modeling the actual underlying trend and not the noisy observed values, very good predictive performance in this case could actually signal overfitting. Furthermore, mean-based metrics are sensitive to large outliers in errors, which could emerge spuriously when validation data are noisy. In other words, validation data with a larger sample size are expected to produce a precise approximation of the true underlying mortality hazard. We dealt with this in two ways. First, following Rajaratnam and colleagues [9], we smoothed the noisy validation trends using loess (with *α* set to 0.85). Second, we weighted all of our metrics of predictive performance by the sample sizes (number entering each age bin) as tabulated from the raw validation data.

We also used this same validation approach to evaluate our estimates of the numbers of children expected to enter each age bin, or EEB. This is done by comparing EEB with the direct tabulations of numbers of children entering each bin, in each year, from the validation data.

With increasing interest in subnational child mortality estimation [6,25–28], it is also critical to assess the validity of these results at subnational levels of aggregation. Most SBH data are geographically identifiable to the first administrative level—typically referred to as states or regions in most countries [6]. We aggregated to the first administrative unit, defined using the Global Administrative Unit Layers (GAUL) shape file made available by the FAO (http://www.fao.org/geonetwork/srv/en/metadata.show?id=12691). In order to obtain large enough sample sizes for stable comparison in the validation, we also aggregated data into 5-year bins preceding each survey. As such, each administrative area only supplied three estimates, and thus, we did not smooth them.

We also compared how well the proposed method estimated trends in 5*q*0 relative to existing methods, since a well-behaving method for age-specific trends should also be able to accurately reproduce trends in 5*q*0. We thus compared out-of-sample trends in 5*q*0 estimated from our test data to those produced by the GBD methods, as well as the standard indirect method. GBD-combined indirect estimates for each available survey were taken from the GBD mortality database (Available online: https://vizhub.healthdata.org/mortality/) and were produced by combining MAP and MAC estimates. For the standard indirect method, we matched model life tables to countries as used by IGME. We included two variants of the standard indirect method: one based on MACs and one based on TSFB (see [11]).

We also used this cross-validation framework to compare our model to several other model specifications. These results are presented in the S1 Fig.

Finally, in order to better establish external validity of this method, we also sought to understand its performance on non-DHS data. By nature of joint data collection, CBH and SBH data from DHS are presumed to be highly consistent. For this reason, SBHs from DHS could be different enough from data for which only SBHs are collected—for example, in censuses—that a cross-validation based on DHS alone may not be sufficient evidence that method performs well for these sources. Thus, for a more practical perspective on the performance of this method in settings where it is intended to be used (i.e., in data for which only SBHs were collected), we compared estimates from these data to directly estimated mortality for which concurrent CBH data were available. First, we applied our method to 243 nationally representative SBH surveys and censuses in order to estimate trends in NN, infant, and under-5 mortality. We then directly estimated these same trends from CBH surveys in the same countries, as described above for the DHS cross-validation, which served as a basis for comparison. We then identified CBH–SBH estimate pairs from the same country-years. Looking at the differences in these pairs of estimates, we used the same set of predictive validity metrics described for the cross-validation assessment in order to assess how similar the age-specific indirect estimates were to contemporaneous direct estimates. Furthermore, in order to understand how our model performed across different data source types, we stratified this comparison across MICS, censuses, and other survey sources.

### Data and code availability

All datasets used for this analysis are listed in the Supporting information (S1 Table for the DHS and S2 Table for additional surveys and censuses used for external validation). Each source in the tables is supplied with an ID number associated with a record in the GHDx (http://ghdx.healthdata.org/). Each GHDx record links to data providers for each survey.

All code for the analyses in this manuscript is available at https://github.com/royburst/sbh_agespecific_indirect_paper_code. In the near future, we plan to release a package for R that allows users to apply this indirect method to any SBH dataset.

## Results

Table 1 shows summary statistics from our cross-validation. The table shows the mean estimates of age-specific mortalities, *q*_*a*_, across all countries and age bins, along with aggregated out-of-sample predictive validity metrics for estimates of *q*_*a*_. We find little bias across all ages, as indicated by very small MEs and MREs close to one. The bias that does exist tends to be slightly over in the younger age bins and slightly under in the older age bins. We also see relatively small SDE across all age bins, indicating that, on average, there is a not large variation in out-of-sample errors across countries and years. Relative variance in errors, measured by MAPE, increases as *q*_*a*_ decreases as a function of age.

**Table 1:** Overall out-of-sample predictive validity metrics for each age bin and mean direct estimates of q_a_ across all country years in the Demographic and Health Surveys database for the 15 years prior to the survey being taken.

Age bin	${\bar{q}}_{a}$	ME	SDE	MRE	MAPE	R^2^
NN	0.031	0.0022	0.005	1.05	9.5%	0.82
PNN1	0.015	0.0010	0.004	1.09	15.9%	0.80
PNN2	0.013	0.0004	0.004	1.08	17.3%	0.82
1yr	0.013	0.0002	0.004	1.02	18.2%	0.88
2yr	0.009	−0.0001	0.002	1.00	16.2%	0.93
3yr	0.006	−0.0002	0.002	0.96	20.7%	0.88
4yr	0.003	−0.0001	0.001	0.97	20.6%	0.81
5q0	0.083	0.0033	0.010	1.05	8.8%	0.95

Abbreviations: 1yr, 12 to 23 months inclusive; 2yr, 24 to 35 months inclusive; 3yr, 36 to 47 months inclusive; 4yr, 48 to 59 months inclusive; MAPE, median absolute percentage error; ME, mean error; MRE, median relative error; NN, live birth to 28 days (neonatal); PNN1, 29 days to 5 months inclusive (post-neonatal 1); PNN2, 6 to 11 months inclusive (post-neonatal 2); $\bar{q_{a}}$, average estimated mortality probability; *R*^2^, coefficient of determination; SDE, standard deviation of the errors.

**Fig 3:**
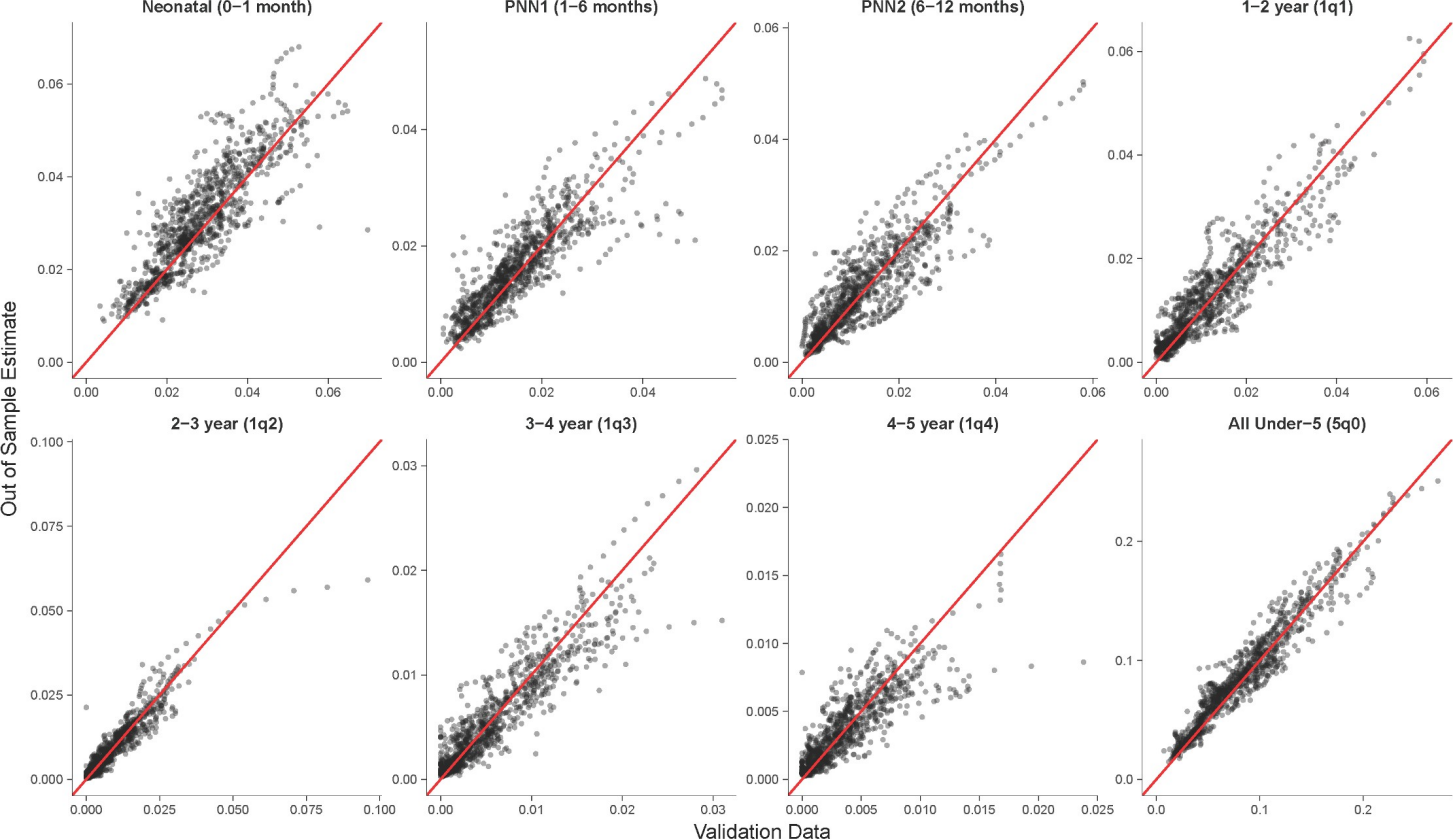
Out-of-sample predictions of mortality probability compared against loess-smoothed validation data. Each point represents a country–age mortality estimate (*q*_*a*,*yr*_) for each held-out survey from the DHS database. Red line indicates unity. DHS, Demographic and Health Surveys; loess, local regression; PNN1, post-neonatal 1; PNN2, post-neonatal 2.

Fig 3 plots the agreement between age-specific mortality rates from the validation data compared to out-of-sample estimates. We also see a relatively high proportion of variance explained as measure by *R*^2^, with all age bins above 0.80. Predictive validity metrics for the combined 5*q*0 age bin perform better than for the smaller age bins, as the model can explain 95% of the variance in input data. This is likely because of several reasons: errors are averaged over when collapsing across ages; relative metrics are less sensitive with a larger overall *q*_*a*_; age bins with larger relative errors tend to have lower hazards, which contribute less overall mortality and thus impact metrics in the combined group less; and larger sample sizes lead to more stable estimates.

**Fig 4:**
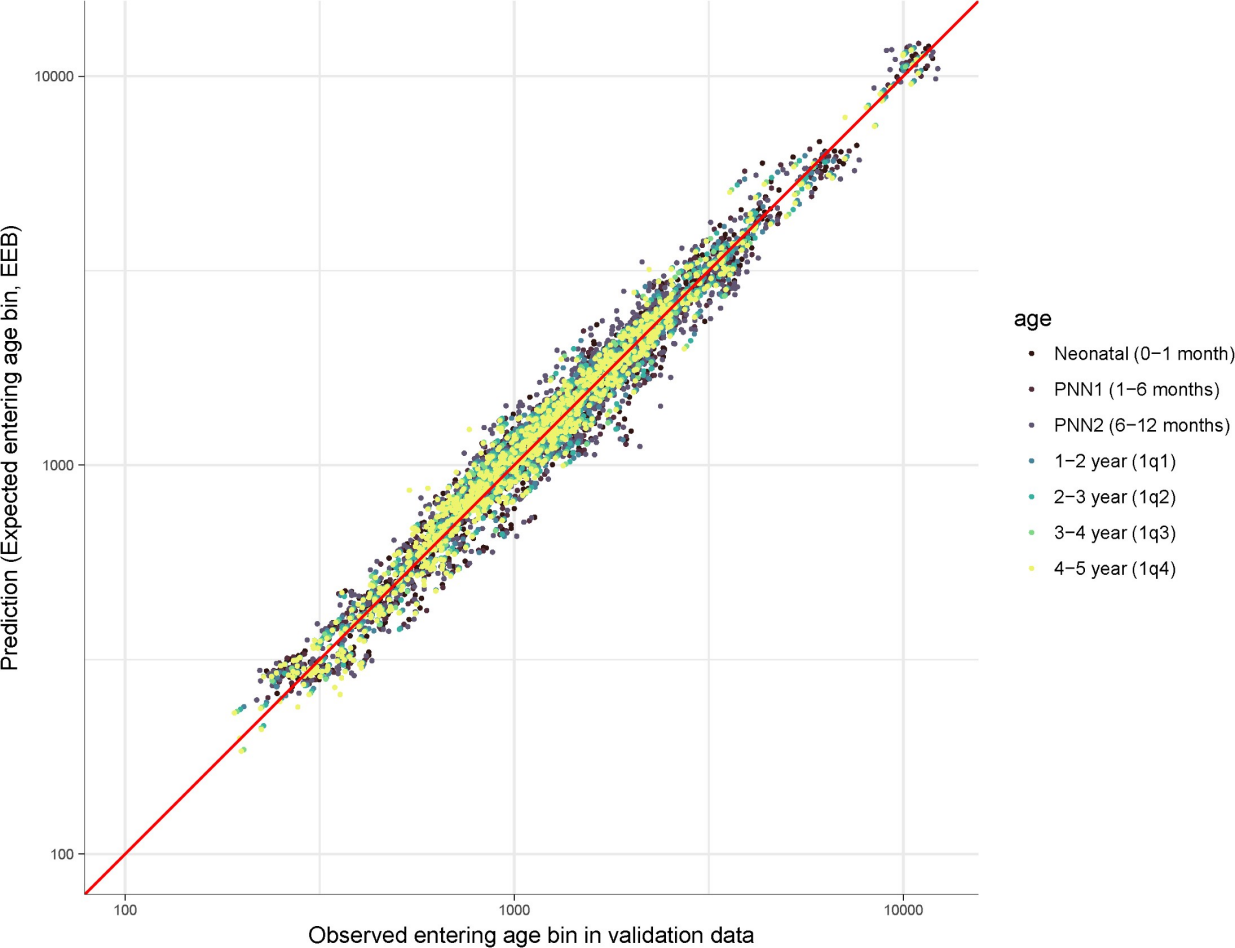
Comparison of EEB and observed children entering each age bin from CBH validation data. Each point represents a survey year age bin; both axes are on log 10 scale. *R*^2^ = 0.97. CBH, complete birth history; EEB, expected entering bin; PNN1, post-neonatal 1; PNN2, post-neonatal 2.

Fig 4 compares EEB with the observed number of children entering each age bin from the validation data. There was high agreement across age groups, with MRE ranging from 1.015 to 1.032 and MAPE ranging from 6.8% to 11.0%, indicating small errors and potentially a very slight upward bias in the EEB estimates. There is no clear difference in EEB performance across age bins. Overall, *R*^2^ was 0.97. This indicates that empirical probability of birth distributions can be reliably used to approximate sample sizes for indirect estimates. This also adds support to the favorable validation results shown above, as EEB weights are an important component of aggregating trends to the national level and because empirical probability of birth distributions are used to impute CEB at birth for prediction.

At the subnational level, model performance was somewhat weaker. There was a similar pattern in direction of bias across the ages, though bias remained minimal overall. There was more variability in the errors, with MAPE ranging from 20.0% in the NN group to 38.0% in 4-year-olds. The percentage of variance explained was also somewhat lower than at the national level. *R*^2^ of the subnational 5*q*0 estimates was 0.91. Some of this difference was likely due to smaller sample sizes in the subnational data compared to the national validation. Our validation data, which were based on direct estimates from CBH, represent realizations of the underlying probability, and thus, the empirical probability from the validation is measured with noise. Despite aggregating to 5-year bins, the average number of children born in each 5-year aggregated subnational observation was 520, compared to 1,769 in each annual national observation, and 4,148 (27%) of each survey-administrative area-age bin observation had no observed deaths. S2 Fig and S3 Table replicate Fig 3 and Table 1, respectively, at the first administrative subnational level.

**Fig 5:**
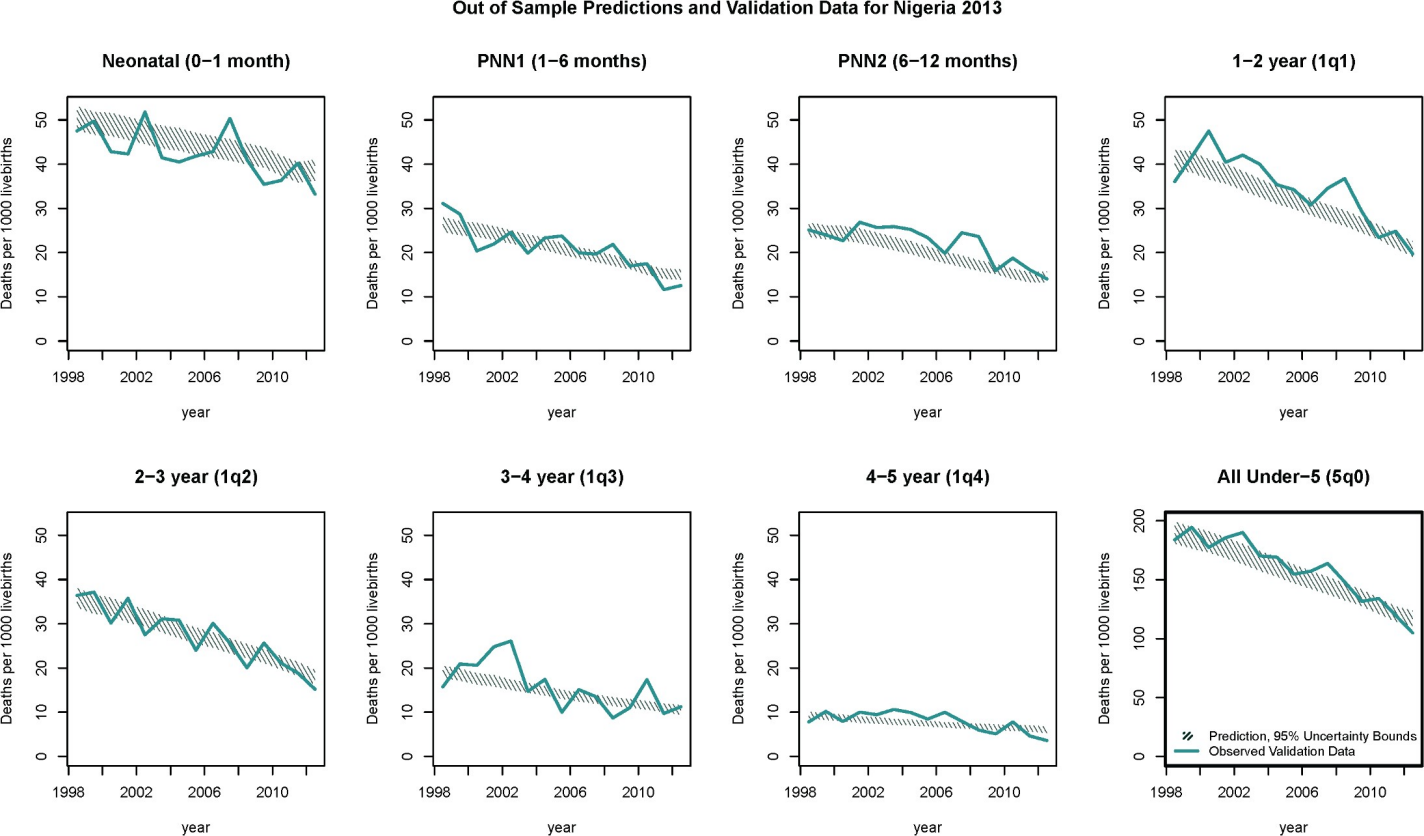
Trends in mortality for each age bin from the 2013 Nigeria DHS. Thick blue lines are validation data; hatched lines are the 95% uncertainty bounds on the out-of-sample predictions. Sampling variation is evident in the blue line through year-on-year spikes. The target of prediction was the overall time trend, leading to a smoother prediction. Axis scales are fixed except for 5q0, which is the combination of the mortality rates from the seven age bins. Similar plots for each country in the validation data are available in in the S4 Fig. DHS, Demographic and Health Surveys; PNN1, post-neonatal 1; PNN2, post-neonatal 2.

Fig 5 shows the out-of-sample estimated trends in age-specific mortality rates estimated using the 2013 Nigeria DHS and compared to the directly estimated validation data. In the S4 Fig, we provide similar plots for each country with extended discussion on those results. Overall, the model was able to reproduce trends in the validation data in Nigeria and in most other countries. Performance was suboptimal in cases for which test and train data differed significantly (for example, in Benin) and when trends were unique to a given country (for example, in Lesotho).

**Fig 6:**
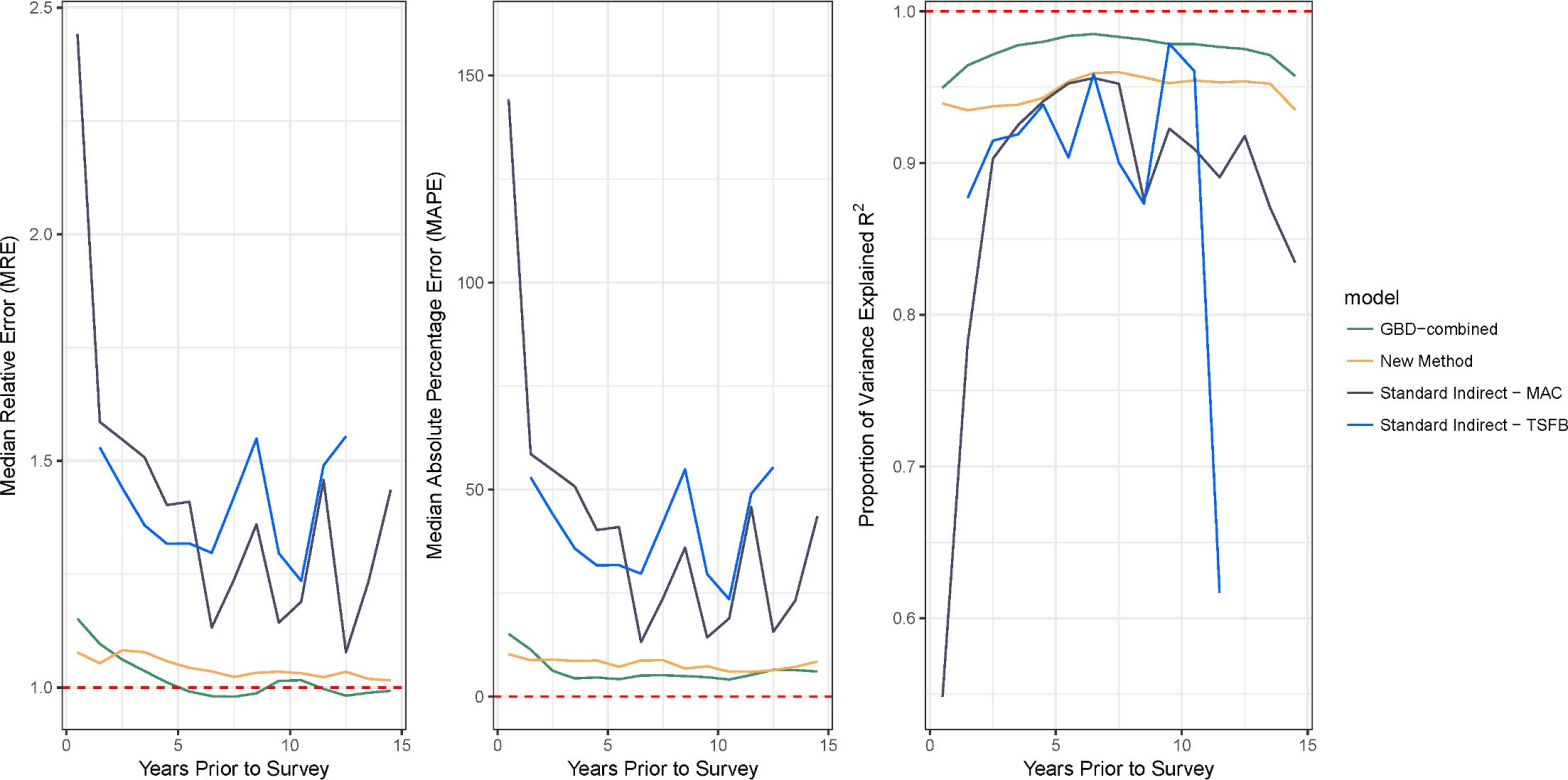
Comparing predictive validity metrics across different methods for indirect estimation of 5q0. Both the GBD-combined and new methods greatly outperform the standard indirect methods, particularly in the most recent 5 years. Note that whereas the standard indirect methods produce estimates in only a few years for any given survey, the location of these times vary based on the population in each survey as determined by the observed fertilities in each source, and thus, we are able to make annual estimates going back 15 years. GBD, Global Burden of Disease; MAC, maternal age cohort; TSFB, time since first birth.

We compared predictive validity in our out-of-sample estimates to indirect estimates of trends in 5*q*0 made from the same SBH holdout data, using the GBD-combined method and the standard indirect method. Fig 6 compares predictive validity metrics for the three methods over the 15 years preceding the survey. Confirming results from Rajaratnam and colleagues [9], we find unstable estimates from the standard indirect method in the most recent 5 years preceding surveys. Near overlap in the MRE and MAPE over time indicates that the new method and the GBD-combined methods generally produce similarly performing results. Overall, we estimated a MAPE of 8.8% for the new method, 6.1% for the GBD-combined method, and 25.6% and 36.5% for the MAC and TSFB variants of the standard indirect method, respectively. The new method and GBD-combined methods each had an MRE of 1.05 and 1.02, whereas the MAC and TSFB variants of the standard indirect method each had an MRE of 1.25 and 1.36. Finally, the *R*^2^ for the new method was 0.95, whereas it was 0.98 for the GBD-combined method and 0.80 for the MAC variant and 0.88 for the TSFB variance of the standard indirect method. If we excluded the most recent 5 years, MRE and MAP remained largely the same, but the *R*^2^ for the MAC and TSFB variants rose to 0.92 and 0.88, respectively. S3 Fig shows trends for each survey in the testing data. We note that for certain surveys with no GBD-combined estimates, such as the Malawi DHS 2016, we are able to produce accurate trends using the new method. It is possible that the non-GBD methods would have performed even better by comparison if these were included. Furthermore, several of the surveys in the testing set were used to train the GBD-combined models while remaining out of sample for the new method and the standard indirect method, potentially giving the GBD-combined method a slight advantage in this comparison.

For external validation, we identified 243 censuses and surveys from 93 countries, in which only SBH was collected. As a basis for comparison, we identified 316 CBH datasets. Applying our method, we estimated trends from each SBH-only data source and identified 16,527 estimate pairs for which we had contemporaneous SBH- and CBH-derived estimates in a single country-year. Estimates for any year after 1990 and within 15 years of the survey data were kept. We further identified 2,694 country-year–age pairs of data from 524 unique country-years for which only SBH data were available. For comparison, we also identified 10,655 country-year–age pairs for which two concurrent CBH direct estimates were available.

Full trend plots for each country with available data are in S6 Fig. In the majority of cases, trends from SBH-only data closely match contemporaneous trends from CBH data. There were several SBH-only surveys that exhibit overall source-level bias relative to concurrent trends.

Table 2 summarizes our findings for these paired comparisons for NN, infant (under-1), and under-5 mortality. We find close agreement across validation metrics. Overall variance was slightly higher and unadjusted *R*^2^ was slightly lower than in the cross-validation assessment. Much of this additional variance could be explained by survey; by simply controlling for data source using survey-level fixed effects, we find large improvements in *R*^2^, with each age bin around 0.96. We further found these results to be robust across SBH data type (census, MICS, and other surveys).

**Table 2:** Summary results for the external validation comparisons across 16,527 country-year data pairs for which a CBH and SBH estimate were both available. We also show a subanalysis by data source, indicating good robustness of results across source type.

Age bin	SBH source	${\bar{q}}_{a,cbh}$	${\bar{q}}_{a,sbh}$	ME	SDE	MRE	MAPE	R^2^	R^2^corr
NN	All	0.030	0.033	0.0026	0.010	1.06	17.7%	0.52	0.96
Infant	All	0.061	0.064	0.0030	0.018	1.05	16.4%	0.64	0.96
Under-5	All	0.093	0.096	0.0029	0.028	1.04	16.4%	0.74	0.97
NN	MICS	0.038	0.041	0.0030	0.009	1.09	17.9%	0.5	0.96
Infant	MICS	0.075	0.080	0.0047	0.019	1.05	16.1%	0.67	0.97
Under-5	MICS	0.123	0.129	0.0055	0.029	1.05	16.3%	0.79	0.98
NN	Census	0.030	0.032	0.0019	0.010	1.02	18.4%	0.55	0.96
Infant	Census	0.062	0.063	0.0013	0.019	1.00	17.2%	0.68	0.97
Under-5	Census	0.097	0.096	−0.0005	0.026	0.98	17.0%	0.79	0.98
NN	Other	0.029	0.032	0.0029	0.010	1.07	17.5%	0.48	0.96
Infant	Other	0.056	0.059	0.0034	0.017	1.07	15.8%	0.57	0.95
Under-5	Other	0.083	0.087	0.0037	0.028	1.06	16.2%	0.62	0.96

Abbreviations: CBH, complete birth history; MAPE, median absolute percentage error; ME, mean error; MICS, Multiple Indicator Cluster Surveys; MRE, median relative error; NN, neonatal; $\bar{q}$, average estimated mortality probability; *R*^2^, coefficient of determination; SBH, summary birth history; SDE, standard deviation of the errors.

**Fig 7:**
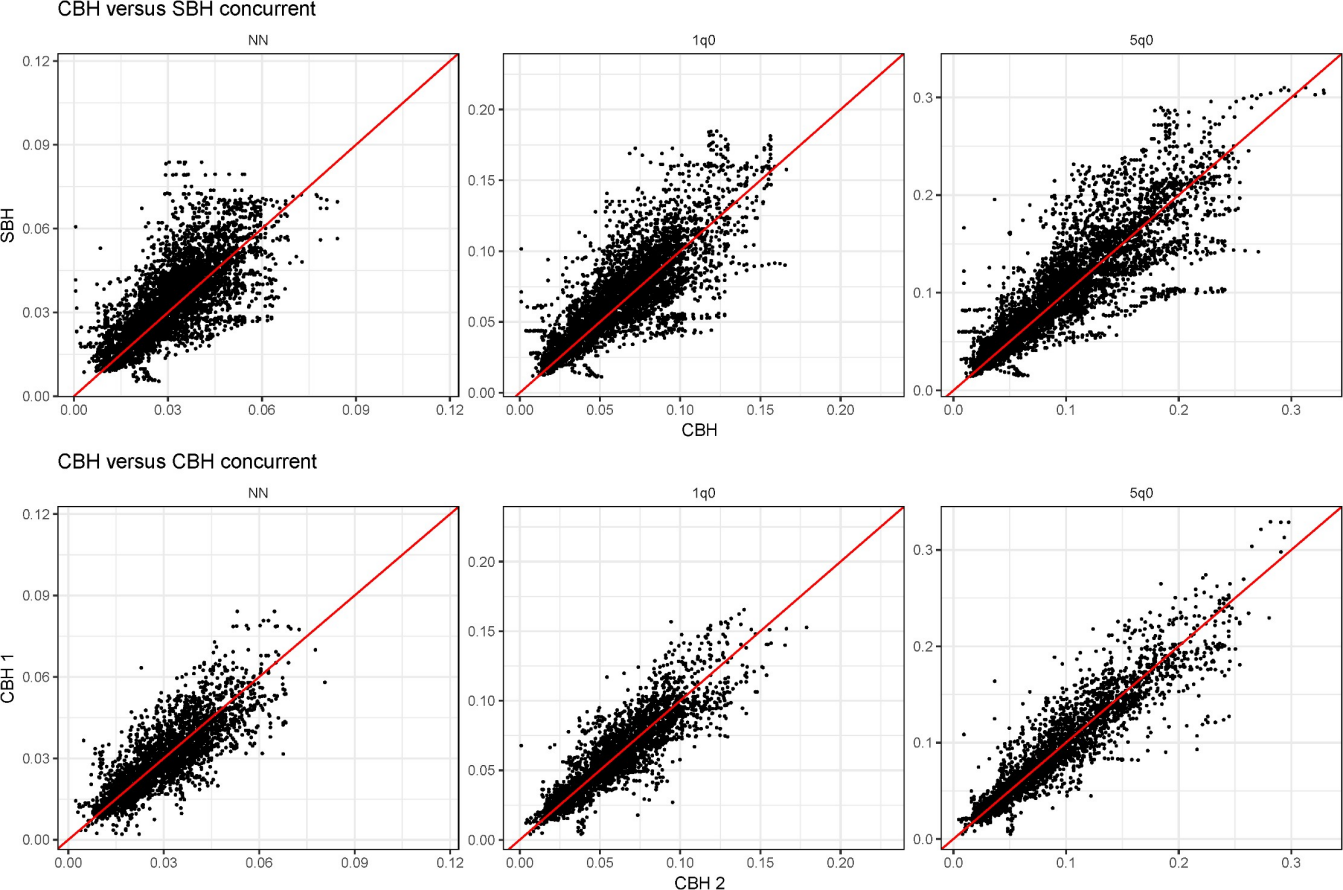
Each country-year concurrent estimate for NN, infant, and under-5 mortality. The top row compared concurrent estimates from the SBH-only data with CBH direct estimates. The bottom row shows the same comparison from concurrent CBH estimates, theoretically representing a baseline level of variance we would expect in concurrent estimates. Comparing red lines indicate unity. CBH, complete birth history; NN, neonatal; SBH, summary birth history.

Fig 7 shows a scatterplot of each country-year concurrent estimate. We also plot the same comparison for country-year pairs for which two direct CBH estimates are available. The comparison of CBH to CBH estimates represents a theoretical baseline difference we would expect to see in concurrent estimates. The similarity between the two sets of scatterplots highlights that much, though not all, of the variation we see between indirect and direct also exists between direct estimates and would be expected even given the best available survey data from which direct estimates could be made.

## Discussion

Our new method for indirect estimation produces age-specific mortality trends consistent with those produced using CBH data in most cases at the country- and first administrative unit–level, as well as producing 5*q*0 estimates that improve on the standard indirect method and are closely comparable in performance with the current best-performing method [9]. We applied the method to external SBH data and found considerable agreement when comparisons could be made to contemporaneous estimates from CBHs. This new method greatly expands the potential utility of SBH data and fills a critical gap in the literature on indirect methods, extending indirect mortality estimation toward specific age bins of interest.

There are two main methodological innovations introduced by this new approach: using hierarchical survival analysis to model individual-level hazard functions and developing a hybrid approach to locating mortality risk in time. By viewing CBHs as time-to-event data, we were able to directly model the quantity of interest, the conditional probability of death *q* at various ages from birth until age 5. Leveraging existing data from millions of CBHs, we inferred hazard functions that vary across countries, surveys, mothers, and their individual children using only covariates that were available in SBHs. These hazard functions, built up from flexibly chosen discrete age bins, then allowed us to produce indirect age-specific estimates for children born at various times. Since these estimates are made at the individual level, they could then be aggregated to any population. Furthermore, accompanying model uncertainty is included in all predictions.

All indirect methods must rely on some approximation in order to locate mortality risk in time, since SBH does not provide explicit information on time of birth or death. MAC methods such as GBD-MAC and the standard indirect approach rely on observed fertility patterns to locate the mean time of risk for each maternal age group. They typically assume unchanging fertility and furthermore ignore recent mortality experiences to children from older mothers. The GBD-MAP method relies on empirical distributions of births and deaths to distribute risks in terms of years prior to survey. This allows older mothers to contribute information from more recent births but also runs the danger of overgeneralizing trends to the level at which data were pooled. Our new method utilizes several sources of information in order to locate mortality risk and to overcome some of the limitations with previous methods. First, secular trends over time are incorporated in the model but are allowed to vary by country-SDI to avoid overgeneralization and allow for prediction in countries not in the training data. Second, individual-level time-varying covariates allow us to predict hazard functions for hypothetical children born throughout different times in each mother's life so that all potential children, including recent births to older women, are incorporated. In order to aggregate trends, we use weights derived for the GBD-MAP method, which put more weight on hypothetical children that were more likely to have existed.

In applying our method to a variety of SBH-only data sources, we found that performance varied across specific sources, and validation metrics in the external data were slightly worse than in the DHS cross-validation assessment. It could be argued that the utility of any indirect method will depend on the quality of SBH data collected [15]. Though to the contrary, indirect methods such as ours, which have been validated externally as well as against high-quality DHS data, can also serve as a tool to assess the quality of these data sources. For example, by comparing trends from multiple available sources within a country, as we show in supplementary Fig 5, certain sources stand out as problematic. See, for example, the upward trend apparent in the Ghana 2000 census or the downward shift seen in the 2008 Cambodia census. These two censuses suffer from different data quality issues, and a future research should focus on understanding the topology of potential issues in SBH data. Modeling groups such as IGME and GBD regularly exclude data sources because of quality concerns that arise in vetting. Furthermore, we found that much of the variation in the external validation could be explained using source-specific effects. In practice, the data-synthesizing models used by groups such as IGME and GBD can account for source-level biases using fixed or random effects.

As global child mortality has declined rapidly in recent years, it has become clear that improvements have not been equal across all ages in early childhood [21]. The Sustainable Development Goals now have an explicit target of reducing NN mortality to 12 deaths per 1,000 live births [7]. Until now, estimates of NN mortality have depended mostly on CBH data or VR, when it is available. If no data are available, estimates are completely modeled based on external information. Until complete and reliable VR data are available from all countries, SBH data should be considered an “inexpensive” alternative to costlier CBH surveys. As we have demonstrated through extensive and systematic external validation, this new method now opens the possibility of leveraging a huge amount of SBH data available from surveys and censuses for monitoring progress toward the NN mortality Sustainable Development Goal.

### Limitations and directions for future research

These results should be interpreted within the context of several limitations. First, despite being widely seen as high quality, and thus the basis for many child mortality estimates, DHS CBH data can suffer from certain issues such as selection biases [29] and misplacement of births [30]. By serving as the empirical basis upon which our model was trained, potential issues in these data could be reflected in the resulting application of it. Future research should focus on quantifying such issues and adjusting empirically based indirect methods to accommodate them. Second, the method presented here relies on formalizing existing relationships between covariates in the data to drive predictions. As such, when these relationships do not hold, predictions can suffer. Given the lack of period-based information in any one given SBH survey, it is expected that indirect estimates will poorly capture rapid changes in mortality [9]. This is partially mitigated in our approach by incorporating individual-level covariates, in which case mortality experiences from younger mothers will be more heavily weighted in recent periods. Third, by using GBD-MAP probability of birth distributions, we assume that fertility experiences are relatively stable over time among women in the same region, age, and number of CEB. Our preliminary analyses indicate this is generally true (see S7 Fig). Future research should focus on modeling these distributions at the individual level as well, potentially jointly fit within one model. Fourth, subnational predictions could likely be improved in the future by using subnational-level covariates rather than national-level covariates like SDI, as well as by implementing models that account for spatial autocorrelation in residuals. Fifth, by relying on concurrent SBH and CBH estimates as the basis for external validation, we could not ascertain the performance of this method in locations where only SBH exists, and thus, our sample may be somewhat biased toward higher-quality data. Finally, we validated the new model on one specific set of age bins, chosen to align with data collection and the typically used age breakdowns in previous research on child mortality. Future research can further validate other age bins and consider further distinguishing trends by sex.

## Conclusions

This new method introduces a novel approach to indirect estimation of child mortality. It produces results comparable to current best methods for indirect estimation of under-5 mortality while additionally producing age-specific estimates at both national and subnational levels, supplying researchers a tool with which to utilize a massive amount of SBH data for estimation of trends in NN and infant mortality at various geographic levels. Systematic application of these methods could further improve the evidence base for monitoring of trends and inequalities in age-specific child mortality.

## Supporting information

S1 Text A brief review of previous approaches to indirect methods for child mortality estimation using SBH data. SBH, summary birth history.
(DOCX)

S1 Table Input data sources for development and cross-validation of the method. This table lists each of the surveys used for training and testing the model. All were either DHS or related Malaria Indicator Surveys. Raw sample sizes for number of women and number of children are also given. More information on each survey, including download links, can be found by searching the GHDx ID at http://ghdx.healthdata.org/. The most recent survey for each country was used for validation and marked with an "X" in the table. DHS, Demographic and Health Surveys; GHDx, Global Health Data Exchange.
(DOCX)

S2 Table External validation data sources. An additional 342 surveys were used for external validation. Of these, 243 were SBH-only surveys; the rest were additional CBH sources used for comparison but not used in training the model. CBH, complete birth history; SBH, summary birth history.
(DOCX)

S3 TableOverall out-of-sample predictive validity metrics for each age bin and mean direct estimates of *q*_*a*_ across all first administrative unit years.MAPE, median absolute percentage error; ME, mean error; MRE, median relative error; $\bar{q}$, average estimated mortality probability; *R*^2^, coefficient of determination; SDE, standard deviation of the errors.(DOCX)

S1 Fig We used our validation framework to compare several model specifications. We tested and compared the following models: (FULL) The full model specification described in the Methods section. (INT) All covariates from the full model specified with no smooths; variable interactions in smooths replaced with interaction terms. (ADD) All covariates from the full model but specified with no smooths or interactions; all variables included additively and untransformed. (TREND) Only the year-SDI smooth included, with no individual covariates. (INDIV) Only the individual-level covariates used, with no year-SDI trend included. This figure shows metrics of predictive validity for these different specifications. The full model does reliably best across all metrics, with only a few exceptions. Notably, full specification is outperformed in bias (MRE) for several age groups by the interaction-only model. The full model generally outperforms all other models. These findings indicate that the predictive gains from using the more flexible (yet complex) GAM approach were appropriate here. The TREND specification does reliably worst across all metrics, indicating that there was value in including individual-level time-varying covariates. From left: MRE, MAPE, and *R*^2^. Dashed red lines are the target values for perfect fit for each metric. Note that if metrics for certain specifications are missing, they are outside of the plotted range and can be considered very poorly performing. GAM, generalized additive model; MAPE, median absolute percentage error; MRE, median relative error; SDI, Socio-demographic Index.
(TIF)

S2 Fig Out-of-sample predictions of mortality probability compared against loess-smoothed validation data at the first administrative level. Each point represents an admin1-age mortality estimate (*q*_*a*,*adm*1,*yr*_) for each held-out survey. Red line indicates unity. loess, local regression.
(TIF)

S3 Fig This figure shows estimated out-of-sample 5*q*0 trends for the 15 years preceding each survey in the test data, along with trends estimated via the GBD-combined and MAC and TSFB standard indirect methods. Sixty of the surveys had GBD-combined estimates available from the GBD mortality database. Trends begin at the year of the survey and go back 15 years or until 1990. Red lines indicate validation data (smoothed with loess, *α* = 0.85). GBD, Global Burden of Disease; loess, local regression; MAC, maternal age cohort; TSFB, time since first birth.
(TIF)

S4 Fig The figures (collectively S4 Fig) show estimated out-of-sample age-specific mortality trends with uncertainty compared to validation data for each country. Surveys used for out-of-sample validation are labeled with an “X” in Supplementary Table 1.
(DOCX)

S5 Fig The figures (collectively S5 Fig) show trends for each country from the external validation. Trends estimated using the new indirect method for SBH-only data are in blue; direct estimates from CBH surveys are in red. Citations for censuses and surveys used for external validation additional to the training and testing DHS data are listed in S2 Table. CBH, complete birth history; DHS, Demographic and Health Surveys; SBH, summary birth history.
(DOCX)

S6 Fig For a test of consistency within the external validation, we looked at the overall ratios of mortality rates across age bins of interest. In this figure, we show scatterplots of the relationships between NN and under-5 mortality, infant and under-5 mortality, and NN and infant mortality across country-year data pairs from contemporaneous SBH- and CBH-derived estimates. High overall agreement in these ratios adds further support to the predictive validity of this method. Each point is a contemporaneous country-year estimate. Red line indicates unity. CBH, complete birth history; NN, neonatal; SBH, summary birth history.
(TIF)

S7 Fig Using POB distributions from the GBD-MAP method has the limitation of being stationary in time. In other words, birth histories are pooled for all mothers from a region with the same CEB, irrespective of year of birth. If distributions changed significantly over time, this could present concern for this analysis. Using the DHS database, we reproduced POB distributions by region, maternal age, CEB, and year of survey (5-year increments). This figure shows two examples of empirical POB distributions made to explore changes in POB over time. Overall, we found very little change in POB distributions over time for most maternal age groups. The most change has occurred for low-fertility (1–2 CEB) older women, as the distributions seem to indicate there has been a trend toward delayed fertility in this group in recent years. Overall, this group represents a very small proportion of the mortality exposure in the data. Noisy distributions in high-fertility women are due to low sample sizes. (a) Sub-Saharan Africa West/Central region plots for women ages 30–31 years. (b) Asia region for women ages 46–47 years. A full set of distributions for all maternal ages and regions can be made available by the authors upon request. CEB, children ever born; DHS, Demographic and Health Surveys; GBD, Global Burden of Disease; MAP, maternal age period; POB, probability of birth.
(TIF)

## Abbreviations

CBH: complete birth history
CD: children died
CEB: children ever born
DHS: Demographic and Health Surveys
DTSA: discrete time survival analysis
EEB: expected entering bin
GAUL: Global Administrative Unit Layers
GBD: Global Burden of Disease Study
GHDx: Global Health Data Exchange
IGME: Inter-Agency Group for Child Mortality Estimation
LMIC: low- and middle-income country
MAC: maternal age cohort
MAP: maternal age period
MAPE: median absolute percentage error
ME: mean error
MICS: Multiple Indicator Cluster Surveys
MRE: median relative error
NN: neonatal
PNN1: post-neonatal 1
PNN2: post-neonatal 2
*R*^2^: coefficient of determination
SBH: summary birth history
SDE: standard deviation of the errors
SDI: Socio-demographic Index
TSFB: time since first birth
VR: vital registration
1yr: 12 to 23 months inclusive
2yr: 24 to 35 months inclusive
3yr: 36 to 47 months inclusive
4yr: 48 to 59 months inclusive
