# Supplementary material for: Development and validation of a new method for indirect estimation of neonatal, infant, and child mortality trends using summary birth histories
Source: PLoS Med. 2018 Oct 31;15(10):e1002687. doi: 10.1371/journal.pmed.1002687 (PMC6209133; doi:10.1371/journal.pmed.1002687)
Supplement: S1 Text — SBH, summary birth history. (DOCX) [file pmed.1002687.s001.docx]

# Review of previous indirect methods

Several popular methods have been developed to indirectly estimate $5q0$ and to locate this mortality risk in time. These methods are referenced in detail in the main manuscript and as such we provide a brief review of them here.

William Brass[1] laid the foundation for the first method to convert summary data on the total number of children born and died into a more conventional life-table measure, $xq0$, or the probability of death before reaching age $x$. Several refinements[2,3] have since been adopted since Brass's original work, which are widely used and well summarized in the UN's *Manual X: Indirect Techniques for Demographic Estimation*[4] and by Preston, Heuveline, and Guillot.[5] We refer to this as the standard indirect method.

Data requirements for the standard indirect method are the counts of number of children ever born ($CEB$) and the number of children died ($CD$) to mothers in 5-year age groups, beginning with 15-19 year olds. The ratio of $CD$ to $CEB$ for each maternal age group($\frac{CD_{i}}{CEB_{i}}$) is converted into a child-age ($x$) and period ($t$) specific mortality probability ($q$) using the following two equations:

$$xq0={(a}_{i}+b_{i} \frac{P_{1}}{P_{2}}+c_{i}\frac{P_{2}}{P_{3}})\frac{CD_{i}}{CEB_{i}}$$

$$t\left( x \right)=d_{i}+e_{i} \frac{P_{1}}{P_{2}}+f_{i}\frac{P_{2}}{P_{3}}$$

where $a_{i}$through $f_{i}$ are maternal age cohort specific coefficients estimated via simulation, and $P_{i}$are the average $CEB$ for each 5-year maternal age group. Each equation is used for each maternal age cohort, and each maternal age cohort produces estimates of cumulative mortality since birth, $xq0$, for a child age $x$ which is best identified for that age group of women. These estimates are then localized to a reference period $t(x)$, representing the year in which the estimate of $xq0$ most reliably refers. Estimates of mortality probabilities at specific ages $xq0$ are then converted to $5q0$ using model life tables. By locating risk from each maternal age cohort to one period in time, estimates of $5q0$ for the most recent periods come from mothers in the youngest age groups. These often do not represent overall mortality in the population because mortality to children of younger mothers is often higher.

The fertility ratios $\frac{P_{1}}{P_{2}}$ and $\frac{P_{2}}{P_{3}}$ used in the standard indirect method serve as an index for the earliness of fertility in the population. Fertility is critical to making accurate indirect estimates because it determines the amount of mortality risk (in time) children born to mothers of certain ages are exposed to. This is why the fertility ratios are multiplied by $\frac{CD_{i}}{CEB_{i}}$ in the first equation. By using survey-specific fertility ratios, however, the standard indirect method explicitly assumes that this period measure of fertility represents the fertility experience of all maternal cohorts in the data.

In 2010, Rajaratnam and colleagues[6] proposed a suite of new methods for indirect estimation using summary birth histories. A major advancement brought by their approach was to fit empirical models directly from data rather than relying on simulations. The authors used data from 166 DHS surveys, where $5q0$ could be estimated directly from $CBH$, and where aggregate measures of $CD_{i}$ and $CEB_{i}$ were also available for the same mothers. The new methods could broadly be categorized on the basis of estimation - either by maternal age cohort (MAC) or maternal age period (MAP). Variants based on time since first birth, rather than maternal age, were also tested, as this information is also available in some censuses.

Following the standard indirect method, the MAC method is also based on aggregate SBH information from 5-year age cohorts of women surveyed to localize $5q0$ estimates in time for each age group. Similar to the standard indirect method, the MAC method utilizes two models, one to estimate $q$ and another to estimate reference time. The equations differ from the standard indirect method in a number of key ways: $\frac{CD_{i}}{CEB_{i}}$ is logit transformed, $CEB_{i}$ is also included as a covariate, fertility ratios are included but not interacted with $\frac{CD_{i}}{CEB_{i}}$, and country random intercepts are included. Models are fit directly on DHS data using logit($5q0)$as the response, thus removing the need to rely on model life tables. By modeling cohort-specific $5q0$ directly, the MAC method resolves the young mother bias, but still relies on young mothers for more recent $5q0$ estimates and does not make use of information about more recent mortality of children from older mothers.

The maternal age period (MAP) method computes period-derived estimates based on frequency distributions from the same DHS database. Empirical distributions describing the proportion of children born as well as the proportion of children died to mothers in each year preceding the survey are tabulated from CBH data. Separate distributions are produced by maternal age and $CEB$ for each of five global regions. These distributions are then applied to each mother on the basis of her region, age, and $CEB$ in order to project back patterns of mortality risk and fertility likelihood, thus assigning each woman-year prior to the survey an expected number of births and deaths. These expected births and deaths are summed across the survey population by year and regressed on logit($5q0$) for that year. This generally yields coefficients near 1, indicating close agreement between the imputed annual $\frac{CD}{CEB}$ and observed logit($5q0$). The MAP method may over-generalize trends over time, since they are taken at the regional level. As such, individual and survey-level variation may not be well captured.

They then use loess smoothing to combine MAP, MAC, and their time since first birth variants to produce a combined result. Estimates resulting from this method are used for the Global Burden of Disease Study. We refer to this as the GBD-combined method. Using smoothed direct estimates from their DHS database as validation, they generally find large predictive improvements over the standard indirect approach, which has since been further verified by others.[7]

More recently Golding and Burstein, et al.[8] developed a method to approximate age-specific binomial samples for their mapping of child mortality in Africa. They again used a large training set of data where both CBH and SBH were available. Their method involved fitting two models, one to estimate a child-age and period-specific mortality probability, $q$, and another for the expected number of child months lived in the surveyed population for a specific child-age and period. Time lag and age indicator variables were used and interacted with logit($\frac{CD}{CEB}$) instead of fitting a separate reference time model in order to localize mortality risk in time, and variables for fertility ratios, year, and maternal age compositions were also included, along with country random effects. Reasonable predictive validity was achieved. Again, this method may also over generalize across time and space, since it relies on lag indicators and global coefficients for the other covariates. Furthermore, the model was fit and predicted on aggregate, rather than individual data.

Several other approaches to indirect estimation have also been described. For example, Kenneth Hill and colleagues[9,10] have proposed the cohort change and birth history imputation methods. The cohort change approach looks at change among true maternal cohorts between two surveys in order to estimate the $5q0$ for the inter-survey period. The birth history imputation approach involves imputing complete birth histories from summary birth histories, by using complete birth histories from a different survey in the same country and matching based on maternal characteristics. The birth history imputation approach has the benefit of producing age-period specific mortality estimates, though in their preliminary investigations, the authors report disappointing validation results.

# References

1. Brass W. Methods for Estimating Fertility and Mortality from Limited and Defective Data. North Carolina: Laboratories for Population Statistics, Univ. of NC; 1975.

2. Trussell TJ. A re-estimation of the multiplying factors for the Brass technique for determining childhood survivorship rates. Popul Stud. 1975;29: 97–107. doi:10.1080/00324728.1975.10410187

3. Feeney G. Estimating Infant Mortality Trends from Child Survivorship Data. Popul Stud. 1980;34: 109–128. doi:10.2307/2173698

4. Manual X [Internet]. [cited 5 Feb 2017]. Available: http://www.un.org/esa/population/publications/Manual_X/Manual_X.htm

5. Preston S, Heuveline P, Guillot M. Demography: Measuring and Modeling Population Processes. Wiley; 2000.

6. Rajaratnam JK, Tran LN, Lopez AD, Murray CJL. Measuring Under-Five Mortality: Validation of New Low-Cost Methods. PLOS Med. 2010;7: e1000253. doi:10.1371/journal.pmed.1000253

7. Verhulst A. Child mortality estimation: An assessment of summary birth history methods using microsimulation. Demogr Res. 2016;34: 1075–1128. doi:10.4054/DemRes.2016.34.39

8. Golding N, Burstein R, Longbottom J, Browne AJ, Fullman N, Osgood-Zimmerman A, et al. Mapping under-5 and neonatal mortality in Africa, 2000–15: a baseline analysis for the Sustainable Development Goals. The Lancet. 2017;390: 2171–2182. doi:10.1016/S0140-6736(17)31758-0

9. Hill K, Brady E, Zimmerman L, Montana L, Silva R, Amouzou A. Monitoring Change in Child Mortality through Household Surveys. PLOS ONE. 2015;10: e0137713. doi:10.1371/journal.pone.0137713

10. Brady E, Hill K. Testing survey-based methods for rapid monitoring of child mortality, with implications for summary birth history data. PLOS ONE. 2017;12: e0176366. doi:10.1371/journal.pone.0176366
