# Supplementary material for: Development and validation of a new method for indirect estimation of neonatal, infant, and child mortality trends using summary birth histories
Source: PLoS Med. 2018 Oct 31;15(10):e1002687. doi: 10.1371/journal.pmed.1002687 (PMC6209133; doi:10.1371/journal.pmed.1002687)
Supplement: S1 Table — This table lists each of the surveys used for training and testing the model. All were either DHS or related Malaria Indicator Surveys. Raw sample sizes for number of women and number of children are also given. More information on each survey, including download links, can be found by searching the GHDx ID at http://ghdx.healthdata.org/. The most recent survey for each country was used for validation and marked with an "X" in the table. DHS, Demographic and Health Surveys; GHDx, Global Health Data Exchange. (DOCX) [file pmed.1002687.s002.docx]

| **Model Region** | | **Country** | | **GHDx ID** | | **Survey** | | **Year** | | **# Mothers** | | **# Children** | **Test Data** | |
| --- | --- | --- | --- | --- | --- | --- | --- | --- | --- | --- | --- | --- | --- | --- |
| North Africa / Middle East | | Afghanistan | | 157018 | | MACRO_DHS | | 2016 | | 26,598 | | 125,715 | X | |
| Asia | | Albania | | 18834 | | MACRO_DHS | | 2009 | | 4,817 | | 12,766 | X | |
| Sub-Saharan Africa, West/Central | | Angola | | 56169 | | MACRO_MIS | | 2011 | | 6,517 | | 22,925 | X | |
| Asia | | Armenia | | 31750 | | MACRO_DHS | | 2010 | | 3,780 | | 8,424 | X | |
| Asia | | Armenia | | 18854 | | MACRO_DHS | | 2005 | | 4,276 | | 10,297 |  | |
| Asia | | Armenia | | 18843 | | MACRO_DHS | | 2000 | | 4,372 | | 11,286 |  | |
| Asia | | Bangladesh | | 157021 | | MACRO_DHS | | 2014 | | 16,079 | | 43,772 | X | |
| Asia | | Bangladesh | | 55956 | | MACRO_DHS | | 2012 | | 16,014 | | 45,834 |  | |
| Asia | | Bangladesh | | 18913 | | MACRO_DHS | | 2007 | | 9,849 | | 30,527 |  | |
| Asia | | Bangladesh | | 18902 | | MACRO_DHS | | 2004 | | 10,138 | | 33,597 |  | |
| Asia | | Bangladesh | | 26826 | | MACRO_DHS | | 2000 | | 9,353 | | 31,906 |  | |
| Asia | | Bangladesh | | 18878 | | MACRO_DHS | | 1997 | | 8,081 | | 29,344 |  | |
| Asia | | Bangladesh | | 18889 | | MACRO_DHS | | 1994 | | 8,541 | | 32,581 |  | |
| Sub-Saharan Africa, West/Central | | Benin | | 79839 | | MACRO_DHS | | 2012 | | 12,522 | | 47,152 | X | |
| Sub-Saharan Africa, West/Central | | Benin | | 18959 | | MACRO_DHS | | 2006 | | 13,814 | | 57,232 |  | |
| Sub-Saharan Africa, West/Central | | Benin | | 18950 | | MACRO_DHS | | 2001 | | 4,612 | | 19,398 |  | |
| Sub-Saharan Africa, West/Central | | Benin | | 18938 | | MACRO_DHS | | 1996 | | 1,804 | | 8,158 |  | |
| Latin America and the Caribbean | | Bolivia | | 19016 | | MACRO_DHS | | 2008 | | 11,720 | | 40,355 | X | |
| Latin America and the Caribbean | | Bolivia | | 18971 | | MACRO_DHS | | 1998 | | 7,634 | | 29,473 |  | |
| Latin America and the Caribbean | | Bolivia | | 18990 | | MACRO_DHS | | 1994 | | 6,053 | | 24,174 |  | |
| Latin America and the Caribbean | | Bolivia | | 18979 | | MACRO_DHS | | 1989 | | 5,542 | | 22,338 |  | |
| Sub-Saharan Africa, South/East | | Botswana | | 19019 | | MACRO_DHS | | 1988 | | 3,279 | | 10,670 | X | |
| Latin America and the Caribbean | | Brazil | | 19046 | | MACRO_DHS | | 1996 | | 8,390 | | 25,513 | X | |
| Sub-Saharan Africa, West/Central | | Burkina Faso | | 19133 | | MACRO_DHS | | 2011 | | 13,247 | | 56,178 | X | |
| Sub-Saharan Africa, West/Central | | Burkina Faso | | 19088 | | MACRO_DHS | | 2003 | | 9,474 | | 41,520 |  | |
| Sub-Saharan Africa, West/Central | | Burkina Faso | | 19076 | | MACRO_DHS | | 1999 | | 4,916 | | 22,145 |  | |
| Sub-Saharan Africa, West/Central | | Burkina Faso | | 19064 | | MACRO_DHS | | 1993 | | 4,778 | | 20,655 |  | |
| Sub-Saharan Africa, South/East | | Burundi | | 30431 | | MACRO_DHS | | 2011 | | 5,954 | | 24,520 | X | |
| Asia | | Cambodia | | 157024 | | MACRO_DHS | | 2014 | | 11,723 | | 33,290 | X | |
| Asia | | Cambodia | | 30379 | | MACRO_DHS | | 2011 | | 11,856 | | 37,511 |  | |
| Asia | | Cambodia | | 19167 | | MACRO_DHS | | 2006 | | 10,791 | | 40,457 |  | |
| Asia | | Cambodia | | 19156 | | MACRO_DHS | | 2000 | | 9,930 | | 40,990 |  | |
| Sub-Saharan Africa, West/Central | | Cameroon | | 19274 | | MACRO_DHS | | 2011 | | 11,023 | | 42,312 | X | |
| Sub-Saharan Africa, West/Central | | Cameroon | | 19211 | | MACRO_DHS | | 2004 | | 7,557 | | 29,455 |  | |
| Sub-Saharan Africa, West/Central | | Cameroon | | 19198 | | MACRO_DHS | | 1998 | | 3,847 | | 15,187 |  | |
| Sub-Saharan Africa, West/Central | | Cameroon | | 19188 | | MACRO_DHS | | 1991 | | 2,839 | | 11,612 |  | |
| Sub-Saharan Africa, West/Central | | Central African Republic | | 19292 | | MACRO_DHS | | 1995 | | 4,388 | | 16,936 | X | |
| Sub-Saharan Africa, West/Central | | Chad | | 157025 | | MACRO_DHS | | 2015 | | 14,156 | | 68,989 | X | |
| Sub-Saharan Africa, West/Central | | Chad | | 19315 | | MACRO_DHS | | 2004 | | 4,643 | | 21,448 |  | |
| Sub-Saharan Africa, West/Central | | Chad | | 19305 | | MACRO_DHS | | 1997 | | 5,865 | | 25,739 |  | |
| Latin America and the Caribbean | | Colombia | | 218566 | | MACRO_DHS | | 2016 | | 25,433 | | 62,580 | X | |
| Latin America and the Caribbean | | Colombia | | 19324 | | MACRO_DHS | | 2005 | | 26,536 | | 71,254 |  | |
| Latin America and the Caribbean | | Colombia | | 19359 | | MACRO_DHS | | 2000 | | 7,830 | | 21,267 |  | |
| Latin America and the Caribbean | | Colombia | | 19350 | | MACRO_DHS | | 1995 | | 7,500 | | 21,830 |  | |
| Latin America and the Caribbean | | Colombia | | 19341 | | MACRO_DHS | | 1990 | | 5,365 | | 15,964 |  | |
| Sub-Saharan Africa, South/East | | Comoros | | 76850 | | MACRO_DHS | | 2013 | | 2,934 | | 11,497 | X | |
| Sub-Saharan Africa, South/East | | Comoros | | 19370 | | MACRO_DHS | | 1996 | | 1,695 | | 7,913 |  | |
| Sub-Saharan Africa, West/Central | | Congo | | 56151 | | MACRO_DHS | | 2012 | | 8,787 | | 31,948 | X | |
| Sub-Saharan Africa, West/Central | | Congo | | 19391 | | MACRO_DHS | | 2005 | | 5,152 | | 16,687 |  | |
| Sub-Saharan Africa, West/Central | | Cote d'Ivoire | | 18533 | | MACRO_DHS | | 2012 | | 7,498 | | 28,211 | X | |
| Sub-Saharan Africa, West/Central | | Cote d'Ivoire | | 18531 | | MACRO_DHS | | 1999 | | 2,048 | | 7,575 |  | |
| Sub-Saharan Africa, West/Central | | Cote d'Ivoire | | 18519 | | MACRO_DHS | | 1994 | | 6,108 | | 24,870 |  | |
| Sub-Saharan Africa, West/Central | | Democratic Republic of the Congo | | 76878 | | MACRO_DHS | | 2014 | | 14,182 | | 59,276 | X | |
| Sub-Saharan Africa, West/Central | | Democratic Republic of the Congo | | 19381 | | MACRO_DHS | | 2007 | | 7,148 | | 29,548 |  | |
| Latin America and the Caribbean | | Dominican Republic | | 77819 | | MACRO_DHS | | 2013 | | 6,687 | | 18,167 | X | |
| Latin America and the Caribbean | | Dominican Republic | | 19456 | | MACRO_DHS | | 2007 | | 19,541 | | 58,037 |  | |
| Latin America and the Caribbean | | Dominican Republic | | 19444 | | MACRO_DHS | | 2002 | | 17,032 | | 53,667 |  | |
| Latin America and the Caribbean | | Dominican Republic | | 19431 | | MACRO_DHS | | 1999 | | 901 | | 2,871 |  | |
| Latin America and the Caribbean | | Dominican Republic | | 19421 | | MACRO_DHS | | 1996 | | 5,942 | | 19,784 |  | |
| Latin America and the Caribbean | | Dominican Republic | | 19410 | | MACRO_DHS | | 1991 | | 4,864 | | 17,163 |  | |
| North Africa / Middle East | | Egypt | | 154897 | | MACRO_DHS | | 2014 | | 19,770 | | 59,266 | X | |
| North Africa / Middle East | | Egypt | | 26842 | | MACRO_DHS | | 2008 | | 14,778 | | 48,619 |  | |
| North Africa / Middle East | | Egypt | | 19521 | | MACRO_DHS | | 2005 | | 17,552 | | 61,455 |  | |
| North Africa / Middle East | | Egypt | | 19529 | | MACRO_DHS | | 2003 | | 8,275 | | 30,298 |  | |
| North Africa / Middle East | | Egypt | | 19511 | | MACRO_DHS | | 2000 | | 14,164 | | 54,780 |  | |
| North Africa / Middle East | | Egypt | | 19493 | | MACRO_DHS | | 1996 | | 13,329 | | 56,390 |  | |
| North Africa / Middle East | | Egypt | | 19482 | | MACRO_DHS | | 1993 | | 8,983 | | 38,076 |  | |
| North Africa / Middle East | | Egypt | | 19472 | | MACRO_DHS | | 1989 | | 8,091 | | 35,519 |  | |
| Sub-Saharan Africa, South/East | | Eritrea | | 19539 | | MACRO_DHS | | 2002 | | 6,009 | | 24,370 | X | |
| Sub-Saharan Africa, South/East | | Ethiopia | | 218568 | | MACRO_DHS | | 2016 | | 10,274 | | 41,392 | X | |
| Sub-Saharan Africa, South/East | | Ethiopia | | 21301 | | MACRO_DHS | | 2011 | | 10,896 | | 45,540 |  | |
| Sub-Saharan Africa, South/East | | Ethiopia | | 19557 | | MACRO_DHS | | 2005 | | 9,339 | | 39,881 |  | |
| Sub-Saharan Africa, South/East | | Ethiopia | | 19571 | | MACRO_DHS | | 2000 | | 10,143 | | 44,174 |  | |
| Sub-Saharan Africa, West/Central | | Gabon | | 76706 | | MACRO_DHS | | 2012 | | 6,383 | | 23,109 | X | |
| Sub-Saharan Africa, West/Central | | Gabon | | 19579 | | MACRO_DHS | | 2001 | | 4,499 | | 16,878 |  | |
| Sub-Saharan Africa, West/Central | | Ghana | | 157027 | | MACRO_DHS | | 2014 | | 6,511 | | 23,118 | X | |
| Sub-Saharan Africa, West/Central | | Ghana | | 21188 | | MACRO_DHS | | 2008 | | 3,299 | | 11,888 |  | |
| Sub-Saharan Africa, West/Central | | Ghana | | 19627 | | MACRO_DHS | | 2003 | | 3,992 | | 15,086 |  | |
| Sub-Saharan Africa, West/Central | | Ghana | | 19614 | | MACRO_DHS | | 1999 | | 3,499 | | 13,188 |  | |
| Sub-Saharan Africa, West/Central | | Ghana | | 19604 | | MACRO_DHS | | 1994 | | 3,501 | | 13,280 |  | |
| Sub-Saharan Africa, West/Central | | Ghana | | 19587 | | MACRO_DHS | | 1988 | | 3,453 | | 14,216 |  | |
| Latin America and the Caribbean | | Guatemala | | 157031 | | MACRO_DHS | | 2015 | | 17,178 | | 55,398 | X | |
| Latin America and the Caribbean | | Guatemala | | 19656 | | MACRO_DHS | | 1999 | | 4,350 | | 18,581 |  | |
| Latin America and the Caribbean | | Guatemala | | 19637 | | MACRO_DHS | | 1995 | | 8,794 | | 38,753 |  | |
| Sub-Saharan Africa, West/Central | | Guinea | | 69761 | | MACRO_DHS | | 2012 | | 6,950 | | 27,683 | X | |
| Sub-Saharan Africa, West/Central | | Guinea | | 19683 | | MACRO_DHS | | 2005 | | 6,259 | | 27,115 |  | |
| Sub-Saharan Africa, West/Central | | Guinea | | 19670 | | MACRO_DHS | | 1999 | | 5,413 | | 22,943 |  | |
| Latin America and the Caribbean | | Guyana | | 21348 | | MACRO_DHS | | 2009 | | 3,484 | | 10,929 | X | |
| Latin America and the Caribbean | | Haiti | | 65118 | | MACRO_DHS | | 2012 | | 8,671 | | 29,013 | X | |
| Latin America and the Caribbean | | Haiti | | 19720 | | MACRO_DHS | | 2006 | | 6,547 | | 24,830 |  | |
| Latin America and the Caribbean | | Haiti | | 19708 | | MACRO_DHS | | 2000 | | 6,459 | | 26,437 |  | |
| Latin America and the Caribbean | | Haiti | | 19695 | | MACRO_DHS | | 1995 | | 3,288 | | 12,547 |  | |
| Latin America and the Caribbean | | Honduras | | 95440 | | MACRO_DHS | | 2012 | | 15,854 | | 49,263 | X | |
| Latin America and the Caribbean | | Honduras | | 19728 | | MACRO_DHS | | 2006 | | 13,991 | | 50,093 |  | |
| Asia | | India | | 19963 | | MACRO_DHS | | 2006 | | 84,609 | | 256,782 | X | |
| Asia | | India | | 19950 | | MACRO_DHS | | 2000 | | 80,872 | | 268,879 |  | |
| Asia | | India | | 19787 | | MACRO_DHS | | 1993 | | 79,322 | | 275,143 |  | |
| Asia | | Indonesia | | 76705 | | MACRO_DHS | | 2012 | | 32,129 | | 83,650 | X | |
| Asia | | Indonesia | | 20021 | | MACRO_DHS | | 2007 | | 30,420 | | 84,726 |  | |
| Asia | | Indonesia | | 20011 | | MACRO_DHS | | 2003 | | 27,317 | | 79,791 |  | |
| Asia | | Indonesia | | 19999 | | MACRO_DHS | | 1997 | | 26,562 | | 86,276 |  | |
| Asia | | Indonesia | | 19990 | | MACRO_DHS | | 1994 | | 26,045 | | 90,326 |  | |
| Asia | | Indonesia | | 19979 | | MACRO_DHS | | 1991 | | 21,065 | | 74,329 |  | |
| North Africa / Middle East | | Jordan | | 77517 | | MACRO_DHS | | 2012 | | 10,304 | | 42,275 | X | |
| North Africa / Middle East | | Jordan | | 21206 | | MACRO_DHS | | 2009 | | 9,124 | | 38,199 |  | |
| North Africa / Middle East | | Jordan | | 20083 | | MACRO_DHS | | 2007 | | 9,916 | | 43,460 |  | |
| North Africa / Middle East | | Jordan | | 20073 | | MACRO_DHS | | 2002 | | 5,494 | | 25,296 |  | |
| North Africa / Middle East | | Jordan | | 20060 | | MACRO_DHS | | 1997 | | 5,038 | | 24,243 |  | |
| North Africa / Middle East | | Jordan | | 20051 | | MACRO_DHS | | 1990 | | 5,853 | | 32,812 |  | |
| Asia | | Kazakhstan | | 20103 | | MACRO_DHS | | 1999 | | 3,364 | | 8,106 | X | |
| Asia | | Kazakhstan | | 20092 | | MACRO_DHS | | 1995 | | 2,649 | | 6,866 |  | |
| Sub-Saharan Africa, South/East | | Kenya | | 157057 | | MACRO_DHS | | 2014 | | 23,245 | | 83,591 | X | |
| Sub-Saharan Africa, South/East | | Kenya | | 21365 | | MACRO_DHS | | 2009 | | 6,102 | | 22,534 |  | |
| Sub-Saharan Africa, South/East | | Kenya | | 20145 | | MACRO_DHS | | 2003 | | 5,865 | | 22,074 |  | |
| Sub-Saharan Africa, South/East | | Kenya | | 20132 | | MACRO_DHS | | 1998 | | 5,717 | | 23,351 |  | |
| Sub-Saharan Africa, South/East | | Kenya | | 20120 | | MACRO_DHS | | 1993 | | 5,415 | | 23,899 |  | |
| Sub-Saharan Africa, South/East | | Kenya | | 20109 | | MACRO_DHS | | 1989 | | 5,507 | | 25,173 |  | |
| Asia | | Kyrgyzstan | | 77518 | | MACRO_DHS | | 2012 | | 5,601 | | 16,180 | X | |
| Asia | | Kyrgyzstan | | 20154 | | MACRO_DHS | | 1997 | | 2,776 | | 8,781 |  | |
| Sub-Saharan Africa, South/East | | Lesotho | | 157058 | | MACRO_DHS | | 2014 | | 4,540 | | 11,710 | X | |
| Sub-Saharan Africa, South/East | | Lesotho | | 21382 | | MACRO_DHS | | 2010 | | 5,191 | | 14,429 |  | |
| Sub-Saharan Africa, South/East | | Lesotho | | 20167 | | MACRO_DHS | | 2005 | | 4,832 | | 14,708 |  | |
| Sub-Saharan Africa, West/Central | | Liberia | | 77385 | | MACRO_DHS | | 2013 | | 7,559 | | 30,804 | X | |
| Sub-Saharan Africa, West/Central | | Liberia | | 34279 | | MACRO_MIS | | 2009 | | 3,643 | | 14,872 |  | |
| Sub-Saharan Africa, West/Central | | Liberia | | 20191 | | MACRO_DHS | | 2007 | | 5,701 | | 22,123 |  | |
| Sub-Saharan Africa, South/East | | Madagascar | | 21409 | | MACRO_DHS | | 2009 | | 12,970 | | 48,464 | X | |
| Sub-Saharan Africa, South/East | | Madagascar | | 20223 | | MACRO_DHS | | 2004 | | 5,845 | | 20,799 |  | |
| Sub-Saharan Africa, South/East | | Madagascar | | 20212 | | MACRO_DHS | | 1997 | | 5,233 | | 21,654 |  | |
| Sub-Saharan Africa, South/East | | Madagascar | | 20202 | | MACRO_DHS | | 1992 | | 4,369 | | 18,931 |  | |
| Sub-Saharan Africa, South/East | | Malawi | | 218581 | | MACRO_DHS | | 2016 | | 18,988 | | 68,074 | X | |
| Sub-Saharan Africa, South/East | | Malawi | | 21393 | | MACRO_DHS | | 2010 | | 18,041 | | 72,301 |  | |
| Sub-Saharan Africa, South/East | | Malawi | | 20263 | | MACRO_DHS | | 2005 | | 9,298 | | 35,883 |  | |
| Sub-Saharan Africa, South/East | | Malawi | | 20252 | | MACRO_DHS | | 2000 | | 10,337 | | 40,421 |  | |
| Sub-Saharan Africa, South/East | | Malawi | | 20235 | | MACRO_DHS | | 1992 | | 3,718 | | 16,330 |  | |
| Asia | | Maldives | | 21311 | | MACRO_DHS | | 2009 | | 6,106 | | 20,136 | X | |
| Sub-Saharan Africa, West/Central | | Mali | | 77388 | | MACRO_DHS | | 2013 | | 8,480 | | 33,803 | X | |
| Sub-Saharan Africa, West/Central | | Mali | | 20274 | | MACRO_DHS | | 2006 | | 11,566 | | 52,140 |  | |
| Sub-Saharan Africa, West/Central | | Mali | | 20315 | | MACRO_DHS | | 2001 | | 10,379 | | 48,407 |  | |
| Sub-Saharan Africa, West/Central | | Mali | | 20301 | | MACRO_DHS | | 1996 | | 7,935 | | 37,921 |  | |
| Sub-Saharan Africa, West/Central | | Mauritania | | 20322 | | MACRO_DHS | | 2001 | | 4,584 | | 19,202 | X | |
| North Africa / Middle East | | Morocco | | 20361 | | MACRO_DHS | | 2004 | | 8,660 | | 32,494 | X | |
| North Africa / Middle East | | Morocco | | 20371 | | MACRO_DHS | | 1992 | | 4,986 | | 22,657 |  | |
| Sub-Saharan Africa, South/East | | Mozambique | | 55975 | | MACRO_DHS | | 2011 | | 10,624 | | 37,984 | X | |
| Sub-Saharan Africa, South/East | | Mozambique | | 20394 | | MACRO_DHS | | 2004 | | 9,732 | | 37,443 |  | |
| Sub-Saharan Africa, South/East | | Mozambique | | 20382 | | MACRO_DHS | | 1997 | | 6,798 | | 25,752 |  | |
| Asia | | Myanmar | | 157061 | | MACRO_DHS | | 2016 | | 7,796 | | 22,989 | X | |
| Sub-Saharan Africa, South/East | | Namibia | | 150382 | | MACRO_DHS | | 2013 | | 6,453 | | 18,090 | X | |
| Sub-Saharan Africa, South/East | | Namibia | | 20428 | | MACRO_DHS | | 2007 | | 6,636 | | 19,522 |  | |
| Sub-Saharan Africa, South/East | | Namibia | | 20417 | | MACRO_DHS | | 2000 | | 4,780 | | 14,946 |  | |
| Sub-Saharan Africa, South/East | | Namibia | | 20404 | | MACRO_DHS | | 1992 | | 3,710 | | 13,372 |  | |
| Asia | | Nepal | | 21240 | | MACRO_DHS | | 2011 | | 8,800 | | 26,615 | X | |
| Asia | | Nepal | | 20462 | | MACRO_DHS | | 2006 | | 7,791 | | 26,394 |  | |
| Asia | | Nepal | | 20450 | | MACRO_DHS | | 2001 | | 7,772 | | 28,955 |  | |
| Asia | | Nepal | | 20437 | | MACRO_DHS | | 1996 | | 7,479 | | 29,156 |  | |
| Latin America and the Caribbean | | Nicaragua | | 126952 | | NIC/DHS_ENDESA | | 2012 | | 11,295 | | 31,244 | X | |
| Latin America and the Caribbean | | Nicaragua | | 20487 | | MACRO_DHS | | 2001 | | 9,275 | | 34,157 |  | |
| Latin America and the Caribbean | | Nicaragua | | 20478 | | MACRO_DHS | | 1998 | | 9,696 | | 36,820 |  | |
| Sub-Saharan Africa, West/Central | | Niger | | 74393 | | MACRO_DHS | | 2012 | | 9,209 | | 44,183 | X | |
| Sub-Saharan Africa, West/Central | | Niger | | 20499 | | MACRO_DHS | | 2006 | | 7,205 | | 34,378 |  | |
| Sub-Saharan Africa, West/Central | | Niger | | 20537 | | MACRO_DHS | | 1998 | | 5,921 | | 28,888 |  | |
| Sub-Saharan Africa, West/Central | | Niger | | 20518 | | MACRO_DHS | | 1992 | | 5,068 | | 23,841 |  | |
| Sub-Saharan Africa, West/Central | | Nigeria | | 77390 | | MACRO_DHS | | 2013 | | 27,451 | | 119,386 | X | |
| Sub-Saharan Africa, West/Central | | Nigeria | | 30991 | | MACRO_MIS | | 2010 | | 4,632 | | 19,644 |  | |
| Sub-Saharan Africa, West/Central | | Nigeria | | 21433 | | MACRO_DHS | | 2008 | | 23,751 | | 104,808 |  | |
| Sub-Saharan Africa, West/Central | | Nigeria | | 20567 | | MACRO_DHS | | 2003 | | 5,111 | | 23,038 |  | |
| Sub-Saharan Africa, West/Central | | Nigeria | | 20552 | | MACRO_DHS | | 1990 | | 6,477 | | 28,123 |  | |
| Asia | | Pakistan | | 77521 | | MACRO_DHS | | 2013 | | 11,965 | | 50,238 | X | |
| Asia | | Pakistan | | 20595 | | MACRO_DHS | | 2007 | | 8,798 | | 39,049 |  | |
| Asia | | Pakistan | | 20584 | | MACRO_DHS | | 1991 | | 5,905 | | 27,369 |  | |
| Latin America and the Caribbean | | Peru | | 209930 | | MACRO_DHS | | 2014 | | 17,488 | | 47,633 | X | |
| Latin America and the Caribbean | | Peru | | 270471 | | MACRO_DHS | | 2012 | | 16,620 | | 47,261 |  | |
| Latin America and the Caribbean | | Peru | | 270470 | | MACRO_DHS | | 2011 | | 15,636 | | 46,194 |  | |
| Latin America and the Caribbean | | Peru | | 270469 | | MACRO_DHS | | 2010 | | 15,886 | | 46,780 |  | |
| Latin America and the Caribbean | | Peru | | 270404 | | MACRO_DHS | | 2009 | | 16,887 | | 50,084 |  | |
| Latin America and the Caribbean | | Peru | | 275090 | | MACRO_DHS | | 2008 | | 28,613 | | 89,220 |  | |
| Latin America and the Caribbean | | Peru | | 20649 | | MACRO_DHS | | 2000 | | 18,931 | | 65,453 |  | |
| Latin America and the Caribbean | | Peru | | 20638 | | MACRO_DHS | | 1996 | | 19,835 | | 72,390 |  | |
| Latin America and the Caribbean | | Peru | | 20626 | | MACRO_DHS | | 1992 | | 10,244 | | 38,783 |  | |
| Asia | | Philippines | | 142943 | | MACRO_DHS | | 2013 | | 10,125 | | 31,680 | X | |
| Asia | | Philippines | | 21421 | | MACRO_DHS | | 2008 | | 8,639 | | 28,518 |  | |
| Asia | | Philippines | | 20699 | | MACRO_DHS | | 2003 | | 8,750 | | 30,443 |  | |
| Asia | | Philippines | | 20683 | | MACRO_DHS | | 1998 | | 8,662 | | 32,626 |  | |
| Asia | | Philippines | | 20674 | | MACRO_DHS | | 1993 | | 9,197 | | 35,863 |  | |
| Sub-Saharan Africa, South/East | | Rwanda | | 157063 | | MACRO_DHS | | 2015 | | 8,736 | | 30,058 | X | |
| Sub-Saharan Africa, South/East | | Rwanda | | 56040 | | MACRO_DHS | | 2011 | | 8,501 | | 32,639 |  | |
| Sub-Saharan Africa, South/East | | Rwanda | | 21222 | | MACRO_DHS | | 2008 | | 4,686 | | 18,421 |  | |
| Sub-Saharan Africa, South/East | | Rwanda | | 20740 | | MACRO_DHS | | 2005 | | 7,045 | | 30,072 |  | |
| Sub-Saharan Africa, South/East | | Rwanda | | 20722 | | MACRO_DHS | | 2000 | | 6,539 | | 27,602 |  | |
| Sub-Saharan Africa, South/East | | Rwanda | | 20711 | | MACRO_DHS | | 1992 | | 4,292 | | 19,440 |  | |
| Sub-Saharan Africa, West/Central | | Sao Tome and Principe | | 26866 | | MACRO_DHS | | 2009 | | 2,014 | | 7,620 | X | |
| Sub-Saharan Africa, West/Central | | Senegal | | 286772 | | MACRO_DHS | | 2016 | | 5,836 | | 22,740 | X | |
| Sub-Saharan Africa, West/Central | | Senegal | | 218592 | | MACRO_DHS | | 2015 | | 5,906 | | 23,250 |  | |
| Sub-Saharan Africa, West/Central | | Senegal | | 191270 | | MACRO_DHS | | 2014 | | 5,733 | | 22,365 |  | |
| Sub-Saharan Africa, West/Central | | Senegal | | 111432 | | MACRO_DHS | | 2013 | | 5,650 | | 22,563 |  | |
| Sub-Saharan Africa, West/Central | | Senegal | | 56063 | | MACRO_DHS | | 2011 | | 10,652 | | 42,510 |  | |
| Sub-Saharan Africa, West/Central | | Senegal | | 11540 | | MACRO_MIS | | 2009 | | 13,134 | | 53,608 |  | |
| Sub-Saharan Africa, West/Central | | Senegal | | 26855 | | MACRO_DHS | | 2005 | | 9,593 | | 39,895 |  | |
| Sub-Saharan Africa, West/Central | | Senegal | | 20780 | | MACRO_DHS | | 1997 | | 6,097 | | 27,448 |  | |
| Sub-Saharan Africa, West/Central | | Senegal | | 20767 | | MACRO_DHS | | 1993 | | 4,534 | | 20,815 |  | |
| Sub-Saharan Africa, West/Central | | Sierra Leone | | 131467 | | MACRO_DHS | | 2013 | | 12,352 | | 47,392 | X | |
| Sub-Saharan Africa, West/Central | | Sierra Leone | | 21258 | | MACRO_DHS | | 2008 | | 5,876 | | 21,136 |  | |
| Sub-Saharan Africa, South/East | | South Africa | | 20796 | | MACRO_DHS | | 1998 | | 8,223 | | 22,934 | X | |
| North Africa / Middle East | | Sudan | | 20813 | | MACRO_DHS | | 1990 | | 5,277 | | 25,805 | X | |
| Sub-Saharan Africa, South/East | | Swaziland | | 20829 | | MACRO_DHS | | 2007 | | 3,488 | | 11,410 | X | |
| Asia | | Tajikistan | | 74460 | | MACRO_DHS | | 2012 | | 6,172 | | 19,938 | X | |
| Sub-Saharan Africa, South/East | | Tanzania | | 218593 | | MACRO_DHS | | 2016 | | 9,721 | | 37,169 | X | |
| Sub-Saharan Africa, South/East | | Tanzania | | 21331 | | MACRO_DHS | | 2010 | | 7,326 | | 29,777 |  | |
| Sub-Saharan Africa, South/East | | Tanzania | | 20875 | | MACRO_DHS | | 2005 | | 7,573 | | 30,557 |  | |
| Sub-Saharan Africa, South/East | | Tanzania | | 20865 | | MACRO_DHS | | 1999 | | 2,935 | | 11,952 |  | |
| Sub-Saharan Africa, South/East | | Tanzania | | 20852 | | MACRO_DHS | | 1996 | | 6,083 | | 24,890 |  | |
| Sub-Saharan Africa, South/East | | Tanzania | | 20841 | | MACRO_DHS | | 1992 | | 6,913 | | 29,143 |  | |
| Sub-Saharan Africa, West/Central | | The Gambia | | 77384 | | MACRO_DHS | | 2013 | | 6,845 | | 26,601 | X | |
| Asia | | Timor-Leste | | 21274 | | MACRO_DHS | | 2010 | | 7,969 | | 35,998 | X | |
| Sub-Saharan Africa, West/Central | | Togo | | 77515 | | MACRO_DHS | | 2014 | | 6,944 | | 26,264 | X | |
| Sub-Saharan Africa, West/Central | | Togo | | 20909 | | MACRO_DHS | | 1998 | | 6,289 | | 26,269 |  | |
| Sub-Saharan Africa, West/Central | | Togo | | 20896 | | MACRO_DHS | | 1988 | | 2,536 | | 10,782 |  | |
| North Africa / Middle East | | Tunisia | | 20926 | | MACRO_DHS | | 1988 | | 3,856 | | 16,463 | X | |
| North Africa / Middle East | | Turkey | | 20954 | | MACRO_DHS | | 2004 | | 7,360 | | 22,443 | X | |
| North Africa / Middle East | | Turkey | | 20947 | | MACRO_DHS | | 1998 | | 5,578 | | 17,791 |  | |
| North Africa / Middle East | | Turkey | | 20936 | | MACRO_DHS | | 1993 | | 5,923 | | 19,762 |  | |
| Sub-Saharan Africa, South/East | | Uganda | | 56021 | | MACRO_DHS | | 2011 | | 6,393 | | 28,609 | X | |
| Sub-Saharan Africa, South/East | | Uganda | | 13109 | | MACRO_MIS | | 2010 | | 3,143 | | 13,863 |  | |
| Sub-Saharan Africa, South/East | | Uganda | | 21014 | | MACRO_DHS | | 2006 | | 6,417 | | 30,090 |  | |
| Sub-Saharan Africa, South/East | | Uganda | | 20993 | | MACRO_DHS | | 2001 | | 5,501 | | 23,410 |  | |
| Sub-Saharan Africa, South/East | | Uganda | | 20976 | | MACRO_DHS | | 1995 | | 5,465 | | 22,752 |  | |
| Sub-Saharan Africa, South/East | | Uganda | | 20964 | | MACRO_DHS | | 1989 | | 3,563 | | 16,074 |  | |
| Asia | | Ukraine | | 21024 | | MACRO_DHS | | 2007 | | 4,811 | | 8,007 | X | |
| Asia | | Uzbekistan | | 21033 | | MACRO_DHS | | 1996 | | 3,018 | | 9,650 | X | |
| Asia | | Vietnam | | 21058 | | MACRO_DHS | | 2002 | | 5,390 | | 14,383 | X | |
| Asia | | Vietnam | | 21049 | | MACRO_DHS | | 1997 | | 5,352 | | 15,517 |  | |
| North Africa / Middle East | | Yemen | | 112500 | | MACRO_DHS | | 2013 | | 14,688 | | 64,602 | X | |
| North Africa / Middle East | | Yemen | | 21068 | | MACRO_DHS | | 1992 | | 5,059 | | 27,081 |  | |
| Sub-Saharan Africa, South/East | | Zambia | | 77516 | | MACRO_DHS | | 2014 | | 12,421 | | 49,207 | X | |
| Sub-Saharan Africa, South/East | | Zambia | | 21117 | | MACRO_DHS | | 2007 | | 5,410 | | 21,366 |  | |
| Sub-Saharan Africa, South/East | | Zambia | | 21102 | | MACRO_DHS | | 2002 | | 5,831 | | 23,805 |  | |
| Sub-Saharan Africa, South/East | | Zambia | | 21090 | | MACRO_DHS | | 1997 | | 5,996 | | 24,799 |  | |
| Sub-Saharan Africa, South/East | | Zambia | | 21079 | | MACRO_DHS | | 1992 | | 5,186 | | 22,122 |  | |
| Sub-Saharan Africa, South/East | Zimbabwe | | 157066 | | MACRO_DHS | | 2015 | | 7,253 | | 20,791 | | | X |
| Sub-Saharan Africa, South/East | | Zimbabwe | | 55992 | | MACRO_DHS | | 2011 | | 6,725 | | 19,279 |  | |
| Sub-Saharan Africa, South/East | | Zimbabwe | | 21163 | | MACRO_DHS | | 2006 | | 6,281 | | 19,489 |  | |
| Sub-Saharan Africa, South/East | | Zimbabwe | | 21151 | | MACRO_DHS | | 1999 | | 4,207 | | 14,184 |  | |
| Sub-Saharan Africa, South/East | | Zimbabwe | | 21139 | | MACRO_DHS | | 1994 | | 4,388 | | 16,777 |  | |
| Sub-Saharan Africa, South/East | | Zimbabwe | | 21126 | | MACRO_DHS | | 1989 | | 3,005 | | 12,405 |  | |
