# Supplementary material for: Development and validation of a new method for indirect estimation of neonatal, infant, and child mortality trends using summary birth histories
Source: PLoS Med. 2018 Oct 31;15(10):e1002687. doi: 10.1371/journal.pmed.1002687 (PMC6209133; doi:10.1371/journal.pmed.1002687)
Supplement: S2 Table — An additional 342 surveys were used for external validation. Of these, 243 were SBH-only surveys; the rest were additional CBH sources used for comparison but not used in training the model. CBH, complete birth history; SBH, summary birth history. (DOCX) [file pmed.1002687.s003.docx]

| **Country** | **GHDx nid** | **Data Type** | **Children** | **Citation** |
| --- | --- | --- | --- | --- |
| Afghanistan | 56099 | cbh | 113,806 | Central Statistics Organization (Afghanistan), ICF Macro, Indian Institute of Health Management Research (IIHMR), Ministry of Public Health (Afghanistan), World Health Organization Regional Office for the Eastern Mediterranean (EMRO-WHO). Afghanistan Special Demographic and Health Survey 2010. Fairfax, United States: ICF International. |
| Afghanistan | 56830 | sbh | 64,660 | Central Statistics Organization (Afghanistan), United Nations Children's Fund (UNICEF). Afghanistan Multiple Indicator Cluster Survey 2010-2011. New York, United States: United Nations Children's Fund (UNICEF), 2013. |
| Albania | 595 | sbh | 8,661 | National Institute of Statistics (Albania), United Nations Children's Fund (UNICEF). Albania Multiple Indicator Cluster Survey 2000. New York, United States: United Nations Children's Fund (UNICEF). |
| Albania | 608 | sbh | 7,531 | National Institute of Statistics (Albania), United Nations Children's Fund (UNICEF). Albania Multiple Indicator Cluster Survey 2005. New York, United States: United Nations Children's Fund (UNICEF). |
| Albania | 56862 | sbh | 29,794 | European Union (EU), Government of Albania, Institute of Statistics (Albania). Albania Census 2011. |
| Algeria | 627 | cbh | 29,406 | National Office of Statistics (Algeria), Ministry of Health, Population and Hospital Reform (Algeria), League of Arab States. Algeria Family Health Survey 2002-2003. |
| Algeria | 210614 | cbh | 58,390 | Ministry of Health and Population (Algeria), United Nations Children's Fund (UNICEF). Algeria Multiple Indicator Cluster Survey 2012-2013. New York, United States: United Nations Children's Fund (UNICEF), 2016. |
| Angola | 687 | sbh | 23,911 | National Institute of Statistics (Angola), United Nations Children's Fund (UNICEF). Angola Multiple Indicator Cluster Survey 2001. New York, United States: United Nations Children's Fund (UNICEF). |
| Angola | 672 | cbh | 2,931 | COSEP-Consulting Ltd., ConsaÃºde Ltd., Macro International, Inc, Ministry of Health (Angola). Angola Malaria Indicator Survey 2006-2007. Fairfax, United States: ICF International. |
| Angola | 30394 | sbh | 41,890 | National Institute of Statistics (Angola), Oxford Policy Management, United Nations Children's Fund (UNICEF). Angola Integrated Inquiry into People's Well-Being 2008-2009. |
| Armenia | 811 | sbh | 143,754 | National Statistical Service of the Republic of Armenia, Minnesota Population Center. Armenia Population and Housing Census 2001 from the Integrated Public Use Microdata Series, International: [Machine-readable database]. Minneapolis: University of Minnesota. |
| Azerbaijan | 881 | sbh | 12,900 | State Statistics Committee of Azerbaijan, United Nations Children's Fund (UNICEF). Azerbaijan Multiple Indicator Cluster Survey 2000. New York, United States: United Nations Children's Fund (UNICEF). |
| Azerbaijan | 18865 | cbh | 13,565 | Macro International, Inc, State Statistical Committee of Azerbaijan. Azerbaijan Demographic and Health Survey 2006. Fairfax, United States: ICF International. |
| Bangladesh | 18920 | cbh | 319,622 | Associates for Community and Population Research (ACPR), International Centre for Diarrhoeal Disease Research, Bangladesh (ICDDR,B), Johns Hopkins University (JHU), Mitra and Associates, National Institute of Population Research and Training (NIPORT), ORC Macro. Bangladesh Special Demographic and Health Survey 2001. Fairfax, United States: ICF International. |
| Bangladesh | 126906 | sbh | 705,724 | Bangladesh Bureau of Statistics. Bangladesh Multiple Indicator Cluster Survey 2009. Dhaka, Bangladesh: Bangladesh Bureau of Statistics. |
| Bangladesh | 151086 | sbh | 112,041 | Bangladesh Bureau of Statistics (BBS), Government of Bangladesh, Ministry of Planning (Bangladesh), United Nations Children's Fund (UNICEF). Bangladesh Multiple Indicator Cluster Survey 2012-2013. New York, United States: United Nations Children's Fund (UNICEF), 2015. |
| Belize | 1089 | sbh | 4,206 | Statistical Institute of Belize, United Nations Children's Fund (UNICEF). Belize Multiple Indicator Cluster Survey 2006. New York, United States: United Nations Children's Fund (UNICEF). |
| Belize | 76699 | sbh | 8,888 | Statistical Institute of Belize, United Nations Children's Fund (UNICEF). Belize Multiple Indicator Cluster Survey 2011. New York, United States: United Nations Children's Fund (UNICEF), 2013. |
| Benin | 206075 | cbh | 45,183 | National Institute of Statistics and Economic Analysis (INSAE) (Benin), United Nations Children's Fund (UNICEF). Benin Multiple Indicator Cluster Survey 2014. New York, United States: United Nations Children's Fund (UNICEF), 2017. |
| Bhutan | 40028 | sbh | 31,697 | National Statistics Bureau (Bhutan), United Nations Children's Fund (UNICEF), United Nations Population Fund (UNFPA). Bhutan Multiple Indicator Cluster Survey 2010. New York, United States: United Nations Children's Fund (UNICEF). |
| Bolivia | 1245 | sbh | 12,777 | National Institute of Statistics (Bolivia), World Bank (WB), Inter-American Development Bank (IDB), United Nations Economic Commission for Latin America and the Caribbean (CEPAL). Bolivia Household Survey 2000. La Paz, Bolivia: National Institute of Statistics (Bolivia). |
| Bolivia | 1289 | sbh | 11,779 | Population Development and Environment (PODEMA), National Directorate of Epidemiology (Bolivia), United Nations Children's Fund (UNICEF). Bolivia Multiple Indicator Cluster Survey 2000. New York, United States: United Nations Children's Fund (UNICEF). |
| Bolivia | 1362 | sbh | 485,628 | National Institute of Statistics (Bolivia), Minnesota Population Center. Bolivia National Census of Population and Housing 2001 from the Integrated Public Use Microdata Series, International: [Machine-readable database]. Minneapolis: University of Minnesota. |
| Bolivia | 19001 | cbh | 45,116 | Macro International, Inc, Ministry of Health and Sports (Bolivia), National Institute of Statistics (Bolivia). Bolivia Demographic and Health Survey 2003-2004. Fairfax, United States: ICF International. |
| Botswana | 1400 | cbh | 18,073 | Central Statistics Office (Botswana). Botswana Family Health Survey 1996. Gaborone, Botswana: Central Statistics Office (Botswana). |
| Botswana | 1404 | sbh | 12,745 | Central Statistics Office (Botswana), United Nations Children's Fund (UNICEF). Botswana Multiple Indicator Cluster Survey 2000. New York, United States: United Nations Children's Fund (UNICEF), 2015. |
| Botswana | 294205 | sbh | 92,210 | Central Statistics Office (Botswana), Minnesota Population Center. Botswana Population and Housing Census 2001 from the Integrated Public Use Microdata Series, International. Minneapolis: University of Minnesota, 2017. |
| Botswana | 21970 | sbh | 22,722 | Central Statistics Office (Botswana). Botswana Demographic Survey 2006. Gaborone, Botswana: Central Statistics Office (Botswana). |
| Botswana | 22125 | cbh | 10,858 | Statistics Botswana, Botswana Familiy Health Survey 2007-2008 |
| Botswana | 294235 | sbh | 95,455 | Central Statistics Office (Botswana), Minnesota Population Center. Botswana Population and Housing Census 2011 from the Integrated Public Use Microdata Series, International. Minneapolis: University of Minnesota, 2017. |
| Brazil | 19035 | cbh | 15,363 | Brazilian Society for Family Welfare (BEMFAM), Macro International, Inc. Brazil Demographic and Health Survey 1991. Fairfax, United States: ICF International. |
| Brazil | 38230 | sbh | 4,830,714 | Brazilian Institute of Geography and Statistics (IBGE), Minnesota Population Center. Brazil General Census 2000 from the Integrated Public Use Microdata Series, International: [Machine-readable database]. Minneapolis: University of Minnesota. |
| Brazil | 1488 | sbh | 177,981 | Brazilian Institute of Geography and Statistics (IBGE). Brazil National Household Sample Survey 2002. Rio de Janeiro, Brazil: Brazilian Institute of Geography and Statistics (IBGE). |
| Brazil | 1489 | sbh | 174,600 | Brazilian Institute of Geography and Statistics (IBGE). Brazil National Household Sample Survey 2003. Rio de Janeiro, Brazil: Brazilian Institute of Geography and Statistics (IBGE). |
| Brazil | 1490 | sbh | 180,887 | Brazilian Institute of Geography and Statistics (IBGE). Brazil National Household Sample Survey 2004. Rio de Janeiro, Brazil: Brazilian Institute of Geography and Statistics (IBGE). |
| Brazil | 80311 | sbh | 182,709 | Brazilian Institute of Geography and Statistics (IBGE). Brazil National Household Sample Survey 2005. Rio de Janeiro, Brazil: Brazilian Institute of Geography and Statistics (IBGE). |
| Brazil | 93528 | sbh | 179,995 | Brazilian Institute of Geography and Statistics (IBGE). Brazil National Household Sample Survey 2006. Rio de Janeiro, Brazil: Brazilian Institute of Geography and Statistics (IBGE). |
| Brazil | 93490 | sbh | 174,714 | Brazilian Institute of Geography and Statistics (IBGE). Brazil National Household Sample Survey 2007. Rio de Janeiro, Brazil: Brazilian Institute of Geography and Statistics (IBGE). |
| Brazil | 93487 | sbh | 166,527 | Brazilian Institute of Geography and Statistics (IBGE). Brazil National Household Sample Survey 2008. Rio de Janeiro, Brazil: Brazilian Institute of Geography and Statistics (IBGE). |
| Brazil | 93522 | sbh | 166,438 | Brazilian Institute of Geography and Statistics (IBGE). Brazil National Household Sample Survey 2009. |
| Brazil | 105322 | sbh | 3,915,750 | Minnesota Population Center, Brazilian Institute of Geography and Statistics. Brazil Demographic Census 2010 from the Integrated Public Use Microdata Series, International: [Machine-readable database]. Minneapolis: University of Minnesota, 2013. |
| Brazil | 106724 | sbh | 145,313 | Brazilian Institute of Geography and Statistics (IBGE). Brazil National Household Sample Survey 2011. Rio de Janeiro, Brazil: Brazilian Institute of Geography and Statistics (IBGE), 2012. |
| Brazil | 156581 | sbh | 144,682 | Brazilian Institute of Geography and Statistics (IBGE). Brazil National Household Sample Survey 2012. Rio de Janeiro, Brazil: Brazilian Institute of Geography and Statistics (IBGE). |
| Brazil | 156583 | sbh | 144,033 | Brazilian Institute of Geography and Statistics (IBGE). Brazil National Household Sample Survey 2013. Rio de Janeiro, Brazil: Brazilian Institute of Geography and Statistics (IBGE). |
| Brazil | 238441 | sbh | 141,577 | Brazilian Institute of Geography and Statistics (IBGE). Brazil National Household Sample Survey 2014. Rio de Janeiro, Brazil: Brazilian Institute of Geography and Statistics (IBGE). |
| Brazil | 281548 | sbh | 135,941 | Brazilian Institute of Geography and Statistics (IBGE). Brazil National Household Sample Survey 2015. Rio de Janeiro, Brazil: Brazilian Institute of Geography and Statistics (IBGE). |
| Burkina Faso | 1927 | sbh | 25,541 | National Institute of Statistics and Demography (Burkina Faso), United Nations Children's Fund (UNICEF). Burkina Faso Multiple Indicator Cluster Survey 2006. New York, United States: United Nations Children's Fund (UNICEF). |
| Burkina Faso | 105403 | sbh | 946,618 | Minnesota Population Center, National Institute of Statistics and Demography (Burkina Faso). Burkina Faso Population and Housing Census 2006 from the Integrated Public Use Microdata Series, International: [Machine-readable database]. Minneapolis: University of Minnesota, 2013. |
| Burkina Faso | 188785 | sbh | 27,986 | ICF International, National Institute of Statistics and Demography (Burkina Faso), National Program for the Fight Against Malaria (PNLP) (Burkina Faso). Burkina Faso Malaria Indicator Survey 2014. Fairfax, United States: ICF International, 2015. |
| Burundi | 1970 | sbh | 20,848 | Burundi Institute of Statistics and Economic Studies. Burundi Priority Survey 1998-1999. |
| Burundi | 1994 | sbh | 13,864 | Burundi Institute of Statistics and Economic Studies, United Nations Children's Fund (UNICEF). Burundi Multiple Indicator Cluster Survey 2000. New York, United States: United Nations Children's Fund (UNICEF). |
| Burundi | 1981 | sbh | 25,768 | United Nations Children's Fund (UNICEF), Burundi Institute of Statistics and Economic Studies, United Nations Population Fund (UNFPA). Burundi Multiple Indicator Cluster Survey 2005. New York, United States: United Nations Children's Fund (UNICEF). |
| Burundi | 108080 | sbh | 13,647 | Burundi Institute of Statistics and Economic Studies, ICF Macro, Ministry of Public Health and the Fight Against AIDS (Burundi), National Institute of Public Health (Burundi). Burundi Malaria Indicator Survey 2012-2013. Fairfax, United States: ICF International, 2013. |
| Cambodia | 19170 | cbh | 18,969 | Macro International, Inc, Ministry of Health (Cambodia), National Institute of Public Health (Cambodia). Cambodia Special Demographic and Health Survey 1998. Fairfax, United States: ICF International. |
| Cambodia | 35322 | sbh | 708,771 | National Institute of Statistics (Cambodia), Minnesota Population Center. Cambodia General Population Census 1998 from the Integrated Public Use Microdata Series, International: [Machine-readable database]. Minneapolis: University of Minnesota. |
| Cambodia | 2002 | sbh | 58,962 | National Institute of Statistics (Cambodia). Cambodia Intercensal Population Survey 2004. |
| Cambodia | 35329 | sbh | 663,330 | National Institute of Statistics (Cambodia), Minnesota Population Center. Cambodia General Population Census 2008 from the Integrated Public Use Microdata Series, International: [Machine-readable database]. Minneapolis: University of Minnesota, 2011. |
| Cameroon | 2053 | sbh | 14,980 | Department of Statistics and Accounting, Ministery of the Economy and Finance (Cameroon) and United Nations Children's Fund (UNICEF). Cameroon Multiple Indicator Cluster Survey 2000. New York, United States: United Nations Children's Fund (UNICEF). |
| Cameroon | 105800 | sbh | 1,046,493 | Minnesota Population Center, National Institute of Statistics (Cameroon), Central Bureau of the Census and Population Studies (Cameroon). Cameroon Population and Housing Census 2005 from the Integrated Public Use Microdata Series, International: [Machine-readable database]. Minneapolis: University of Minnesota, 2013 |
| Cameroon | 244455 | cbh | 26,201 | Ministry of Public Health (Cameroon), National Institute of Statistics (Cameroon), United Nations Children's Fund (UNICEF). Cameroon Multiple Indicator Cluster Survey 2014. New York, United States: United Nations Children's Fund (UNICEF), 2017. |
| Cape Verde | 27511 | cbh | 16,772 | Cape Verde National Statistics Institute (INE) Division of Reproductive Health-Centers for Disease Control and Prevention (CDC). (2000) Cape Verde Reproductive Health Survey 1998. Atlanta, United States: Centers for Disease Control and Prevention (CDC). |
| Central African Republic | 2209 | sbh | 59,424 | Division of Statistics and Economic Studies (Central African Republic), Ministry of Economy, Planning and International Cooperation (Central African Republic), United Nations Children's Fund (UNICEF). Central African Republic Multiple Indicator Cluster Survey 2000. New York, United States: United Nations Children's Fund (UNICEF). |
| Central African Republic | 2223 | sbh | 35,974 | United Nations Children's Fund (UNICEF). Central African Republic Multiple Indicator Cluster Survey 2006. New York, United States: United Nations Children's Fund (UNICEF). |
| Central African Republic | 82832 | sbh | 37,331 | Central African Institute of Statistics, Economic and Social Studies (ICASEES) (Central African Republic), ICF International. Central African Republic Multiple Indicator Cluster Survey 2010-2011. Fairfax, United States: ICF International, 2013. |
| Chad | 2244 | sbh | 21,938 | United Nations Children's Fund (UNICEF), Census Bureau (Chad), National Institute of Statistical, Economic and Demographic Studies (Chad). Chad Multiple Indicator Cluster Survey 2000. New York, United States: United Nations Children's Fund (UNICEF). |
| Chad | 76701 | sbh | 61,128 | Ministry of Planning, Economy, and International Cooperation (Chad), National Institute of Statistical, Economic and Demographic Studies (Chad), United Nations Children's Fund (UNICEF). Chad Multiple Indicator Cluster Survey 2010. New York, United States: United Nations Children's Fund (UNICEF), 2014. |
| Chile | 2311 | sbh | 623,140 | National Institute of Statistics (INE) (Chile), Minnesota Population Center. Chile General Population and Housing Census 2002 from the Integrated Public Use Microdata Series, International: [Machine-readable database]. Minneapolis: University of Minnesota. |
| China | 294255 | sbh | 4,260,950 | China Statistics Press, Minnesota Population Center. China National Population Census 2000 from the Integrated Public Use Microdata Series, International. Minneapolis: University of Minnesota, 2017. |
| Colombia | 3029 | sbh | 1,771,037 | National Administrative Department of Statistics (DANE) (Colombia), Minnesota Population Center. Colombia General Census 2005-2006 from the Integrated Public Use Microdata Series, International: [Machine-readable database]. Minneapolis: University of Minnesota. |
| Colombia | 21281 | cbh | 55,239 | ICF Macro, Profamilia. Colombia Demographic and Health Survey 2009-2010. Calverton, United States: ICF Macro, 2011. |
| Comoros | 3114 | sbh | 16,560 | United Nations Development Programme (UNDP), United Nations Children's Fund (UNICEF). Comoros Multiple Indicator Cluster Survey 2000. New York, United States: United Nations Children's Fund (UNICEF). |
| Congo | 3133 | sbh | 18,574 | ICF Macro, National Center for Statistics and Economic Studies (Congo, Rep.). Congo AIDS Indicator Survey 2009. Calverton, United States: ICF Macro. |
| Costa Rica | 3243 | sbh | 189,555 | National Institute of Statistics and Censuses (INEC) (Costa Rica), Minnesota Population Center. Costa Rica National Population and Housing Census 2000 from the Integrated Public Use Microdata Series, International: [Machine-readable database]. Minneapolis: University of Minnesota. |
| Costa Rica | 227111 | sbh | 174,898 | Minnesota Population Center, Costa Rica National Institute of Statistics and Census. Costa Rica Census 2011 from the Integrated Public Use Microdata Series, International: [Machine-readable database]. Minneapolis: University of Minnesota, 2015. |
| Cuba | 60935 | sbh | 13,154 | Ministry of Public Health (Cuba), United Nations Children's Fund (UNICEF). Cuba Multiple Indicator Cluster Survey 2010-2011. New York, United States: United Nations Children's Fund (UNICEF). |
| Cuba | 169975 | sbh | 12,553 | Ministry of Public Health (Cuba), National Office of Statistics (Cuba), United Nations Children's Fund (UNICEF). Cuba Multiple Indicator Cluster Survey 2014. New York, United States: United Nations Children's Fund (UNICEF), 2005. |
| Democratic Republic of the Congo | 3161 | sbh | 39,356 | Ministry of Planning and Reconstruction (Congo, DR), United Nations Children's Fund (UNICEF). Congo, DR Multiple Indicator Cluster Survey 2001. New York, United States: United Nations Children's Fund (UNICEF). |
| Democratic Republic of the Congo | 26998 | sbh | 40,648 | National Statistical Institute (Congo, DR), Ministry of Planning (Congo, DR), United Nations Children's Fund (UNICEF). Congo, DR Multiple Indicator Cluster Survey 2010. New York, United States: United Nations Children's Fund (UNICEF). |
| Djibouti | 3392 | cbh | 10,574 | Department of Statistics and Demographic Studies (Djibouti), League of Arab States, Ministry of Health (Djibouti), Pan Arab Project for Family Health (PAPFAM). Djibouti Family Health Survey 2002. |
| Djibouti | 3404 | sbh | 10,479 | Ministry of Economy, Finance, and Planning in charge of Privatization (Djibouti), Ministry of Health (Djibouti), United Nations Children's Fund (UNICEF). Djibouti Multiple Indicator Cluster Survey 2006. New York, United States: United Nations Children's Fund (UNICEF). |
| Dominican Republic | 27069 | sbh | 8,619 | Center for Social and Demographic Studies (CESDEM), United Nations Children's Fund (UNICEF). Dominican Republic Multiple Indicator Cluster Survey 2000. New York, United States: United Nations Children's Fund (UNICEF). |
| Dominican Republic | 151296 | sbh | 415,435 | National Statistics Office (Dominican Republic), Minnesota Population Center. Dominican Republic Census 2002 from the Integrated Public Use Microdata Series, International: [Machine-readable database]. Minneapolis: University of Minnesota. |
| Dominican Republic | 3455 | cbh | 18,903 | National Statistics Office (Dominican Republic), United Nations Children's Fund (UNICEF). Dominican Republic National Multipurpose Household Survey 2006. Santo Domingo, Dominican Republic: National Statistics Office (Dominican Republic). |
| Dominican Republic | 151304 | sbh | 466,537 | National Statistics Office (Dominican Republic), Minnesota Population Center. Dominican Republic Census 2010 from the Integrated Public Use Microdata Series, International: [Machine-readable database]. Minneapolis: University of Minnesota. |
| Dominican Republic | 200697 | cbh | 58,946 | National Statistics Office (Dominican Republic), United Nations Children's Fund (UNICEF). Dominican Republic Multiple Indicator Cluster Survey 2014. New York, United States: United Nations Children's Fund (UNICEF), 2016. |
| Ecuador | 27615 | cbh | 33,765 | Center for Studies of Population and Social Development (CEPAR), Division of Reproductive Health-Centers for Disease Control and Prevention (CDC). (1995) Ecuador Reproductive Health Survey 1994. Atlanta, United States: Centers for Disease Control and Prevention (CDC). |
| Ecuador | 27621 | cbh | 34,046 | Center for Studies of Population and Social Development (CEPAR) (Ecuador), Division of Reproductive Health-Centers for Disease Control and Prevention (CDC). Ecuador Reproductive Health Survey 1999. Atlanta, United States: Centers for Disease Control and Prevention (CDC), 2001. |
| Ecuador | 3549 | sbh | 613,208 | National Institute of Statistics and Censuses (Ecuador), Minnesota Population Center. Ecuador Population and Housing Census 2001 from the Integrated Public Use Microdata Series, International: [Machine-readable database]. Minneapolis: University of Minnesota. |
| Ecuador | 27630 | cbh | 24,696 | Center for Studies of Population and Social Development (CEPAR) (Ecuador) and Division of Reproductive Health-Centers for Disease Control and Prevention (CDC). (2005) Ecuador Reproductive Health Survey 2004. Quito, Ecuador: CEPAR. |
| Ecuador | 105801 | sbh | 673,109 | Minnesota Population Center, National Institute of Statistics and Census (INEC) (Ecuador). Ecuador Population and Housing Census 2010 from the Integrated Public Use Microdata Series, International: [Machine-readable database]. Minneapolis: University of Minnesota, 2013. |
| Egypt | 157026 | sbh | 16,984 | El-Zanaty and Associates, ICF International, Ministry of Health and Population (Egypt), National Population Council (Egypt). Egypt Special Demographic and Health Survey 2015. Fairfax, United States: ICF International, 2015. |
| El Salvador | 27590 | cbh | 32,149 | El Salvador Demographic Association (ADS), Division of Reproductive Health-Centers for Disease Control and Prevention (CDC). El Salvador Reproductive Health Survey 1998. Atlanta, United States: Centers for Disease Control and Prevention (CDC). |
| El Salvador | 27599 | cbh | 24,442 | AsociaciÃ³n DemogrÃ¡fica SalvadoreÃ±a (ADS), Division of Reproductive Health-Centers for Disease Control and Prevention (CDC). (2004) El Salvador Reproductive Health Survey 2002-2003. San Salvador, El Salvador: ADS. |
| El Salvador | 56476 | sbh | 270,154 | Minnesota Population Center, General Administration of Statistics and Censuses (El Salvador), Ministry of Economy (El Salvador). El Salvador Population and Housing Census 2007 from the Integrated Public Use Microdata Series, International: [Machine-readable database]. Minneapolis: University of Minnesota, 2012. |
| El Salvador | 27606 | cbh | 25,228 | AsociaciÃ³n DemogrÃ¡fica SalvadoreÃ±a (ADS), Division of Reproductive Health-Centers for Disease Control and Prevention (CDC). (2009) El Salvador Reproductive Health Survey 2008. San Salvador, El Salvador: ADS. |
| El Salvador | 200636 | cbh | 24,689 | General Administration of Statistics and Censuses (El Salvador), Ministry of Health (El Salvador), United Nations Children's Fund (UNICEF). El Salvador Multiple Indicator Cluster Survey 2014. New York, United States: United Nations Children's Fund (UNICEF), 2017. |
| Equitoria Guinea | 3655 | sbh | 12,919 | Ministry of Planning, Economic Development and Public Investment (Equatorial Guinea), United Nations Children's Fund (UNICEF). Equatorial Guinea Multiple Indicator Cluster Survey 2000. New York, United States: United Nations Children's Fund (UNICEF). |
| Eritrea | 19546 | cbh | 14,268 | Macro International, Inc, National Statistics Office (Eritrea). Eritrea Demographic and Health Survey 1995-1996. Calverton, United States: Macro International, Inc. |
| Ethiopia | 227133 | sbh | 816,117 | Minnesota Population Center, Ethiopia Central Statistical Agency. Ethiopia Population and Housing Census 2007 from the Integrated Public Use Microdata Series, International: [Machine-readable database]. Minneapolis: University of Minnesota, 2015. |
| Fiji | 105854 | sbh | 41,450 | Minnesota Population Center, Bureau of Statistics (Fiji). Fiji Population and Housing Census 2007 from the Integrated Public Use Microdata Series, International: [Machine-readable database]. Minneapolis: University of Minnesota, 2013 |
| Georgia | 3970 | sbh | 13,793 | National Center for Disease Control (Georgia), State Department of Statistics of Georgia, United Nations Children's Fund (UNICEF). Georgia Multiple Indicator Cluster Survey 2005. New York, United States: United Nations Children's Fund (UNICEF). |
| Georgia | 27494 | cbh | 8,284 | Georgia Center for Disease Control (NCDC), Georgian Ministry of Labor Health and Social Affairs (MOLHSA), Division of Reproductive Health, Centers for Disease Control and Prevention (CDC). Georgia Reproductive Health Survey 2005. Atlanta, United States: Centers for Disease Control and Prevention (CDC). |
| Georgia | 95336 | cbh | 7,773 | Division of Reproductive Health, Centers for Disease Control and Prevention (CDC), Georgia Ministry of Labor, Health and Social Affairs, National Center for Disease Control and Public Health (Georgia), National Statistics Office of Georgia (GeoStat). Georgia Reproductive Health Survey 2010-2011. |
| Ghana | 38508 | sbh | 1,134,199 | Ghana Statistical Service, Minnesota Population Center. Ghana Population and Housing Census 2000 from the Integrated Public Use Microdata Series, International: [Machine-readable database]. Minneapolis: University of Minnesota. |
| Ghana | 4679 | sbh | 22,681 | Ghana Statistical Service. Ghana Living Standards Measurement Survey 2005-2006. Accra, Ghana: Ghana Statistical Service. |
| Ghana | 4694 | sbh | 15,689 | Ministry of Health (MOH) (Ghana), Ghana Statistical Service and United Nations Children's Fund (UNICEF). Ghana Multiple Indicator Cluster Survey 2006. New York, United States: United Nations Children's Fund (UNICEF). |
| Ghana | 21173 | cbh | 25,710 | Ghana Health Service, Ghana Statistical Service, Macro International, Inc. Ghana Special Demographic and Health Survey 2007-2008. Calverton, United States: Macro International, Inc, 2010. |
| Ghana | 160576 | cbh | 34,751 | Ghana Statistical Service, Ministry of Health (Ghana), United Nations Children's Fund (UNICEF). Ghana District Multiple Indicator Cluster Survey 2007-2008. |
| Ghana | 151306 | sbh | 1,275,941 | Ghana Statistical Service, Minnesota Population Center. Ghana Census 2010 from the Integrated Public Use Microdata Series, International: [Machine-readable database]. Minneapolis: University of Minnesota. |
| Ghana | 56241 | sbh | 2,016 | Institute of Statistical, Social and Economic Research, University of Ghana, United Nations Children's Fund (UNICEF). Ghana - Accra Multiple Indicator Cluster Survey 2010-2011. New York, United States: United Nations Children's Fund (UNICEF), 2014. |
| Ghana | 63993 | cbh | 31,145 | Centers for Disease Control and Prevention (CDC), Ghana Statistical Service, Government of Japan, ICF Macro, Ministry of Health (Ghana), Navrongo Health Research Centre, USAID, United Nations Children's Fund (UNICEF), United Nations Population Fund (UNFPA). Ghana Multiple Indicator Cluster Survey 2011. New York, United States: United Nations Children's Fund (UNICEF), 2013. |
| Ghana | 286788 | sbh | 13,050 | Ghana Health Service, Ghana Statistical Service, ICF International, National Malaria Control Program (Ghana), National Public Health and Reference Laboratory (NHPRL)(Ghana). Ghana Malaria Indicator Survey 2016. Fairfax, United States: ICF International, 2017. |
| Guatemala | 45718 | sbh | 23,378 | Inter-American Development Bank (IDB), National Statistics Institute (Guatemala), World Bank. Guatemala Living Standards Measurement Survey 2000. Washington DC, United States: World Bank. |
| Guatemala | 27563 | cbh | 28,731 | Guatemala Ministry of Health and Social Assistance, University of Valle, Division of Reproductive Health-Centers for Disease Control and Prevention (CDC). (2003) Guatemala Reproductive Health Survey 2002. Atlanta, United States: Centers for Disease Control and Prevention (CDC). |
| Guatemala | 4779 | cbh | 45,017 | Guatemala Ministry of Health and Social Assistance, University of Valle and Division of Reproductive Health-Centers for Disease Control and Prevention (CDC). Guatemala Reproductive Health Survey 2008-2009. Atlanta, United States: Centers for Disease Control and Prevention (CDC). |
| Guinea-Bissau | 4808 | sbh | 23,844 | Secretary State of Planning, National Institute of Statistics and Census (INEC), United Nations Children's Fund (UNICEF). Guinea-Bissau Multiple Indicator Cluster Survey 2000. New York, United States: United Nations Children's Fund (UNICEF). |
| Guinea-Bissau | 4818 | sbh | 24,016 | United Nations Children's Fund (UNICEF), Government of Guinea-Bissau. Guinea-Bissau Multiple Indicator Cluster Survey 2006. New York, United States: United Nations Children's Fund (UNICEF). |
| Guinea-Bissau | 174049 | cbh | 27,607 | National Statistics Institute (Guinea-Bissau), United Nations Children's Fund (UNICEF). Guinea-Bissau Multiple Cluster Indicator Survey 2014. New York, United States: United Nations Children's Fund (UNICEF), 2016. |
| Guyana | 4905 | cbh | 2,887 | Bureau of Statistics (Guyana), World Bank. Guyana Living Standards Measurement Survey 1992-1993. |
| Guyana | 4916 | sbh | 11,691 | Bureau of Statistics (Guyana), United Nations Children's Fund (UNICEF). Guyana Multiple Indicator Cluster Survey 2000. New York, United States: United Nations Children's Fund (UNICEF) |
| Guyana | 4837 | cbh | 4,923 | Central Bureau of Statistics (Ghana), Guyana Responsible Parenthood Association (GRPA), Ministry of Health (Guyana), ORC Macro, Pan American Health Organization (PAHO). Guyana AIDS Indicator Survey 2005. Calverton, United States: ORC Macro. |
| Guyana | 4926 | sbh | 12,157 | United Nations Children's Fund (UNICEF), Bureau of Statistics (Guyana). Guyana Multiple Indicator Cluster Survey 2006. New York, United States: United Nations Children's Fund (UNICEF). |
| Guyana | 200598 | cbh | 11,161 | Bureau of Statistics (Guyana), Ministry of Health (Guyana), United Nations Children's Fund (UNICEF). Guyana Multiple Indicator Cluster Survey 2014. New York, United States: United Nations Children's Fund (UNICEF), 2016. |
| Haiti | 106473 | sbh | 439,200 | Minnesota Population Center, Haitian Institute of Statistics and Informatics. Haiti Population and Housing Census 2003 from the Integrated Public Use Microdata Series, International: [Machine-readable database]. Minneapolis: University of Minnesota, 2013 |
| Haiti | 26680 | cbh | 4,711 | Global Fund to Fight Aids Tuberculosis and Malaria (GFATM). Haiti Global Fund Household Survey 2008. |
| Honduras | 27551 | cbh | 23,535 | Honduras Family Planning Association (ASHONPLAFA), Ministry of Health (Honduras), and Division of Reproductive Health-Centers for Disease Control and Prevention (CDC). Honduras Reproductive Health Survey 2001. Tegucigalpa, Honduras: Honduras Family Planning Association (ASHONPLAFA). |
| Honduras | 5009 | sbh | 21,774 | National Institute of Statistics (Honduras). Honduras Survey of Living Conditions 2004. Tegucigalpa, Honduras: National Institute of Statistics (Honduras). |
| India | 23183 | sbh | 757,856 | International Institute for Population Sciences (India). India District Level Household Survey 1998-1999. Mumbai, India: International Institute for Population Sciences (India). |
| India | 23219 | sbh | 1,391,228 | International Institute for Population Sciences (India). India District Level Household Survey 2002-2005. Mumbai, India: International Institute for Population Sciences (India). |
| India | 23258 | sbh | 1,818,042 | International Institute for Population Sciences (India). India District Level Household Survey 2007-2008. Mumbai, India: International Institute for Population Sciences (India), 2010. |
| India | 165390 | sbh | 259,032 | International Institute for Population Sciences (India). India District Level Household Survey 2012-2014. New Delhi, India: Ministry of Health and Family Welfare (India). |
| Indonesia | 6535 | cbh | 568,692 | Central Bureau of Statistics (Indonesia). Indonesia Intercensal Population Survey 1995. |
| Indonesia | 6767 | sbh | 466,726 | Central Bureau of Statistics (Indonesia), Ministry of Health (Indonesia), World Bank. Indonesia National Socioeconomic Survey 1998. |
| Indonesia | 6790 | sbh | 438,949 | Central Bureau of Statistics (Indonesia), Ministry of Health (Indonesia), World Bank. Indonesia National Socioeconomic Survey 1999. |
| Indonesia | 6811 | sbh | 384,103 | Central Bureau of Statistics (Indonesia), Ministry of Health (Indonesia), World Bank. Indonesia National Socioeconomic Survey 2000. |
| Indonesia | 56554 | sbh | 9,961,117 | Minnesota Population Center, Statistics Indonesia. Indonesia Population Census 2000 from the Integrated Public Use Microdata Series, International: [Machine-readable database]. Minneapolis: University of Minnesota, 2012. |
| Indonesia | 6842 | sbh | 458,846 | Statistics Indonesia, Indonesia National Socioeconomic Survey 2001 |
| Indonesia | 43510 | sbh | 432,996 | Statistics Indonesia. Indonesia National Socioeconomic Survey 2002. |
| Indonesia | 6874 | sbh | 449,842 | Statistics Indonesia. Indonesia National Socioeconomic Survey 2003. |
| Indonesia | 6904 | sbh | 501,844 | Statistics Indonesia. Indonesia National Socioeconomic Survey 2004. |
| Indonesia | 5376 | sbh | 510,692 | Statistics Indonesia. Indonesia National Socioeconomic Survey 2005. |
| Indonesia | 5401 | sbh | 545,163 | Statistics Indonesia. Indonesia National Socioeconomic Survey 2006. |
| Indonesia | 6970 | sbh | 582,333 | Statistics Indonesia. Indonesia National Socioeconomic Survey 2007. |
| Indonesia | 43526 | sbh | 131,645 | Statistics Indonesia. Indonesia National Socioeconomic Survey 2008. |
| Indonesia | 43552 | sbh | 129,963 | Statistics Indonesia. Indonesia National Socioeconomic Survey 2009. |
| Indonesia | 30235 | sbh | 125,345 | Statistics Indonesia. Indonesia National Socioeconomic Survey 2010. |
| Indonesia | 56558 | sbh | 10,489,232 | Minnesota Population Center, Statistics Indonesia. Indonesia Population Census 2010 from the Integrated Public Use Microdata Series, International: [Machine-readable database]. Minneapolis: University of Minnesota, 2012. |
| Indonesia | 91740 | sbh | 10,485,859 | Statistics Indonesia. Indonesia Population Census 2010. |
| Indonesia | 85265 | sbh | 130,728 | Statistics Indonesia. Indonesia National Socioeconomic Survey 2011. |
| Indonesia | 150884 | sbh | 534,256 | Central Bureau of Statistics (Indonesia). Indonesia National Socioeconomic Survey 2012. Jakarta, Indonesia: Central Bureau of Statistics (Indonesia). |
| Indonesia | 151184 | sbh | 518,178 | Statistics Indonesia. Indonesia National Socioeconomic Survey 2013. Jakarta, Indonesia: Statistics Indonesia. |
| Iran | 39396 | sbh | 628,318 | Statistical Centre of Iran, Minnesota Population Center. Iran General Census of Population and Housing 2006 from the Integrated Public Use Microdata Series, International: Version 6.1 [Machine-readable database]. Minneapolis: University of Minnesota, 2011. |
| Iraq | 7028 | cbh | 62,359 | United Nations Children's Fund (UNICEF), Central Organization for Statistics and Information Technology (Iraq), Kurdistan Regional Statistics Office. Iraq Multiple Indicator Cluster Survey 2006. New York, United States: United Nations Children's Fund (UNICEF). |
| Iraq | 23429 | sbh | 34,284 | Ministry of Health (Iraq), Central Organization for Statistics and Information Technology (Iraq), Kurdistan Regional Statistics Office, World Health Organization (WHO), Ministry of Health (Kurdistan). Iraq Family Health Survey 2006-2007. |
| Iraq | 76707 | cbh | 136,878 | Central Organization for Statistics and Information Technology (Iraq), Kurdistan Regional Statistics Office, Ministry of Health (Iraq), United Nations Children's Fund (UNICEF). Iraq Multiple Indicator Cluster Survey 2011. New York, United States: United Nations Children's Fund (UNICEF), 2013. |
| Jamaica | 7140 | sbh | 8,532 | United Nations Children's Fund (UNICEF). Jamaica Multiple Indicator Cluster Survey 2000. New York, United States: United Nations Children's Fund (UNICEF). |
| Jamaica | 39450 | sbh | 93,521 | Statistical Institute of Jamaica (STATIN), Minnesota Population Center. Jamaica Population Census 2001 from the Integrated Public Use Microdata Series, International: [Machine-readable database]. Minneapolis: University of Minnesota, 2011. |
| Jamaica | 7149 | sbh | 7,206 | Statistical Institute of Jamaica (STATIN) and United Nations Children's Fund (UNICEF). Jamaica Multiple Indicator Cluster Survey 2005. New York, United States: United Nations Children's Fund (UNICEF). |
| Kazakhstan | 7340 | sbh | 23,329 | Agency of the Republic of Kazakhstan on Statistics and United Nations Children's Fund (UNICEF). Kazakhstan Multiple Indicator Cluster Survey 2006. New York, United States: United Nations Children's Fund (UNICEF). |
| Kazakhstan | 76702 | sbh | 22,048 | Agency of the Republic of Kazakhstan on Statistics, United Nations Children's Fund (UNICEF). Kazakhstan Multiple Indicator Cluster Survey 2010-2011. New York, United States: United Nations Children's Fund (UNICEF), 2013. |
| Kazakhstan | 260403 | sbh | 21,482 | Ministry of National Economy (Kazakhstan), United Nations Children's Fund (UNICEF). Kazakhstan Multiple Indicator Cluster Survey 2015. New York, United States: United Nations Children's Fund (UNICEF), 2017. |
| Kenya | 39481 | sbh | 964,933 | Central Bureau of Statistics (CBS) (Kenya), Minnesota Population Center. Kenya Population and Housing Census 1999 from the Integrated Public Use Microdata Series, International: [Machine-readable database]. Minneapolis: University of Minnesota. |
| Kenya | 153943 | sbh | 976,094 | Central Bureau of Statistics (Kenya), UK Department for International Development (DFID), United Nations Development Programme (UNDP), United Nations Population Fund (UNFPA), United States Agency for International Development (USAID). Kenya Population and Housing Census 1999. |
| Kenya | 7387 | sbh | 31,278 | Central Bureau of Statistics (Kenya), United Nations Children's Fund (UNICEF). Kenya Multiple Indicator Cluster Survey 2000. New York, United States: United Nations Children's Fund (UNICEF). |
| Kenya | 7375 | sbh | 37,583 | Central Bureau of Statistics (Kenya), UK Department for International Development (DFID), United States Agency for International Development (USAID), European Union (EU), Danish International Development Agency (DANIDA), World Bank (WB), United Nations Development Programme (UNDP). Kenya Integrated Household Budget Survey 2005-2006. Nairobi, Kenya: Central Bureau of Statistics (Kenya). |
| Kenya | 57990 | sbh | 14,769 | Centers for Disease Control and Prevention (CDC), KEMRI Wellcome Trust Research Programme (KWTRP), Kenya National Bureau of Statistics, Ministry of Public Health and Sanitation (Kenya), National Coordinating Agency for Population and Development (Kenya), Population Services International (PSI). Kenya Malaria Indicator Survey 2007. |
| Kenya | 133219 | sbh | 26,155 | Centers for Disease Control and Prevention (CDC), Kenya Medical Research Institute (KEMRI), Kenya National Bureau of Statistics, Ministry of Public Health and Sanitation (Kenya), National AIDS Control Council (Kenya), National AIDS and STI Control Program (Kenya), National Coordinating Agency for Population and Development (Kenya), National Public Health Laboratory Services, Ministry of Public Health and Sanitation (Kenya), USAID. Kenya AIDS Indicator Survey 2007. Nairobi, Kenya: Kenya National Bureau of Statistics. |
| Kenya | 7427 | sbh | 2,390,112 | Kenya National Bureau of Statistics, USAID, United Nations Population Fund (UNFPA), United States Census Bureau. Kenya Population and Housing Census 2009. |
| Kenya | 106512 | sbh | 2,388,668 | Minnesota Population Center, Kenya National Bureau of Statistics. Kenya Population Census 2009 from the Integrated Public Use Microdata Series, International: [Machine-readable database]. Minneapolis: University of Minnesota, 2013. |
| Kenya | 218579 | sbh | 14,087 | ICF International, Kenya National Bureau of Statistics, National Malaria Control Program (NMCP) (Kenya). Kenya Malaria Indicator Survey 2015. Fairfax, United States: ICF International, 2015. |
| Kyrgyzstan | 39466 | sbh | 256,968 | National Statistical Committee of the Kyrgyz Republic, Minnesota Population Center. Kyrgyzstan National Population Census 1999 from the Integrated Public Use Microdata Series, International: [Machine-readable database]. Minneapolis: University of Minnesota. |
| Kyrgyzstan | 7540 | sbh | 12,820 | United Nations Children's Fund (UNICEF), National Statistical Committee of the Kyrgyz Republic. Kyrgyzstan Multiple Indicator Cluster Survey 2005-2006. New York, United States: United Nations Children's Fund (UNICEF). |
| Kyrgyzstan | 106520 | sbh | 280,046 | Minnesota Population Center, National Statistical Committee of the Kyrgyz Republic. Kyrgyzstan Population and Housing Census 2009 from the Integrated Public Use Microdata Series, International: [Machine-readable database]. Minneapolis: University of Minnesota, 2013. |
| Kyrgyzstan | 162283 | cbh | 14,527 | National Statistical Committee of the Kyrgyz Republic, United Nations Children's Fund (UNICEF). Kyrgyzstan Multiple Indicator Cluster Survey 2014. New York, United States: United Nations Children's Fund (UNICEF), 2015. |
| Laos | 103973 | cbh | 56,802 | Ministry of Education and Sports (Laos), Ministry of Health (Laos), Ministry of Planning and Investment (Laos). Laos Multiple Indicator Cluster Survey 2011-2012. New York, United States: United Nations Children's Fund (UNICEF), 2013. |
| Lebanon | 44861 | cbh | 9,867 | Central Administration of Statistics (Lebanon), League of Arab States, Ministry of Social Affairs (Lebanon), Pan Arab Project for Family Health (PAPFAM). Lebanon Family Health Survey 2004. |
| Lesotho | 7721 | sbh | 15,482 | Bureau of Statistics (Lesotho), United Nations Children's Fund (UNICEF). Lesotho Multiple Indicator Cluster Survey 2000. New York, United States: United Nations Children's Fund (UNICEF). |
| Liberia | 151310 | sbh | 209,401 | Liberia Institute for Statistics and Geo-information Services (LISGIS), Minnesota Population Center. Liberia Census 2008 from the Integrated Public Use Microdata Series, International: [Machine-readable database]. Minneapolis: University of Minnesota. |
| Liberia | 56828 | sbh | 13,917 | ICF International, Liberia Institute for Statistics and Geo-information Services (LISGIS), National Malaria Control Program (Liberia). Liberia Malaria Indicator Survey 2011. Fairfax, United States: ICF International, 2012. |
| Liberia | 286768 | sbh | 13,869 | ICF International, Liberia Institute for Statistics and Geo-information Services (LISGIS), National Malaria Control Program (Liberia). Liberia Malaria Indicator Survey 2016. Fairfax, United States: ICF International, 2017. |
| Libya | 107340 | cbh | 49,554 | League of Arab States, National Center for Disease Control (Libya), Pan Arab Project for Family Health (PAPFAM). Libya Family Health Survey 2007. |
| Madagascar | 27020 | sbh | 21,615 | National Institute of Statistics (Madagascar), United Nations Children's Fund (UNICEF). Madagascar Multiple Indicator Cluster Survey 2000. New York, United States: United Nations Children's Fund (UNICEF). |
| Madagascar | 69806 | sbh | 23,462 | ICF International, National Institute of Statistics (Madagascar), National Program for the Fight Against Malaria (PNLP) (Madagascar), Pasteur Institute of Madagascar (IPM). Madagascar Malaria Indicator Survey 2011. Fairfax, United States: ICF International. |
| Madagascar | 125594 | cbh | 9,956 | National Institute of Statistics (Madagascar), United Nations Children's Fund (UNICEF). Madagascar - South Multiple Indicator Cluster Survey 2012. New York, United States: United Nations Children's Fund (UNICEF), 2015. |
| Madagascar | 111438 | sbh | 22,075 | ICF International, National Institute of Statistics (Madagascar), National Program for the Fight Against Malaria (PNLP) (Madagascar), Pasteur Institute of Madagascar (IPM). Madagascar Malaria Indicator Survey 2013. Fairfax, United States: ICF International, 2013. |
| Madagascar | 218580 | sbh | 27,819 | ICF International, Ministry of Public Health (Madagascar), National Institute of Statistics (Madagascar), National Program for the Fight Against Malaria (PNLP) (Madagascar), Pasteur Institute of Madagascar (IPM). Madagascar Malaria Indicator Survey 2016. Fairfax, United States: ICF International, 2017. |
| Malawi | 40179 | sbh | 729,974 | National Statistical Office (Malawi), Minnesota Population Center. Malawi Population and Housing Census 1998 from the Integrated Public Use Microdata Series, International: [Machine-readable database]. Minneapolis: University of Minnesota, 2011. |
| Malawi | 7919 | cbh | 78,960 | United Nations Children's Fund (UNICEF), National Statistics Office (Malawi). Malawi Multiple Indicator Cluster Survey 2006. New York, United States: United Nations Children's Fund (UNICEF). |
| Malawi | 40186 | sbh | 875,423 | National Statistical Office (Malawi), Minnesota Population Center. Malawi Population and Housing Census 2008 from the Integrated Public Use Microdata Series, International: [Machine-readable database]. Minneapolis: University of Minnesota, 2011. |
| Malawi | 77387 | sbh | 8,026 | ICF International, National Malaria Control Program (Malawi). Malawi Malaria Indicator Survey 2012. Fairfax, United States: ICF International, 2013. |
| Malawi | 157059 | sbh | 8,126 | ICF International, Ministry of Health (Malawi), National Malaria Control Program (Malawi), National Statistical Office of Malawi. Malawi Malaria Indicator Survey 2014. Fairfax, United States: ICF International, 2015. |
| Malawi | 161662 | cbh | 72,579 | National Statistical Office of Malawi, United Nations Children's Fund (UNICEF). Malawi Multiple Indicator Cluster Survey 2013-2014. New York, United States: United Nations Children's Fund (UNICEF), 2015. |
| Mali | 40235 | sbh | 663,345 | Central Census Bureau (Mali), Minnesota Population Center. Mali General Population and Housing Census 1998 from the Integrated Public Use Microdata Series, International: [Machine-readable database]. Minneapolis: University of Minnesota. |
| Mali | 151311 | sbh | 840,735 | Central Census Bureau (Mali), Minnesota Population Center. Mali Census 2009 from the Integrated Public Use Microdata Series, International: [Machine-readable database]. Minneapolis: University of Minnesota. |
| Mali | 218587 | sbh | 28,960 | ICF International, INFO-STAT (Mali), Ministry of Health and Public Hygiene (Mali), National Institute of Public Health Research (INRSP) (Mali), National Institute of Statistics (INSTAT) (Mali). Mali Malaria Indicator Survey 2015. Fairfax, United States: ICF International, 2016. |
| Mali | 248224 | cbh | 55,820 | Ministry of Health (Mali), Ministry of Planning (Mali), National Institute of Statistics (INSTAT) (Mali), United Nations Children's Fund (UNICEF). Mali Multiple Indicator Cluster Survey 2015. New York, United States: United Nations Children's Fund (UNICEF), 2017. |
| Mauritania | 26871 | cbh | 13,246 | Macro International, Inc, Ministry of Health and Social Affairs (Mauritania), National Office of Statistics (Mauritania). Mauritania Special Demographic and Health Survey 2003-2004. Calverton, United States: Macro International, Inc. |
| Mauritania | 8115 | sbh | 35,683 | National Office of Statistics (Mauritania), United Nations Children's Fund (UNICEF). Mauritania Multiple Indicator Cluster Survey 2007. New York, United States: United Nations Children's Fund (UNICEF). |
| Mauritania | 152783 | cbh | 33,648 | National Office of Statistics (Mauritania), United Nations Children's Fund (UNICEF). Mauritania Multiple Indicator Cluster Survey 2011. New York, United States: United Nations Children's Fund (UNICEF), 2015. |
| Mexico | 43771 | sbh | 5,645,050 | National Institute of Statistics, Geography, and Informatics (Mexico), Minnesota Population Center. Mexico Population and Housing Census 2000 from the Integrated Public Use Microdata Series, International: [Machine-readable database]. Minneapolis: University of Minnesota. |
| Mexico | 43776 | sbh | 5,076,280 | National Institute of Statistics, Geography, and Informatics (Mexico), Minnesota Population Center. Mexico Population and Housing Census 2005 from the Integrated Public Use Microdata Series, International: [Machine-readable database]. Minneapolis: University of Minnesota. |
| Mexico | 23982 | cbh | 78,420 | National Institute of Statistics, Geography, and Informatics (Mexico), National Institute for Public Health (Mexico), National Population Council (Mexico). Mexico National Survey of Demographic Dynamics 2006. Aguascalientes, Mexico: National Institute of Statistics, Geography, and Informatics (Mexico). |
| Mexico | 24006 | sbh | 158,141 | National Institute of Statistics and Geography (INEGI) (Mexico). Mexico National Survey of Demographic Dynamics 2009. |
| Mexico | 56480 | sbh | 6,073,966 | Minnesota Population Center, National Institute of Statistics and Geography (Mexico). Mexico Population and Housing Census 2010 from the Integrated Public Use Microdata Series, International: [Machine-readable database]. Minneapolis: University of Minnesota, 2012. |
| Mexico | 93321 | sbh | 50,526 | National Institute of Statistics and Geography (INEGI) (Mexico). Mexico Household Income and Expenditure Survey 2010. Mexico City, Mexico: National Institute of Statistics and Geography (INEGI) (Mexico). |
| Mexico | 240525 | sbh | 10,418,861 | National Institute of Statistics and Geography (INEGI) (Mexico). Mexico Intercensal Survey 2015. Mexico City, Mexico: National Institute of Statistics and Geography (INEGI) (Mexico). |
| Mexico | 294574 | sbh | 5,403,100 | National Institute of Statistics, Geography and Informatics (INEGI), Minnesota Population Center.&nbsp;Mexico Intercensal Survey 2015&nbsp;from the Integrated Public Use Microdata Series, International. Minneapolis: University of Minnesota, 2017. |
| Moldova | 20339 | cbh | 9,903 | Macro International, Inc, National Scientific and Applied Center for Preventive Medicine (Moldova). Moldova Demographic and Health Survey 2005. Calverton, United States: Macro International, Inc. |
| Mongolia | 43016 | cbh | 17,782 | Ministry of Health and Social Welfare (Mongolia), National Statistical Office of Mongolia, United Nations Statistics Division (UNSD). Mongolia Reproductive Health Survey 1998. |
| Mongolia | 8788 | sbh | 17,766 | National Statistical Office of Mongolia. Mongolia Multiple Indicator Cluster Survey 2000. New York, United States: United Nations Children's Fund (UNICEF). |
| Mongolia | 8777 | sbh | 15,378 | National Statistical Office of Mongolia, United Nations Children's Fund (UNICEF). Mongolia Multiple Indicator Cluster Survey 2005. New York, United States: United Nations Children's Fund (UNICEF). |
| Mongolia | 76704 | sbh | 18,061 | National Statistical Office of Mongolia, United Nations Children's Fund (UNICEF). Mongolia Multiple Indicator Cluster Survey 2010. New York, United States: United Nations Children's Fund (UNICEF), 2013. |
| Morocco | 20351 | cbh | 10,496 | Macro International, Inc, Ministry of Public Health (Morocco). Morocco Special Demographic and Health Survey 1995. Calverton, United States: Macro International, Inc. |
| Morocco | 56492 | sbh | 726,578 | Minnesota Population Center, High Commission for Planning (Morocco). Morocco Population and Housing Census 2004 from the Integrated Public Use Microdata Series, International: [Machine-readable database]. Minneapolis: University of Minnesota, 2012. |
| Morocco | 126909 | sbh | 30,414 | Ministry of Health (Morocco), Pan Arab Project for Family Health (PAPFAM), United Nations Children's Fund (UNICEF), United Nations Population Fund (UNFPA), World Health Organization (WHO). Morocco National Survey on Population and Family Health 2010-2011. |
| Mozambique | 227143 | sbh | 1,359,295 | Minnesota Population Center, Mozambique National Statistics Institute. Mozambique Census 2007 from the Integrated Public Use Microdata Series, International: [Machine-readable database]. Minneapolis: University of Minnesota, 2015. |
| Mozambique | 8906 | sbh | 29,263 | ICF Macro, Ministry of Health (Mozambique), National Institute of Statistics (INE) (Mozambique). Mozambique AIDS Indicator Survey 2009. Calverton, United States: ICF Macro, 2010. |
| Mozambique | 27031 | cbh | 42,215 | United Nations Children's Fund (UNICEF), National Statistics Institute (Mozambique). Mozambique Multiple Indicator Cluster Survey 2008-2009. New York, United States: United Nations Children's Fund (UNICEF). |
| Myanmar | 90696 | cbh | 60,796 | Ministry of Health (Myanmar), Ministry of National Planning and Economic Development (Myanmar), United Nations Children's Fund (UNICEF). Myanmar Multiple Indicator Cluster Survey 2009-2010. |
| Nepal | 162317 | cbh | 28,647 | Central Bureau of Statistics (Nepal), United Nations Children's Fund (UNICEF). Nepal Multiple Indicator Cluster Survey 2014. New York, United States: United Nations Children's Fund (UNICEF), 2015. |
| Nicaragua | 56520 | sbh | 295,576 | Minnesota Population Center, National Institute of Statistics and Censuses (Nicaragua). Nicaragua Population and Housing Census 2005 from the Integrated Public Use Microdata Series, International: [Machine-readable database]. Minneapolis: University of Minnesota, 2012. |
| Nicaragua | 9270 | cbh | 34,055 | Division of Reproductive Health, Centers for Disease Control and Prevention (CDC), National Institute for Development Information (Nicaragua). Nicaragua Reproductive Health Survey 2006-2007. Managua, Nicaragua: National Institute for Development Information (Nicaragua). |
| Niger | 9439 | sbh | 21,570 | Government of Niger, Macro International, Inc, United Nations Children's Fund (UNICEF). Niger Multiple Indicator Cluster Survey 2000. New York, United States: United Nations Children's Fund (UNICEF). |
| Nigeria | 9506 | sbh | 55,461 | National Bureau of Statistics (Nigeria), United Nations Children's Fund (UNICEF). Nigeria Multiple Indicator Cluster Survey 1999. Abuja, Nigeria: National Bureau of Statistics (Nigeria). |
| Nigeria | 25006 | sbh | 52,723 | Federal Office of Statistics (Nigeria). Nigeria Living Standards Survey 2003-2004. |
| Nigeria | 9516 | sbh | 68,689 | United Nations Children's Fund (UNICEF), National Bureau of Statistics (Nigeria). Nigeria Multiple Indicator Cluster Survey 2007. New York, United States: United Nations Children's Fund (UNICEF). |
| Nigeria | 24915 | sbh | 55,508 | Central Bank of Nigeria, National Bureau of Statistics (Nigeria), Nigerian Communications Commission (NCC). Nigeria General Household Survey 2008. |
| Nigeria | 76703 | sbh | 100,531 | National Bureau of Statistics (Nigeria), United Nations Children's Fund (UNICEF). Nigeria Multiple Indicator Cluster Survey 2011. New York, United States: United Nations Children's Fund (UNICEF), 2013. |
| Nigeria | 218590 | sbh | 25,450 | ICF International, National Bureau of Statistics (Nigeria), National Malaria Control Programme (Nigeria), National Population Commission of Nigeria. Nigeria Malaria Indicator Survey 2015. Fairfax, United States: ICF International, 2016. |
| Nigeria | 218613 | cbh | 101,649 | National Agency for the Control of AIDS (Nigeria), National Bureau of Statistics (Nigeria), National Primary Health Care Development Agency (NPHCDA) (Nigeria), United Nations Children's Fund (UNICEF). Nigeria Multiple Indicator Cluster Survey with National Immunization Coverage Survey Supplement 2016-2017. New York, United States: United Nations Children's Fund (UNICEF), 2018. |
| Pakistan | 9919 | cbh | 24,151 | Federal Bureau of Statistics (Pakistan) and World Bank. Pakistan Living Standards Measurement Survey 1991. Islamabad, Pakistan: Federal Bureau of Statistics (Pakistan). |
| Pakistan | 9658 | sbh | 68,941 | Federal Bureau of Statistics (Pakistan). Pakistan Integrated Household Survey 1998-1999. Islamabad, Pakistan: Federal Bureau of Statistics (Pakistan). |
| Pakistan | 9720 | sbh | 68,782 | Federal Bureau of Statistics (Pakistan). Pakistan Integrated Household Survey 2001-2002. Islamabad, Pakistan: Federal Bureau of Statistics (Pakistan). |
| Pakistan | 24818 | sbh | 60,656 | Federal Bureau of Statistics (Pakistan). Pakistan Social and Living Standards Measurement Survey 2005-2006. Islamabad, Pakistan: Federal Bureau of Statistics (Pakistan). |
| Pakistan | 30634 | sbh | 56,277 | Federal Bureau of Statistics (Pakistan). Pakistan Social and Living Standards Measurement Survey 2007-2008. Islamabad, Pakistan: Federal Bureau of Statistics (Pakistan). |
| Palestine | 10001 | cbh | 26,074 | Ministry of Health (Palestine), Palestinian Central Bureau of Statistics, United Nations Children's Fund (UNICEF), United Nations Population Fund (UNFPA). Palestine - West Bank and Gaza Strip Multiple Indicator Cluster Survey 2000. Ramallah, Palestine: Palestinian Central Bureau of Statistics. |
| Palestine | 20596 | cbh | 22,478 | Palestinian Central Bureau of Statistics. Palestine Demographic and Health Survey 2004. |
| Palestine | 9999 | cbh | 51,635 | League of Arab States, Palestinian Central Bureau of Statistics, United Nations Children's Fund (UNICEF). Palestine Family Health Survey 2006-2007. |
| Palestine | 125591 | cbh | 55,823 | Ministry of Health (Palestine), Palestinian Central Bureau of Statistics, United Nations Children's Fund (UNICEF), United Nations Population Fund (UNFPA). Palestine Multiple Indicator Cluster Survey 2010. New York, United States: United Nations Children's Fund (UNICEF), 2014. |
| Palestine | 161590 | cbh | 31,817 | Ministry of Health (Palestine), Palestinian Central Bureau of Statistics, United Nations Children's Fund (UNICEF). Palestine Multiple Indicator Cluster Survey 2014. New York, United States: United Nations Children's Fund (UNICEF), 2015. |
| Panama | 40907 | sbh | 142,057 | Department of Statistics and Census (Panama), Minnesota Population Center. Panama Population and Housing Census 2000 from the Integrated Public Use Microdata Series, International: [Machine-readable database]. Minneapolis: University of Minnesota. |
| Panama | 10224 | sbh | 13,639 | Census and Statistics Directorate (Panama), Ministry of Economy and Finance (Panama), World Bank. Panama Living Standard Measurement Survey 2003. Washington DC, United States: World Bank. |
| Panama | 106529 | sbh | 156,544 | Minnesota Population Center, National Institute of Statistics and Census (Panama). Panama Population and Housing Census 2010 from the Integrated Public Use Microdata Series, International: [Machine-readable database]. Minneapolis: University of Minnesota, 2013. |
| Papua New Guinea | 58191 | sbh | 3,250,144 | National Statistical Office (Papua New Guinea). Papua New Guinea Census 2000. |
| Paraguay | 10326 | cbh | 8,262 | Division of Reproductive Health-Centers for Disease Control and Prevention (CDC). Paraguay Contraceptive Prevalence Survey 1998. Atlanta, United States: Centers for Disease Control and Prevention (CDC). |
| Paraguay | 10357 | sbh | 11,580 | Department of Statistics, Surveys and Censuses (Paraguay). Paraguay Integrated Household Survey 1997-1998. AsunciÃ³n, Paraguay: Department of Statistics, Surveys and Censuses (Paraguay). |
| Paraguay | 10350 | sbh | 20,082 | Department of Statistics, Surveys and Censuses (Paraguay). Paraguay Integrated Household Survey 2000-2001. AsunciÃ³n, Paraguay: Department of Statistics, Surveys and Censuses (Paraguay). |
| Paraguay | 227167 | sbh | 272,548 | Minnesota Population Center, Paraguay Department of Statistics, Surveys and Censuses. Paraguay Population and Housing Census 2002 from the Integrated Public Use Microdata Series, International: [Machine-readable database]. Minneapolis: University of Minnesota, 2015. |
| Paraguay | 10370 | cbh | 14,799 | Division of Reproductive Health-Centers for Disease Control and Prevention (CDC). (2005): Paraguay Reproductive Health Survey 2004. AsunciÃ³n, Paraguay, Paraguayan Center for Population Studies (CEPEP). |
| Paraguay | 27525 | cbh | 11,367 | Paraguay Center for Population Studies (CEPEP). Paraguay Reproductive Health Survey 2008. AsunciÃ³n, Paraguay: Paraguayan Center for Population Studies (CEPEP). |
| Paraguay | 324470 | cbh | 14,355 | General Directorate of Statistics, Surveys and Censuses (DGEEC)(Paraguay), Ministry of Public Health and Social Welfare (Paraguay), United Nations Children's Fund (UNICEF). Paraguay Multiple Indicator Cluster Survey 2016. New York, United States: United Nations Children's Fund (UNICEF), 2017. |
| Peru | 10460 | sbh | 10,468 | Instituto CuÃ¡nto. Peru National Living Standards Measurement Survey 2000. Lima, Peru: Instituto CuÃ¡nto. |
| Peru | 41267 | sbh | 1,255,851 | National Institute of Statistics and Informatics (INEI) (Peru), Minnesota Population Center. Peru National Population and Housing Census 2007 from the Integrated Public Use Microdata Series, International: [Machine-readable database]. Minneapolis: University of Minnesota. |
| Peru | 146860 | cbh | 44,725 | ICF International, National Institute of Statistics and Informatics (INEI) (Peru). Peru Continuous Demographic and Health Survey 2013. Lima, Peru: National Institute of Statistics and Informatics (INEI) (Peru), 2014. |
| Peru | 303663 | cbh | 74,559 | National Institute of Statistics and Informatics (INEI) (Peru). Peru Demographic and Family Health Survey 2015. Lima, Peru: National Institute of Statistics and Informatics (INEI) (Peru), 2017. |
| Peru | 303664 | cbh | 67,481 | National Institute of Statistics and Informatics (INEI) (Peru). Peru Demographic and Family Health Survey 2016. Lima, Peru: National Institute of Statistics and Informatics (INEI) (Peru), 2017. |
| Philippines | 135803 | cbh | 99,962 | ICF International, National Statistics Office (Philippines). Philippines Demographic and Health Survey 2011. |
| Rwanda | 42432 | sbh | 518,045 | National Census Commission (Rwanda), Minnesota Population Center. Rwanda Population and Housing Census 2002 from the Integrated Public Use Microdata Series, International: [Machine-readable database]. Minneapolis: University of Minnesota. |
| Rwanda | 11324 | sbh | 21,025 | National Institute of Statistics of Rwanda (NISR), Oxford Policy Management. Rwanda Integrated Living Conditions Survey 2005-2006. Kigali, Rwanda: National Institute of Statistics of Rwanda (NISR). |
| Rwanda | 218773 | sbh | 547,998 | National Institute of Statistics of Rwanda. Rwanda Population and Housing Census 2012. Kigali, Rwanda: National Institute of Statistics of Rwanda, 2015. |
| Rwanda | 77391 | sbh | 11,726 | ICF International, Ministry of Health (Rwanda). Rwanda Malaria Indicator Survey 2012-2013. Fairfax, United States: ICF International, 2014. |
| Sao Tome and Principe | 27055 | sbh | 7,765 | National Institute of Statistics (Sao Tome and Principe), United Nations Children's Fund (UNICEF). Sao Tome and Principe Multiple Indicator Cluster Survey 2000. New York, United States: United Nations Children's Fund (UNICEF). |
| Sao Tome and Principe | 214640 | cbh | 7,492 | Global Fund to Fight Aids Tuberculosis and Malaria (GFATM), ICF International, National Center for Endemic Diseases (CNE) (Sao Tome and Principe), National Institute of Statistics (Sao Tome and Principe), United Nations Children's Fund (UNICEF), United Nations Development Programme (UNDP). Sao Tome and Principe Multiple Indicator Cluster Survey 2014. New York, United States: United Nations Children's Fund (UNICEF), 2016. |
| Senegal | 20786 | cbh | 51,506 | Groupe SERDHA, Macro International, Inc, Ministry of Health and Prevention (Senegal). Senegal Demographic and Health Survey 1999-2000. Calverton, United States: Macro International, Inc. |
| Senegal | 43142 | sbh | 564,269 | Directorate of Forecasting and Statistics (Senegal), Minnesota Population Center. Senegal General Population and Housing Census 2002 from the Integrated Public Use Microdata Series, International: [Machine-readable database]. Minneapolis: University of Minnesota. |
| Senegal | 11516 | sbh | 18,520 | Macro International, Inc, Research Center for Human Development (Senegal). Senegal Malaria Indicator Survey 2006. Calverton, United States: Macro International, Inc. |
| Sierra Leone | 11639 | sbh | 17,032 | Central Statistics Office (Sierra Leone), United Nations Children's Fund (UNICEF). Sierra Leone Multiple Indicator Cluster Survey 2000. New York, United States: United Nations Children's Fund (UNICEF). |
| Sierra Leone | 11661 | sbh | 344,320 | Statistics Sierra Leone and Minnesota Population Center. Sierra Leone Population and Housing Census 2004 from the Integrated Public Use Microdata Series, International: [Machine-readable database]. Minneapolis: University of Minnesota, 2011. |
| Sierra Leone | 11649 | sbh | 28,284 | United Nations Children's Fund (UNICEF), Statistics Sierra Leone. Sierra Leone Multiple Indicator Cluster Survey 2005. New York, United States: United Nations Children's Fund (UNICEF). |
| Sierra Leone | 76700 | sbh | 39,257 | Statistics Sierra Leone, United Nations Children's Fund (UNICEF). Sierra Leone Multiple Indicator Cluster Survey 2010. New York, United States: United Nations Children's Fund (UNICEF). |
| Sierra Leone | 286773 | sbh | 28,463 | Catholic Relief Services (CRS), College of Medicine and Allied Health Sciences, University of Sierra Leone (COMAHS), ICF International, Roll Back Malaria Partnership, Statistics Sierra Leone. Sierra Leone Malaria Indicator Survey 2016. Fairfax, United States: ICF International, 2017. |
| Somalia | 11774 | cbh | 20,034 | Pan Arab Project for Family Health (PAPFAM), United Nations Children's Fund (UNICEF). Somalia Multiple Indicator Cluster Survey 2006. New York, United States: United Nations Children's Fund (UNICEF). |
| South Africa | 105306 | cbh | 65,741 | Central Statistical Service (South Africa). South Africa October Household Survey 1994. |
| South Africa | 106684 | cbh | 62,706 | Central Statistical Service (South Africa). South Africa October Household Survey 1995. |
| South Africa | 106686 | cbh | 104,873 | Central Statistical Service (South Africa). South Africa October Household Survey 1997. |
| South Africa | 43152 | sbh | 1,799,625 | Statistics South Africa, Minnesota Population Center. South Africa Census 2001 from the Integrated Public Use Microdata Series, International: [Machine-readable database]. Minneapolis: University of Minnesota. |
| South Africa | 43158 | sbh | 469,555 | Statistics South Africa, Minnesota Population Center. South Africa Community Survey 2007 from the Integrated Public Use Microdata Series, International: [Machine-readable database]. Minneapolis: University of Minnesota. |
| South Africa | 280803 | sbh | 1,247,106 | Statistics South Africa. South Africa Community Survey 2016. Pretoria, South Africa: Statistics South Africa, 2016. |
| South Sudan | 106548 | sbh | 337,728 | Minnesota Population Center, Southern Sudan Centre for Census, Statistics and Evaluation. Sudan - South Sudan Population and Housing Census 2008 from the Integrated Public Use Microdata Series, International: [Machine-readable database]. Minneapolis: University of Minnesota, 2013 |
| Sudan | 24143 | cbh | 77,520 | Ministry of Health (Southern Sudan), Federal Ministry of Health (Sudan), Southern Sudan Centre for Census, Statistics and Evaluation (SSCCSE), Central Bureau of Statistics (Sudan). Sudan Family Health Survey 2006. |
| Sudan | 43167 | sbh | 2,810,742 | National Population Census Council (Sudan), Central Bureau of Statistics (Sudan), Southern Sudan Centre for Census, Statistics and Evaluation (SSCCSE), Minnesota Population Center. Sudan Population and Housing Census 2008 from the Integrated Public Use Microdata Series, International: [Machine-readable database]. Minneapolis: University of Minnesota, 2011. |
| Sudan | 153643 | cbh | 47,092 | Central Bureau of Statistics (Sudan), Ministry of Health (South Sudan). Sudan - North Multiple Indicator Cluster Survey 2010. New York, United States: United Nations Children's Fund (UNICEF), 2015. |
| Sudan | 200617 | cbh | 52,245 | Central Bureau of Statistics (Sudan), Federal Ministry of Health (Sudan), United Nations Children's Fund (UNICEF). Sudan Multiple Indicator Cluster Survey 2014. New York, United States: United Nations Children's Fund (UNICEF), 2016. |
| Suriname | 12280 | sbh | 8,481 | General Bureau of Statistics (Suriname), Pan American Health Organization (PAHO), United Nations Children's Fund (UNICEF), United Nations Development Programme (UNDP). Suriname Multiple Indicator Cluster Survey 1999-2000. New York, United States: United Nations Children's Fund (UNICEF). |
| Suriname | 12289 | sbh | 10,503 | General Statistical Office (Suriname), United Nations Children's Fund (UNICEF). Suriname Multiple Indicator Cluster Survey 2006. New York, United States: United Nations Children's Fund (UNICEF). |
| Swaziland | 12320 | sbh | 13,812 | Central Statistical Office (Swaziland), United Nations Children's Fund (UNICEF). Swaziland Multiple Indicator Cluster Survey 2000. New York, United States: United Nations Children's Fund (UNICEF). |
| Swaziland | 30325 | cbh | 9,805 | Central Statistical Office (Swaziland), United Nations Children's Fund (UNICEF). Swaziland Multiple Indicator Cluster Survey 2010. New York, United States: United Nations Children's Fund (UNICEF). |
| Swaziland | 200707 | cbh | 9,830 | Central Statistical Office (Swaziland), United Nations Children's Fund (UNICEF), United Nations Educational, Scientific and Cultural Organization (UNESCO), United Nations Population Fund (UNFPA). Swaziland Multiple Indicator Cluster Survey 2014. New York, United States: United Nations Children's Fund (UNICEF), 2016. |
| Syria | 12399 | sbh | 55,015 | United Nations Children's Fund (UNICEF), Central Bureau of Statistics (Syria), Ministry of Health (Syria), Pan Arab Project for Family Health (PAPFAM). Syria Multiple Indicator Cluster Survey 2006. New York, United States: United Nations Children's Fund (UNICEF). |
| Tajikistan | 12455 | cbh | 8,527 | National State Statistical Agency (Tajikistan), World Bank. Tajikistan Living Standards Measurement Survey 1999. |
| Tajikistan | 12595 | sbh | 16,393 | National State Statistical Agency (Tajikistan), United Nations Children's Fund (UNICEF). Tajikistan Multiple Indicator Cluster Survey 2000 . New York, United States: United Nations Children's Fund (UNICEF). |
| Tajikistan | 12489 | cbh | 13,458 | National State Statistical Agency (Tajikistan), World Bank. Tajikistan Living Standards Measurement Survey 2003. |
| Tajikistan | 12608 | sbh | 23,127 | United Nations Children's Fund (UNICEF), State Committee on Statistics of the Republic of Tajikistan. Tajikistan Multiple Indicator Cluster Survey 2005. New York, United States: United Nations Children's Fund (UNICEF). |
| Tanzania | 43212 | sbh | 2,664,423 | National Bureau of Statistics (Tanzania), Minnesota Population Center. Tanzania Population and Housing Census 2002 from the Integrated Public Use Microdata Series, International: [Machine-readable database]. Minneapolis: University of Minnesota. |
| Tanzania | 12630 | sbh | 33,014 | National Bureau of Statistics (Tanzania), ORC Macro, Tanzania Commission for AIDS (TACAIDS). Tanzania AIDS Indicator Survey 2003-2004. Calverton, United States: ORC Macro. |
| Tanzania | 12644 | cbh | 27,511 | Macro International, Inc, National Bureau of Statistics (Tanzania), Office of Chief Government Statistician (OCGS-Zanzibar), Tanzania Commission for AIDS (TACAIDS), Zanzibar AIDS Commission (ZAC). Tanzania HIV/AIDS and Malaria Indicator Survey 2007-2008. Calverton, United States: Macro International, Inc. |
| Tanzania | 77395 | sbh | 32,522 | ICF International, National Bureau of Statistics (Tanzania), Office of Chief Government Statistician (OCGS-Zanzibar), Tanzania Commission for AIDS (TACAIDS), Zanzibar AIDS Commission (ZAC). Tanzania AIDS Indicator Survey 2011-2012. Fairfax, United States: ICF International, 2013. |
| Tanzania | 294725 | sbh | 3,225,395 | National Bureau of Statistics (Tanzania), Minnesota Population Center. Tanzania Population and Housing Census 2012 from the Integrated Public Use Microdata Series, International. Minneapolis: University of Minnesota, 2017. |
| Thailand | 43231 | sbh | 213,031 | National Statistical Office (Thailand), Minnesota Population Center. Thailand Population and Housing Census 2000 from the Integrated Public Use Microdata Series, International: [Machine-readable database]. Minneapolis: University of Minnesota. |
| Thailand | 12732 | sbh | 48,610 | National Statistical Office (Thailand), United Nations Children's Fund (UNICEF). Thailand Multiple Indicator Cluster Survey 2005-2006. New York, United States: United Nations Children's Fund (UNICEF). |
| Thailand | 148649 | sbh | 30,853 | College of Population Studies, Chulalongkorn University (Thailand), Institute for Population and Social Research, Mahidol University (Thailand), International Health Policy Program (Thailand), Ministry of Education (Thailand), Ministry of Public Health (Thailand), Ministry of Social Development and Human Security (MSDHS) (Thailand), National Health Security Office (Thailand), National Statistical Office (Thailand), Thai Health Promotion Foundation, United Nations Children's Fund (UNICEF). Thailand Multiple Indicator Cluster Survey 2012. New York, United States: United Nations Children's Fund (UNICEF), 2016. |
| The Gambia | 3922 | sbh | 16,875 | Central Statistics Department (Gambia), United Nations Children's Fund (UNICEF). Gambia Multiple Indicator Cluster Survey 2000. New York, United States: United Nations Children's Fund (UNICEF). |
| The Gambia | 3935 | sbh | 27,475 | Gambia Bureau of Statistics (GBOS), United Nations Children's Fund (UNICEF). Gambia Multiple Indicator Cluster Survey 2005-2006. New York, United States: United Nations Children's Fund (UNICEF). |
| Timor-Leste | 20888 | cbh | 17,889 | ACIL Australia Pty Ltd., Australian National University, Ministry of Health (Timor-Leste), National Statistics Directorate (Timor-Leste), University of Newcastle (Australia). Timor-Leste Demographic and Health Survey 2003. Newcastle, Australia: University of Newcastle (Australia). |
| Togo | 12896 | sbh | 17,832 | Directorate General of Statistics and National Accounting (Togo), United Nations Children's Fund (UNICEF). Togo Multiple Indicator Cluster Survey 2006. New York, United States: United Nations Children's Fund (UNICEF). |
| Togo | 40021 | sbh | 18,954 | Directorate General of Statistics and National Accounting (Togo), United Nations Children's Fund (UNICEF). Togo Multiple Indicator Cluster Survey 2010. New York, United States: United Nations Children's Fund (UNICEF). |
| Trinidad and Tobago | 12940 | sbh | 6,445 | Central Statistical Office (Trinidad and Tobago), United Nations Children's Fund (UNICEF). Trinidad and Tobago Multiple Indicator Cluster Survey 2000. New York, United States: United Nations Children's Fund (UNICEF). |
| Trinidad and Tobago | 12950 | sbh | 6,551 | Central Statistical Office (Trinidad and Tobago) and United Nations Children's Fund (UNICEF). Trinidad and Tobago Multiple Indicator Cluster Survey 2006. New York, United States: United Nations Children's Fund (UNICEF). |
| Trinidad and Tobago | 294807 | sbh | 35,604 | Central Statistical Office (Trinidad and Tobago), Minnesota Population Center. Trinidad and Tobago Population and Housing Census 2011 from the Integrated Public Use Microdata Series, International [Machine-readable database]. Minneapolis: University of Minnesota, 2017. |
| Tunisia | 76709 | cbh | 13,569 | Ministry of Regional Development and Planning (Tunisia), National Institute of Statistics (Tunisia), United Nations Children's Fund (UNICEF). Tunisia Multiple Indicator Cluster Survey 2011-2012. New York, United States: United Nations Children's Fund (UNICEF), 2014. |
| Turkey | 56509 | sbh | 1,638,302 | Minnesota Population Center, State Institute of Statistics (Turkey). Turkey Population Census 2000 from the Integrated Public Use Microdata Series, International: [Machine-readable database]. Minneapolis: University of Minnesota, 2012. |
| Turkey | 32421 | cbh | 19,678 | Institute of Population Studies, Hacettepe University, Ministry of Health (Turkey), State Planning Organization (Turkey), Turkish Statistical Institute. Turkey Demographic and Health Survey 2008. Ankara, Turkey: Institute of Population Studies, Hacettepe University. |
| Turkey | 90439 | sbh | 55,121 | Istanbul University, Ministry of Health (Turkey), Turkish Statistical Institute. Turkey Infant and Under-5 Mortality Survey 2011. |
| Turkmenistan | 13064 | sbh | 12,070 | Ministry of Health and Medical Industry (Turkmenistan), United Nations Children's Fund (UNICEF). Turkmenistan Multiple Indicator Cluster Survey 2006. New York, United States: United Nations Children's Fund (UNICEF), 2016. |
| Turkmenistan | 264583 | cbh | 12,800 | State Committee on Statistics of Turkmenistan, United Nations Children's Fund (UNICEF). Turkmenistan Multiple Indicator Cluster Survey 2015-2016. New York, United States: United Nations Children's Fund (UNICEF), 2017. |
| Uganda | 43328 | sbh | 1,811,659 | Uganda Bureau of Statistics, Minnesota Population Center. Uganda Population and Housing Census 2002 from the Integrated Public Use Microdata Series, International: [Machine-readable database]. Minneapolis: University of Minnesota. |
| Uganda | 81004 | sbh | 9,404 | Uganda Bureau of Statistics. Uganda Living Standards Measurement Survey - Integrated Survey on Agriculture 2009-2010. Washington DC, United States: World Bank. |
| Uganda | 55973 | sbh | 68,086 | Centers for Disease Control and Prevention (CDC), ICF Macro, Ministry of Health (Uganda), Uganda Bureau of Statistics, Uganda Virus Research Institute. Uganda AIDS Indicator Survey 2011. Calverton, United States: ICF Macro. |
| Uganda | 157065 | sbh | 17,128 | ICF International, National Malaria Control Program, Ministry of Health (Uganda), Uganda Bureau of Statistics. Uganda Malaria Indicator Survey 2014-2015. Fairfax, United States: ICF International, 2015. |
| Ukraine | 13218 | cbh | 8,144 | Division of Reproductive Health-Centers for Disease Control and Prevention (CDC) and Kiev International Institute of Sociology. (2001) Ukraine Reproductive Health Survey 1999. Atlanta, United States: Centers for Disease Control and Prevention (CDC). |
| Ukraine | 13197 | sbh | 7,872 | United Nations Children's Fund (UNICEF). Ukraine Multiple Indicator Cluster Survey 2005. New York, United States: United Nations Children's Fund (UNICEF). |
| Ukraine | 132739 | sbh | 10,715 | StatInform Consulting, State Statistics Service (Ukraine), Ukrainian Center for Social Reforms (UCSR), United Nations Children's Fund (UNICEF). Ukraine Multiple Indicator Cluster Survey 2012. New York, United States: United Nations Children's Fund (UNICEF), 2014. |
| Uruguay | 56577 | sbh | 95,208 | Minnesota Population Center, National Institute of Statistics (Uruguay). Uruguay Extended National Household Survey 2006 from the Integrated Public Use Microdata Series, International: [Machine-readable database]. Minneapolis: University of Minnesota, 2012. |
| Uruguay | 151322 | sbh | 106,698 | National Institute of Statistics (Uruguay), Minnesota Population Center. Uruguay Census 2011 from the Integrated Public Use Microdata Series, International: [Machine-readable database]. Minneapolis: University of Minnesota. |
| Uzbekistan | 13436 | sbh | 16,770 | United Nations Children's Fund (UNICEF), Ministry of Macroeconomics and Statistics (Uzbekistan). Uzbekistan Multiple Indicator Cluster Survey 2000. New York, United States: United Nations Children's Fund (UNICEF). |
| Uzbekistan | 21039 | cbh | 11,607 | Analytical and Information Center of the Ministry of Health of Uzbekistan, Macro International, Inc, Ministry of Macroeconomics and Statistics (Uzbekistan). Uzbekistan Special Demographic and Health Survey 2002. Calverton, United States: Macro International, Inc. |
| Uzbekistan | 13445 | sbh | 26,751 | United Nations Children's Fund (UNICEF), State Committee of the Republic of Uzbekistan on Statistics. Uzbekistan Multiple Indicator Cluster Survey 2006. New York, United States: United Nations Children's Fund (UNICEF). |
| Vanuatu | 13465 | sbh | 6,316 | Ministry of Health (Vanuatu), United Nations Children's Fund (UNICEF). Vanuatu Multiple Indicator Cluster Survey 2007-2008. New York, United States: United Nations Children's Fund (UNICEF). |
| Venezuela | 43412 | sbh | 1,161,057 | National Institute of Statistics (Venezuela), Minnesota Population Center. Venezuela Population and Housing Census 2002 from the Integrated Public Use Microdata Series, International: [Machine-readable database]. Minneapolis: University of Minnesota. |
| Vietnam | 43718 | sbh | 1,103,904 | General Statistics Office (Viet Nam), Minnesota Population Center. Viet Nam Population and Housing Census 1999 from the Integrated Public Use Microdata Series, International: [Machine-readable database]. Minneapolis: University of Minnesota. |
| Vietnam | 13708 | sbh | 17,570 | General Statistics Office (Viet Nam), United Nations Children's Fund (UNICEF). Vietnam Multiple Indicator Cluster Survey 2000. New York, United States: United Nations Children's Fund (UNICEF). |
| Vietnam | 13544 | sbh | 20,964 | General Statistics Office (Viet Nam), National Institute of Hygiene and Epidemiology (Viet Nam), ORC Macro. Vietnam AIDS Indicator Survey 2005. Calverton, United States: Macro International, Inc. |
| Vietnam | 13719 | sbh | 16,447 | General Statistics Office (Viet Nam), United Nations Children's Fund (UNICEF). Vietnam Multiple Indicator Cluster Survey 2006. New York, United States: United Nations Children's Fund (UNICEF). |
| Vietnam | 43726 | sbh | 6,004,427 | General Statistics Office (Viet Nam), Minnesota Population Center. Viet Nam Population and Housing Census 2009 from the Integrated Public Use Microdata Series, International: [Machine-readable database]. Minneapolis: University of Minnesota. |
| Vietnam | 57999 | sbh | 18,127 | General Statistics Office (Viet Nam), United Nations Children's Fund (UNICEF). Vietnam Multiple Indicator Cluster Survey 2010-2011. New York, United States: United Nations Children's Fund (UNICEF). |
| Vietnam | 152735 | cbh | 15,479 | General Statistics Office (Viet Nam), United Nations Children's Fund (UNICEF). Vietnam Multiple Indicator Cluster Survey 2013-2014. New York, United States: United Nations Children's Fund (UNICEF), 2015. |
| Yemen | 13795 | sbh | 54,378 | Central Statistical Organization (Yemen), League of Arab States, Ministry of Public Health and Population (Yemen), Pan Arab Project for Family Health (PAPFAM). Yemen Family Health Survey 2003. |
| Yemen | 13816 | cbh | 17,213 | Ministry of Health (Yemen) and United Nations Children's Fund (UNICEF). Yemen Multiple Indicator Cluster Survey 2006. New York, United States: United Nations Children's Fund (UNICEF). |
| Zambia | 151325 | sbh | 602,546 | Central Statistical Office (Zambia), Minnesota Population Center. Zambia Census 2000 from the Integrated Public Use Microdata Series, International: [Machine-readable database]. Minneapolis: University of Minnesota. |
| Zambia | 151326 | sbh | 768,988 | Central Statistical Office (Zambia), Minnesota Population Center. Zambia Census 2010 from the Integrated Public Use Microdata Series, International: [Machine-readable database]. Minneapolis: University of Minnesota. |
| Zimbabwe | 35493 | cbh | 23,716 | Central Statistical Office (Zimbabwe). Zimbabwe Multiple Indicator Monitoring Survey 2009. New York, United States: United Nations Children's Fund (UNICEF). |
| Zimbabwe | 152720 | cbh | 32,285 | United Nations Children's Fund (UNICEF), Zimbabwe National Statistics Agency. Zimbabwe Multiple Indicator Cluster Survey 2014. New York, United States: United Nations Children's Fund (UNICEF), 2015. |
