# Supplementary material for: Development and validation of a new method for indirect estimation of neonatal, infant, and child mortality trends using summary birth histories
Source: PLoS Med. 2018 Oct 31;15(10):e1002687. doi: 10.1371/journal.pmed.1002687 (PMC6209133; doi:10.1371/journal.pmed.1002687)
Supplement: S3 Table — MAPE, median absolute percentage error; ME, mean error; MRE, median relative error; q¯, average estimated mortality probability; R2, coefficient of determination; SDE, standard deviation of the errors. (DOCX) [file pmed.1002687.s004.docx]

| ***Age-bin*** | ${\bar{\boldsymbol{q}}}_{\boldsymbol{a}}$ | ***ME*** | ***SDE*** | ***MRE*** | ***MAPE*** | $\boldsymbol{R}^{\boldsymbol{2}}$ |
| --- | --- | --- | --- | --- | --- | --- |
| ***NN*** | 0.030 | 0.002 | 0.010 | 1.05 | 20.0 | 0.53 |
| ***PNN1*** | 0.017 | 0.000 | 0.007 | 1.05 | 26.5 | 0.62 |
| ***PNN2*** | 0.015 | 0.000 | 0.007 | 1.04 | 29.0 | 0.66 |
| ***1yr*** | 0.015 | 0.000 | 0.008 | 1.06 | 30.0 | 0.72 |
| ***2yr*** | 0.011 | 0.000 | 0.006 | 0.98 | 28.7 | 0.73 |
| ***3yr*** | 0.007 | 0.000 | 0.005 | 0.94 | 31.5 | 0.61 |
| ***4yr*** | 0.004 | 0.000 | 0.004 | 0.92 | 38.0 | 0.43 |
| ***5q0*** | 0.094 | 0.002 | 0.017 | 1.04 | 11.8 | 0.91 |
