# Supplementary figures and images for: Development and validation of a new method for indirect estimation of neonatal, infant, and child mortality trends using summary birth histories
Source: PLoS Med. 2018 Oct 31;15(10):e1002687. doi: 10.1371/journal.pmed.1002687 (PMC6209133; doi:10.1371/journal.pmed.1002687)

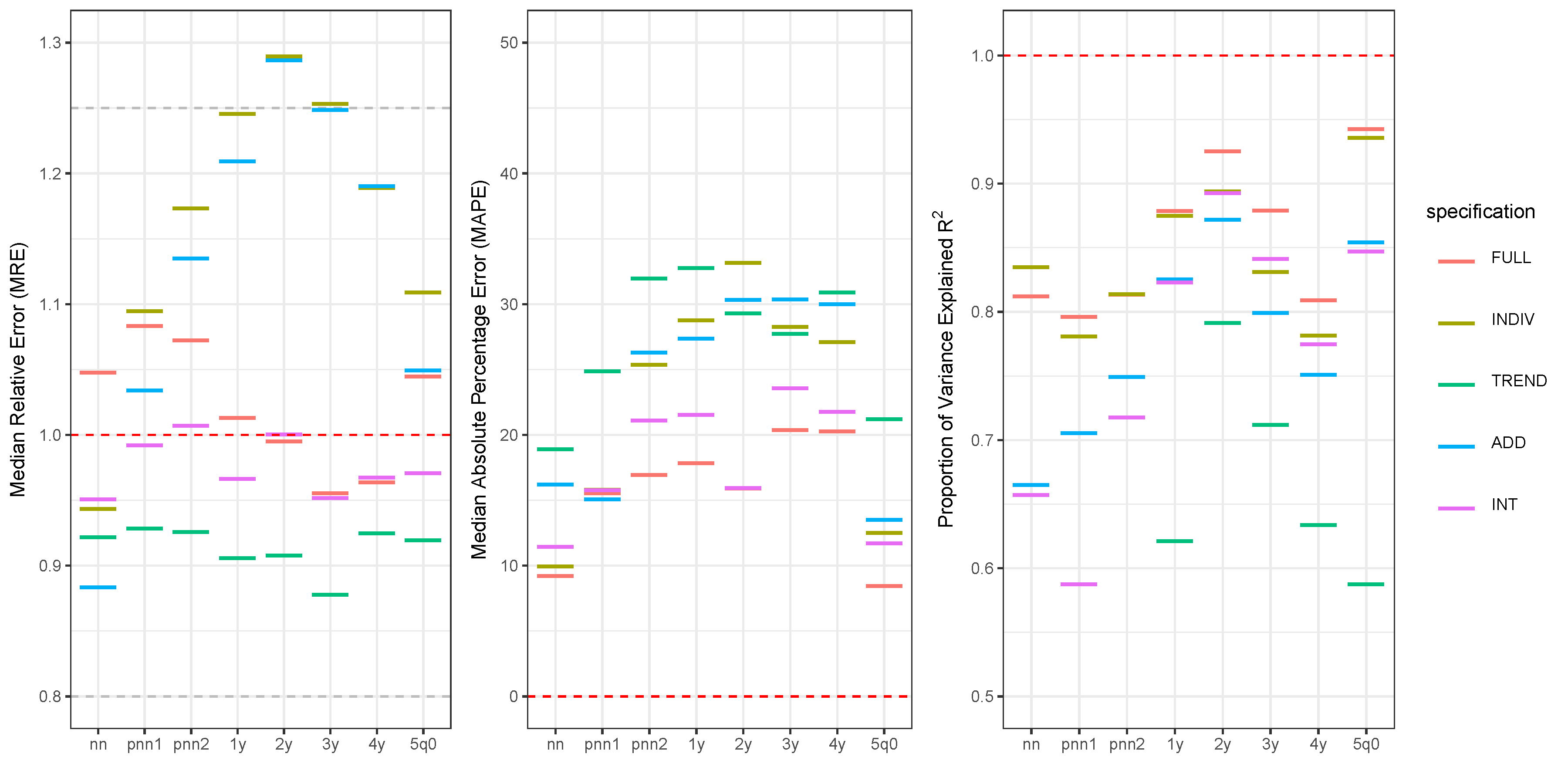

Supplement: S1 Fig — We tested and compared the following models: (FULL) The full model specification described in the Methods section. (INT) All covariates from the full model specified with no smooths; variable interactions in smooths replaced with interaction terms. (ADD) All covariates from the full model but specified with no smooths or interactions; all variables included additively and untransformed. (TREND) Only the year-SDI smooth included, with no individual covariates. (INDIV) Only the individual-level covariates used, with no year-SDI trend included. This figure shows metrics of predictive validity for these different specifications. The full model does reliably best across all metrics, with only a few exceptions. Notably, full specification is outperformed in bias (MRE) for several age groups by the interaction-only model. The full model generally outperforms all other models. These findings indicate that the predictive gains from using the more flexible (yet complex) GAM approach were appropriate here. The TREND specification does reliably worst across all metrics, indicating that there was value in including individual-level time-varying covariates. From left: MRE, MAPE, and R2. Dashed red lines are the target values for perfect fit for each metric. Note that if metrics for certain specifications are missing, they are outside of the plotted range and can be considered very poorly performing. GAM, generalized additive model; MAPE, median absolute percentage error; MRE, median relative error; SDI, Socio-demographic Index. (TIF) [file pmed.1002687.s005.tif]

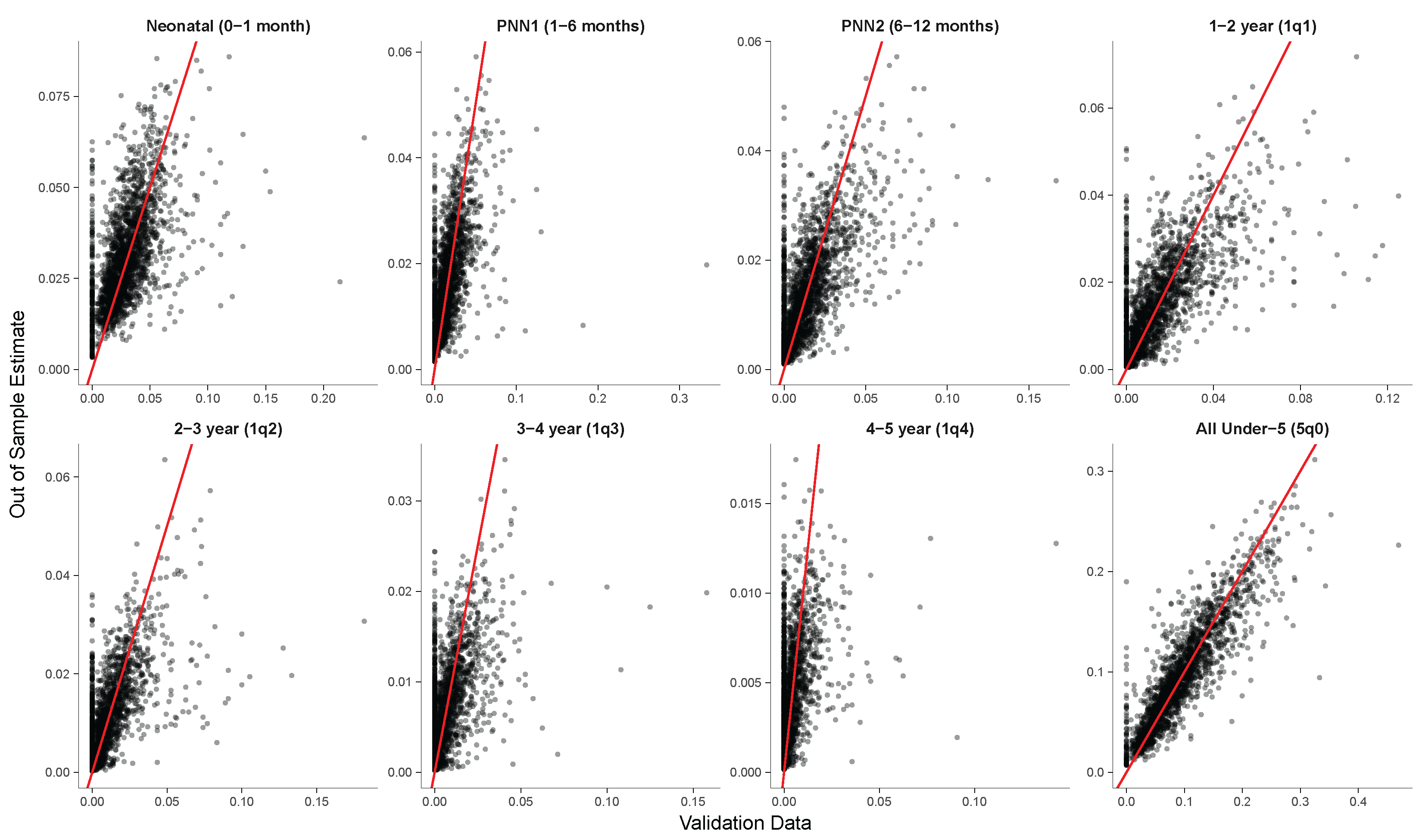

Supplement: S2 Fig — Each point represents an admin1-age mortality estimate (qa,adm1,yr) for each held-out survey. Red line indicates unity. loess, local regression. (TIF) [file pmed.1002687.s006.tif]

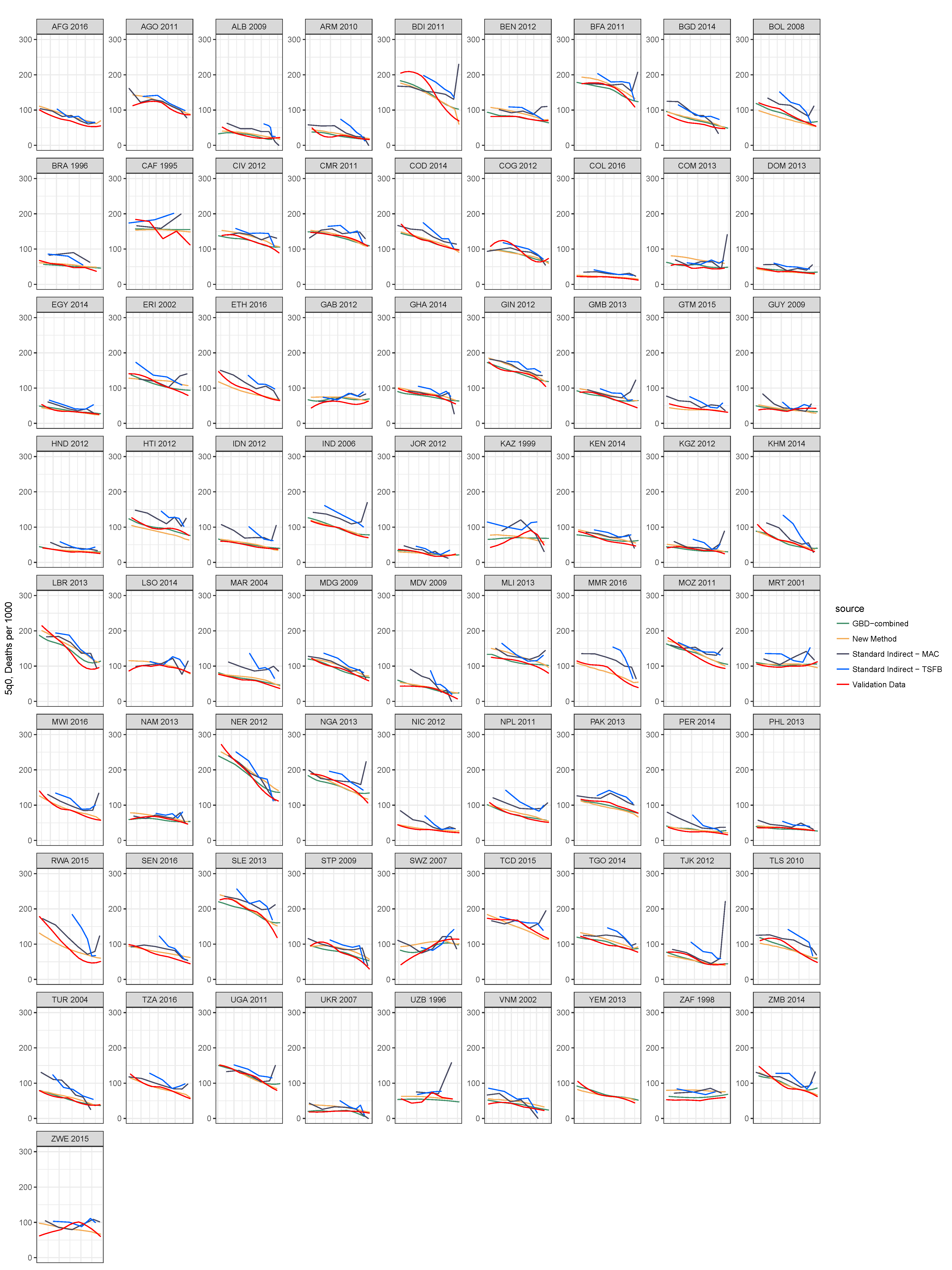

Supplement: S3 Fig — Sixty of the surveys had GBD-combined estimates available from the GBD mortality database. Trends begin at the year of the survey and go back 15 years or until 1990. Red lines indicate validation data (smoothed with loess, α = 0.85). GBD, Global Burden of Disease; loess, local regression; MAC, maternal age cohort; TSFB, time since first birth. (TIF) [file pmed.1002687.s007.tif]

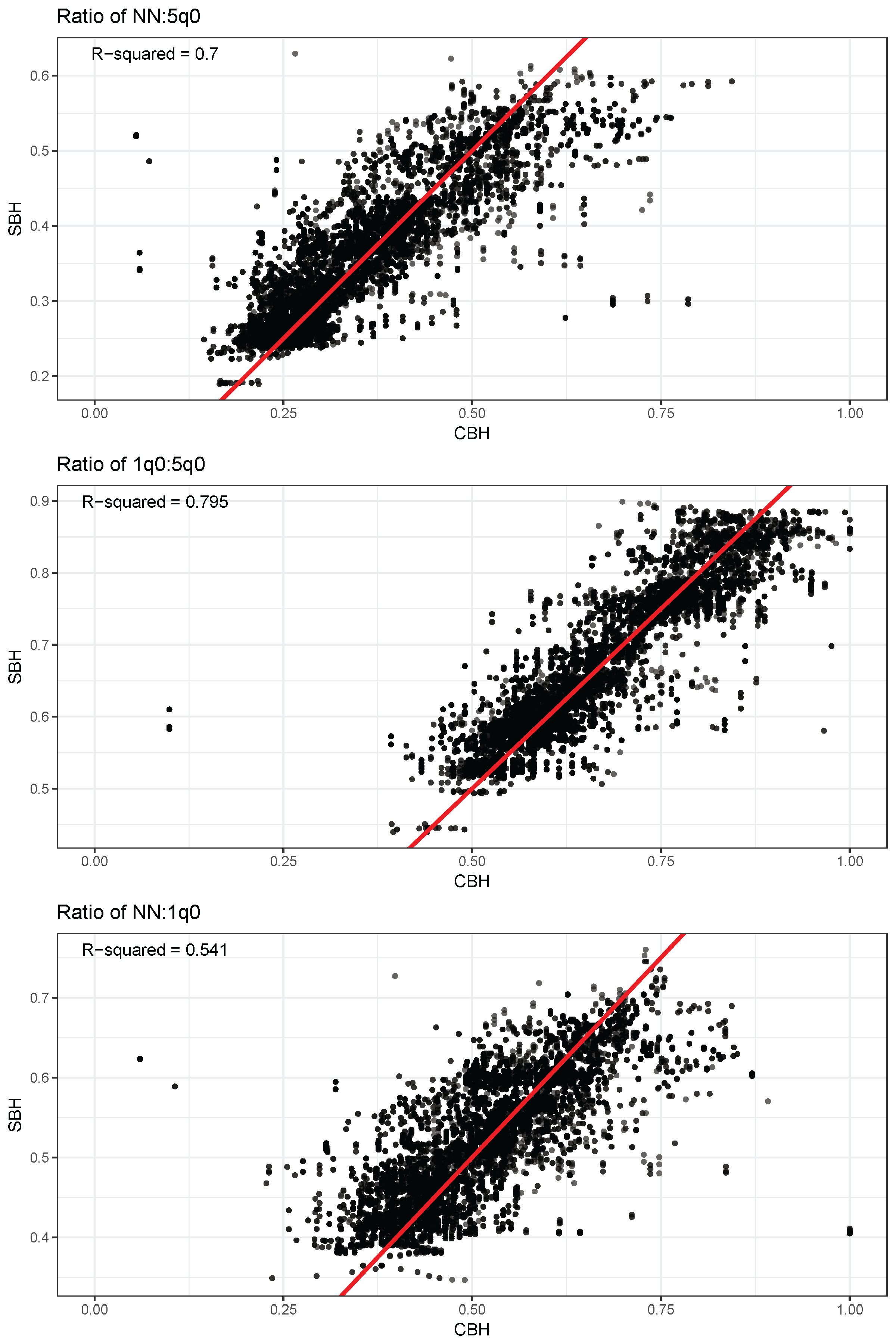

Supplement: S6 Fig — In this figure, we show scatterplots of the relationships between NN and under-5 mortality, infant and under-5 mortality, and NN and infant mortality across country-year data pairs from contemporaneous SBH- and CBH-derived estimates. High overall agreement in these ratios adds further support to the predictive validity of this method. Each point is a contemporaneous country-year estimate. Red line indicates unity. CBH, complete birth history; NN, neonatal; SBH, summary birth history. (TIF) [file pmed.1002687.s010.tif]

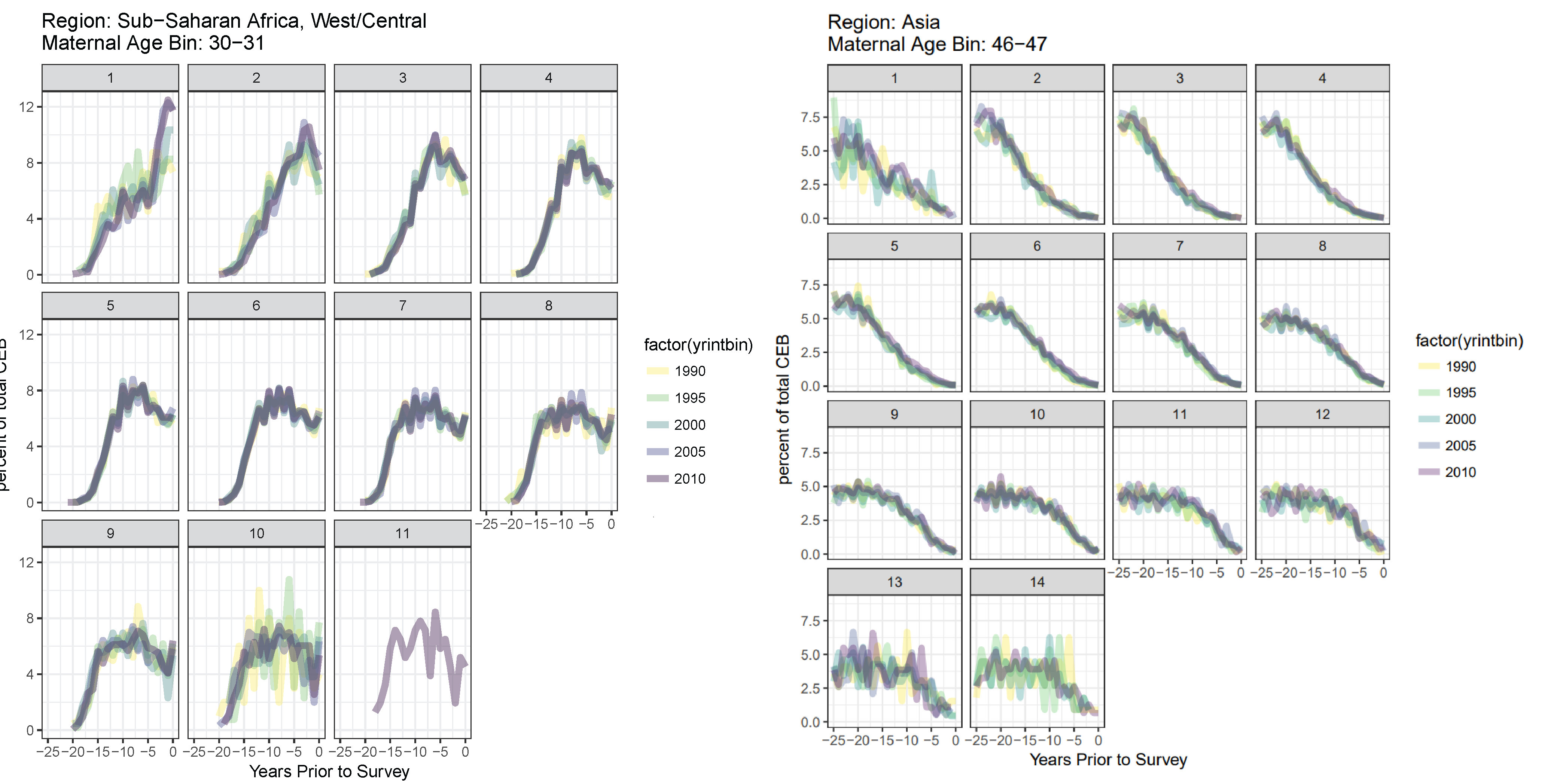

Supplement: S7 Fig — In other words, birth histories are pooled for all mothers from a region with the same CEB, irrespective of year of birth. If distributions changed significantly over time, this could present concern for this analysis. Using the DHS database, we reproduced POB distributions by region, maternal age, CEB, and year of survey (5-year increments). This figure shows two examples of empirical POB distributions made to explore changes in POB over time. Overall, we found very little change in POB distributions over time for most maternal age groups. The most change has occurred for low-fertility (1–2 CEB) older women, as the distributions seem to indicate there has been a trend toward delayed fertility in this group in recent years. Overall, this group represents a very small proportion of the mortality exposure in the data. Noisy distributions in high-fertility women are due to low sample sizes. (a) Sub-Saharan Africa West/Central region plots for women ages 30–31 years. (b) Asia region for women ages 46–47 years. A full set of distributions for all maternal ages and regions can be made available by the authors upon request. CEB, children ever born; DHS, Demographic and Health Surveys; GBD, Global Burden of Disease; MAP, maternal age period; POB, probability of birth. (TIF) [file pmed.1002687.s011.tif]
