# Supplementary material for: Development and validation of a new method for indirect estimation of neonatal, infant, and child mortality trends using summary birth histories
Source: PLoS Med. 2018 Oct 31;15(10):e1002687. doi: 10.1371/journal.pmed.1002687 (PMC6209133; doi:10.1371/journal.pmed.1002687)
Supplement: S4 Fig — Surveys used for out-of-sample validation are labeled with an “X” in Supplementary Table 1. (DOCX) [file pmed.1002687.s008.docx]

UPLOADED HERE: [https://doi.org/10.6084/m9.figshare.7163300.v1](https://doi.org/10.6084/m9.figshare.7163300.v1)
