# Supplementary material for: Development and validation of a new method for indirect estimation of neonatal, infant, and child mortality trends using summary birth histories
Source: PLoS Med. 2018 Oct 31;15(10):e1002687. doi: 10.1371/journal.pmed.1002687 (PMC6209133; doi:10.1371/journal.pmed.1002687)
Supplement: S5 Fig — Trends estimated using the new indirect method for SBH-only data are in blue; direct estimates from CBH surveys are in red. Citations for censuses and surveys used for external validation additional to the training and testing DHS data are listed in S2 Table. CBH, complete birth history; DHS, Demographic and Health Surveys; SBH, summary birth history. (DOCX) [file pmed.1002687.s009.docx]

UPLOADED HERE: [https://doi.org/10.6084/m9.figshare.7163321.v1](https://doi.org/10.6084/m9.figshare.7163321.v1)
